# Emergence and evolution of heterocyte glycolipid biosynthesis enabled specialized nitrogen fixation in cyanobacteria

**DOI:** 10.1073/pnas.2413972122

**Published:** 2025-01-27

**Authors:** Ruth Pérez Gallego, F. A. Bastiaan von Meijenfeldt, Nicole J. Bale, Jaap S. Sinninghe Damsté, Laura Villanueva

**Affiliations:** ^a^Department of Marine Microbiology and Biogeochemistry, Royal Netherlands Institute for Sea Research (NIOZ), Den Burg 1790 AB, The Netherlands; ^b^Department of Earth Sciences, Faculty of Geosciences, Utrecht University, Utrecht 3508 TA, The Netherlands; ^c^Department of Biology, Faculty of Sciences, Utrecht University, Utrecht 3584 CS, The Netherlands

**Keywords:** heterocytous cyanobacteria, nitrogen fixation, heterocyte glycolipid biosynthesis, biosynthetic gene cluster, lipid biomarkers

## Abstract

Heterocytes, specialized cells for nitrogen fixation in cyanobacteria, are surrounded by heterocyte glycolipids (HGs), which contribute to protection of the nitrogenase enzyme from oxygen. Diverse HGs preserve in the sediment and have been widely used as evidence of past nitrogen fixation, and structural variation has been suggested to preserve taxonomic information and reflect paleoenvironmental conditions. Here, by comprehensive HG identification and screening of HG biosynthetic gene clusters throughout cyanobacteria, we reconstruct the convergent evolutionary history of HG structure, in which different clades produce the same HGs. We find that rudimentary HG biosynthetic machinery was already present in cyanobacteria before the emergence of heterocytes for functions unrelated to nitrogen fixation and identify HG analogs produced by specific and distantly related nonheterocytous cyanobacteria. These structurally less complex molecules represent precursors of HGs, suggesting that HGs arose after a genomic reorganization and expansion of ancestral biosynthetic machinery, enabling the rise of cyanobacterial heterocytes in an increasingly oxygenated atmosphere. Our results open a chapter in the potential use of diagenetic products of HGs and HG analogs as fossils for reconstructing the evolution of multicellularity and division of labor in cyanobacteria.

## Significance

The emergence of multicellularity and division of labor in cyanobacteria is a major evolutionary transition and remains elusive due to scarcity of fossil records. We investigate specialized cells used for nitrogen fixation by reconstructing the origin and evolution of molecules unique to their membranes, heterocyte glycolipids (HGs), by combining genomic analysis of ~3,600 cyanobacteria with high-resolution detection of HGs in key cultures. Our results dispute the common use of HGs as taxonomic biomarkers. We reveal the presence of rudimentary HG biosynthetic machinery in cyanobacteria before the emergence of the heterocyte and show that specific nonheterocytous cyanobacteria still produce structurally similar molecules today. Our study helps elucidate how cell specialization evolved in cyanobacteria via neofunctionalization of existing genomic machinery.

Nitrogen-fixing (diazotrophic) cyanobacteria play a major role in nitrogen cycling by transforming N_2_ to biologically available NH_4_^+^ (1–4). To overcome the inhibition by oxygen of the nitrogenase enzyme responsible for nitrogen fixation, diazotrophic cyanobacteria have evolved strategies that separate oxygen-sensitive nitrogen fixation from oxygen-producing photosynthesis, either temporally or spatially (5, 6). One strategy involves the confinement of the nitrogen fixation reaction to heterocytes, specialized nonphotosynthetic cells. Heterocytes are surrounded by a thick-walled cell envelope, composed of polysaccharides and long-chain lipids with sugar headgroups called heterocyte glycolipids (HGs), that limits oxygen diffusion into the cell (7).

**Fig. 1.**
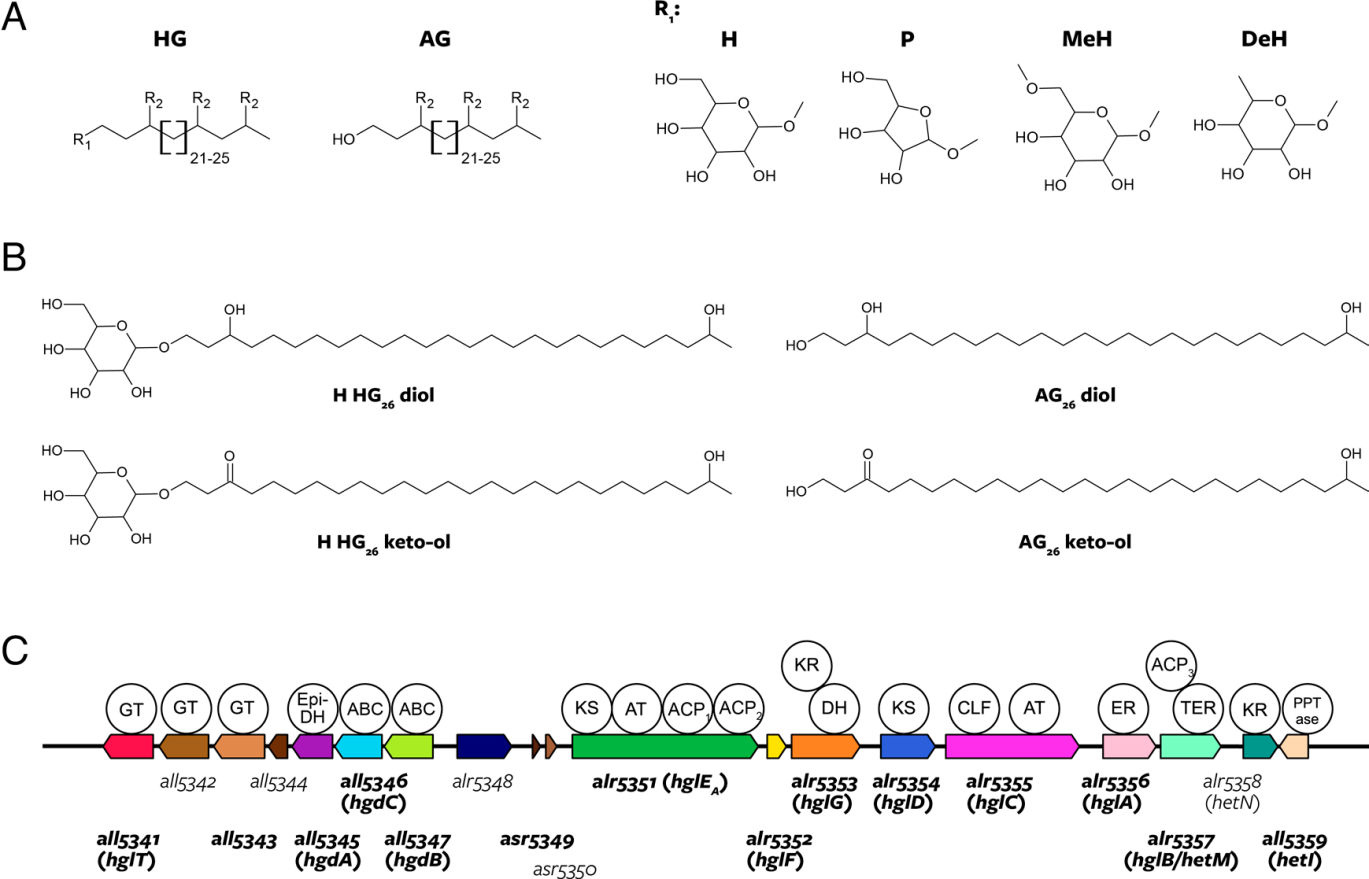
Chemical structure of HGs and schematic representation of the *hgl* island of *Anabaena* sp. PCC 7120 and its most common products. (*A*) HGs consist of a sugar headgroup that is attached to an aglycone (AG). Headgroups (R_1_) are H, hexose; P, pentose; MeH, methyl hexose; and DeH, deoxyhexose. Functional groups on the alkyl chain (R_2_) are H, -OH (alcohol), and =O (ketone). 19 distinct HG structures have previously been identified in heterocytous cyanobacterial cultures and in the environment (8, 10, 11, 20, 23, 24). (*B*) Chemical structure of the most abundant HGs and AGs produced by *Anabaena* sp. PCC 7120. (*C*) Gene cluster in *Anabaena* sp. PCC 7120 containing 19 genes (the *hgl* island), of which 14 have been shown to be involved in HG biosynthesis and transport (in bold). Similar clusters had also been identified in several other heterocyte-forming cyanobacteria (7, 14, 16). The known functions of *Anabaena* sp. PCC 7120 *hgl* island gene products are shown in *SI Appendix,* Table S4, and the complete HG biosynthetic pathway is depicted in *SI Appendix,* Fig. S1. Catalytic domains (circles) are plotted above the genes. Numbered genes depict the genomic naming scheme of *Anabaena* sp. PCC 7120, gene names assigned in previous publications are depicted within brackets. GT, glycosyl transferase; Epi-DH, epimerase-dehydratase; ABC, ATP-binding cassette (ABC) transporter; KS, ketoacyl synthase; AT, acyl transferase; ACP, acyl carrier protein; KR, ketoreductase; DH, dehydratase; CLF, chain length factor; ER, enoyl reductase; TER, thioester reductase; PPTase, phosphopantetheinyltransferase.

HGs detected in cultures of heterocytous cyanobacteria and in the environment are structurally diverse (Fig. 1 *A* and *B*), spanning a variety of alkyl chain lengths (C_26_, C_28_, C_30_, and C_32_), attached to different sugar headgroups [hexoses (C_6_) and pentose (C_5_)], with two or three keto or alcohol groups at fixed positions in their alkyl chain (8–12). Nineteen structurally distinct HGs have been identified to date (13). HGs are biosynthesized by polyketide synthases (PKSs) that extend and reduce a growing acyl chain in successive rounds leading to the formation of a so-called aglycone (AG), which is subsequently attached to a sugar headgroup (Fig. 1 *A* and *B* and *SI Appendix,* Fig. S1) (7, 14–16). Thus, enzymatic differences between cyanobacteria may result in the production of structurally different HGs, which may reflect adaptive differences in membrane impermeabilization to N_2_ and oxygen (10, 17). HGs can be preserved in the sedimentary record and have been used as indicators of past nitrogen fixation by cyanobacteria for example in the early Eocene greenhouse world 49 million years ago (Ma) (18) and during the Cretaceous–Paleogene mass extinction 66 Ma (19), to differentiate specific families and genera of heterocytous cyanobacteria as taxonomic markers (20), and to detect cyanobacterial symbionts of marine diatoms (21). Additionally, specific HGs found in the sedimentary record have been used to reconstruct past surface water temperatures (22). However, despite the relevance of HGs for nitrogen fixation and their potential as biomarkers, knowledge on their structural diversity is limited because to date, only few cyanobacterial cultures have been examined with high-resolution mass spectrometry techniques allowing for comprehensive HG identification, including those present in low abundance.

Fossil evidence and phylogenetic analyses suggest that cyanobacteria capable of heterocyte differentiation arose between 2,450 and 2,100 Ma, around the same time and possibly simultaneously with multicellularity and nitrogen fixation (25–27). Evolutionary pressure for an oxygen-impermeable lipid layer to shelter the nitrogenase enzyme was likely driven by the rising oxygen levels of Earth’s atmosphere around 2,300 Ma (25, 28, 29). Heterocytes have been suggested to be evolutionary related to akinetes (30), spore-like cells produced under stress conditions by some heterocytous cyanobacteria, which are also surrounded by HGs (30, 31).

Here, we reconstruct the acquisition and evolution of the biosynthetic capability to produce HGs by screening ~3,600 cyanobacterial genomes and plasmids, and ~225,000 genomes representing 182 prokaryotic phyla, for key genes involved in HG formation and their deposition in the cell envelope of the heterocyte. We further analyzed the lipid composition of 24 heterocytous and of two nonheterocytous cyanobacterial cultures using high-resolution mass spectrometry to elucidate the connection between biosynthetic capability encoded by the genome and the generated biosynthetic product. Our results reveal that HG structure evolved convergently within the heterocytous cyanobacteria and suggest an origin of HGs from structurally similar molecules still produced by some nonheterocytous cyanobacteria, which are not associated with nitrogen fixation nor akinete formation today.

## Results and Discussion

### Genomic Prediction of HG Biosynthesis in *Cyanobacteriia*.

To investigate the evolution of HG biosynthesis within cyanobacteria, we searched for characteristic genetic signatures in 3,579 publicly available cyanobacterial genomes and plasmids (32) and 14 genomes of heterocytous cyanobacteria that we sequenced as part of this study (of cultures whose lipids were analyzed in this study, or in previous studies, see *SI Appendix*, Tables S1–S3 and Datasets S1 and S2) and focused our analysis on the taxonomic class *Cyanobacteriia*. We screened for the genomic presence and colocalization of 19 protein-coding sequences which are clustered on the genome of *Anabaena* sp. PCC 7120 (Fig. 1*C* and *SI Appendix,* Fig. S1) (14, 33), the well-studied “model” cyanobacterium for heterocyte formation. Fourteen of the encoded proteins have been shown to be involved in the biosynthesis of HGs, and in their export across the inner membrane and cell wall—leading to HG deposition in the cell envelope just outside the outer membrane and beneath the polysaccharide layer (Fig. 1*C* and *SI Appendix*, Table S4) (7, 14–16, 34, 35). This genomic cluster has been called the “*hgl* island” (14, 33) because it contains, among others, several “*hgl*” genes (Fig. 1*C*). Here, we define *hgl* islands as gene clusters containing homologs of at least seven of the 19 queried genes (*SI Appendix*).

To validate our genomic “island” identification approach, we evaluated our results against a subset of cyanobacteria with known morphology (*SI Appendix,* Fig. S2) (36), and analyzed the genomic co-occurrence of two processes taking place in heterocytes, which are both encoded by genomic clusters, HG and nitrogenase biosynthesis (the latter encoded by *nif* genes, *SI Appendix*, Fig. S3 and Tables S4 and S5). This benchmark confirmed that the ability to make heterocytes can be confidently inferred from the genome sequence based on the presence of an *hgl* island as defined here (*SI Appendix*, Table S6).

As PKSs are widely distributed in *Cyanobacteriia* and in other taxonomic groups, we also screened 255,388 genomes representing 182 prokaryotic phyla for gene clusters containing homologs of HG biosynthesis genes (*SI Appendix,* Fig. S4 and Dataset S3). This screen revealed that *hgl* islands are closely related to PKSs involved in polyunsaturated fatty acid (PUFA) biosynthesis in specific genera of *Proteobacteria*, *Bacteroidetes*, and *Chloroflexi*—including the *Proteobacteria Vibrio*, *Shewanella*, *Moritella*, *Colwellia*, and *Psychromonas* (*SI Appendix*) (37, 38), and that all the cyanobacterial *hgl* islands identified here originate from a single ancestral island within *Cyanobacteriia* (*SI Appendix*).

### *Hgl* and *hgl*-Like Islands Are Present Throughout *Cyanobacteriia*.

**Fig. 2.**
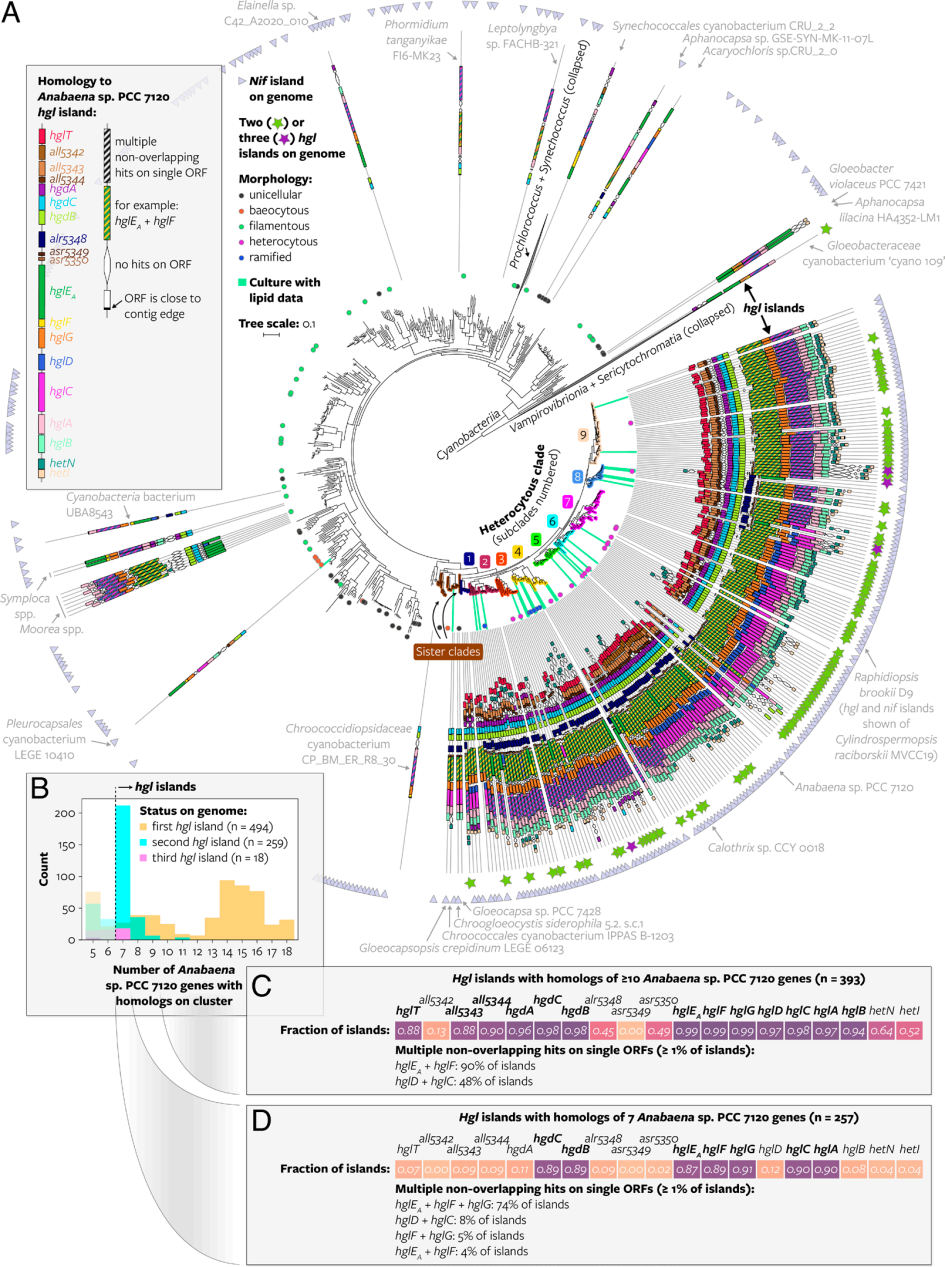
*Hgl* islands within *Cyanobacteriia*. (*A*) Maximum likelihood cyanobacterial phylogeny created using a concatenated alignment of 24 core vertically transferred genes (39) of 1,260 genomes representing species groups (2,758 genomes of cultured and uncultured cyanobacteria clustered at ≥95% average nucleotide identity). The tree is rooted between *Cyanobacteriia* and the nonphotosynthetic *Vampirovibrionia* and *Sericytochromatia* (40, 41). Morphology of specific genomes is indicated based on (36): unicellular (gray); baeocytous (orange), strains capable of forming internal small cells (or endospores) by multiple fissions in parent cells (42); filamentous (green), chain of cells (or trichome) without an investing sheath which grows by intercalary cell division (42, 43); heterocytous (purple) and ramified (blue) are filamentous strains that when grown in the absence of combined nitrogen contain heterocytes in their trichome and that divide in one or in more than one plane, respectively (43). HG lipid data indicated by green shading connecting the tree leaves to the *hgl* island were obtained in this study and in previous studies (*SI Appendix,* Table S1). The *hgl* and *nif* islands of one selected genome per branch are drawn on the tree (*SI Appendix*, *Methods*). The composition of the most extended *hgl* island in terms of number of homologs of the 19 *Anabaena* sp. PCC 7120 *hgl* island genes found on each genome’s island is shown. For size reference, the length of the coding region of *hglE_A_* of *Anabaena* sp. PCC 7120 is 4,626 base pairs. Genomes that contain more than one *hgl* island are indicated with stars, the composition of their additional islands is not shown. The “heterocytous clade” is defined based on the ubiquitous presence of both *hgl* and *nif* islands and contains genomes with unknown morphology. Monophyletic heterocytous subclades were manually selected. The “sister clades” contain the most closely related genomes with known unicellular or baeocytous morphology to the heterocytous clade. The scale bar represents the mean number of substitutions per site. ORF, open reading frame; contig, contiguous sequence. A tree with all leaves labeled and with ultrafast bootstrap approximation values can be found in *SI Appendix,* Fig. S5. Source files in Dataset S4. (*B*) Size distribution of all identified gene clusters. Gene clusters are grouped according to their status in the genome, where the “first” cluster (orange) is the most extended island in terms of number of *Anabaena* sp. PCC 7120 genes with homologs on the island, and the “second” cluster (cyan) the second-most extended island. Sample sizes in the legend are only for *hgl* islands (here defined as genomic clusters containing homologs of ≥7 *Anabaena* sp. PCC 7120 genes). (*C*) Frequency of homologs of HG biosynthesis genes on *hgl* islands containing homologs of ≥10 genes, and (*D*) containing homologs of seven genes. Color-coding in panels (*C* and *D*) is for legibility only and reflects the numbers within the cells, with darker colors representing a higher fraction.

We investigated the presence of *hgl* islands in the context of the phylogeny of the *Cyanobacteriia* based on a concatenation of core vertically transferred genes (Fig. 2*A*, *SI Appendix,* Fig. S5, and Dataset S4). This revealed a monophyletic group that includes cyanobacteria known to produce heterocytes. Although the morphology of many of the 479 cyanobacteria in this clade is unknown, 367 (77%) contain an *hgl* island composed of homologs of ≥10 of the queried genes (Fig. 1*C*), and almost all can fix nitrogen based on the genomic presence of a *nif* island (Fig. 2*A*). This confirms previous suggestions of the occurrence of a single heterocytous clade within the *Cyanobacteriia* (25, 36).

The gene composition of *hgl* islands with homologs of ≥10 genes is largely conserved, with homologs of 13 out of the 19 queried genes present on ≥88% of the islands (Fig. 2 *B* and *C* and *SI Appendix,* Fig. S6 *A* and *B*) and their relative position fixed within the island (*SI Appendix,* Fig. S7). This indicates that evolutionary conservation of genes and their location within the *hgl* island are associated with the preservation of their function in heterocyte formation. Queried genes that are not conserved may be nonessential for HG biosynthesis, be essential but present somewhere else on the genome (e.g., *hetI*, see *SI Appendix,* Fig. S8), or their catalytic function may have been replaced by unrelated genes (*SI Appendix*). The identification of 13 genes that are evolutionary conserved on *hgl* islands is in good agreement with previous mutagenesis studies showing that 12 of the genes are essential in *Anabaena* sp. PCC 7120 for proper HG biosynthesis and deposition (*hgdACB*, *hglTE_A_FGDCAB*, and *all5343*; *SI Appendix*, Fig. S6*B* and Table S4) (14, 44–46).

Remarkably, the genomes of 35 out of the 2,279 cyanobacteria that are not part of the heterocytous clade—and are thus not expected to make heterocytes—also encode an *hgl* island as defined here (i.e., a genomic cluster of homologs of at least seven of the queried HG biosynthesis genes) (Fig. 2*A* and *SI Appendix*, Table S7). They include unicellular cyanobacteria like *Gloeocapsa* sp. PCC 7428, part of a closely related sister clade of heterocytous cyanobacteria in our phylogeny (Fig. 2*A*), and *Gloeobacter violaceus* PCC 7421 (14), a distantly related cyanobacterium found at the base of the *Cyanobacteriia* (Fig. 2*A*), which has a uniquely simple life cycle and cellular composition lacking thylakoids (47, 48). Some of the nonheterocytous cyanobacteria possessing an *hgl* island do not encode a *nif* island (Fig. 2*A*) and are thus likely nondiazotrophic. Most of the *hgl* islands of nonheterocytous cyanobacteria have a more variable gene composition than the *hgl* islands of heterocytous cyanobacteria, with occasional gene duplications, fusions, rearrangements, and absences, and all lack homologs of the gene *hglT* (Fig. 2*A*) encoding the glycosyltransferase (GT) that attaches the glucose headgroup to the AG in the last step of HG biosynthesis (*SI Appendix,* Fig. S1). Hence, although these cyanobacteria possess clusters of homologs of HG biosynthesis genes, their divergent gene composition and phylogenetic position outside the heterocytous clade suggests enigmatic biosynthetic products and a function unrelated to heterocyte formation.

Our genomic screening also revealed that 259 of the 494 *Cyanobacteriia* that have an *hgl* island possess one or more additional *hgl* islands (Fig. 2 *A* and *B*). Many of the cyanobacteria with multiple islands encode a smaller island with a highly conserved gene composition, containing homologs of only seven HG biosynthesis genes (*hgdCB* and *hglE_A_FGCA*) encoded by five open reading frames (ORFs) (Fig. 2*D* and *SI Appendix,* Fig. S6*C*), thus differing from the more extended *hgl* islands with homologs of ≥10 genes discussed above (cf. Fig. 2*C* and *SI Appendix,* Fig. S6*B*). Additional *hgl* islands have previously been reported for four heterocytous cyanobacteria (49); however, our data show that they occur widespread within heterocytous cyanobacteria. Because its gene composition is highly conserved and more limited in size compared to the queried island, it may encode biosynthetic products other than HGs. Hereafter, we refer to these smaller islands within heterocytous cyanobacteria as “*hgl*-like” islands. *Hgl*-like islands are absent in some akinete-forming heterocytous cyanobacteria like *Anabaena variabilis* ATCC 29413, and present as the only island in a few cyanobacteria within the heterocytous clade which have lost the ability to form heterocytes like *Raphidiopsis brookii* D9 (27, 50), suggesting that the biosynthetic products of *hgl*-like islands are not involved in akinete nor heterocyte formation (*SI Appendix*).

Hence, our results demonstrate that HG biosynthesis genes are conserved within a monophyletic heterocytous clade in *Cyanobacteriia* as a cluster resembling the *Anabaena* sp. PCC 7120 *hgl* island. However, several distantly related nonheterocytous cyanobacteria also encode *hgl* islands with a more divergent gene composition, and various cyanobacteria encode one or more additional islands which include “*hgl*-like” islands in heterocytous cyanobacteria. The identified *hgl* islands (including the subset of *hgl*-like islands) provide genomic evidence to elucidate the origin of HGs in *Cyanobacteriia*.

### Heterocytous Cyanobacteria Produce a Wide Diversity of HGs.

**Fig. 3.**
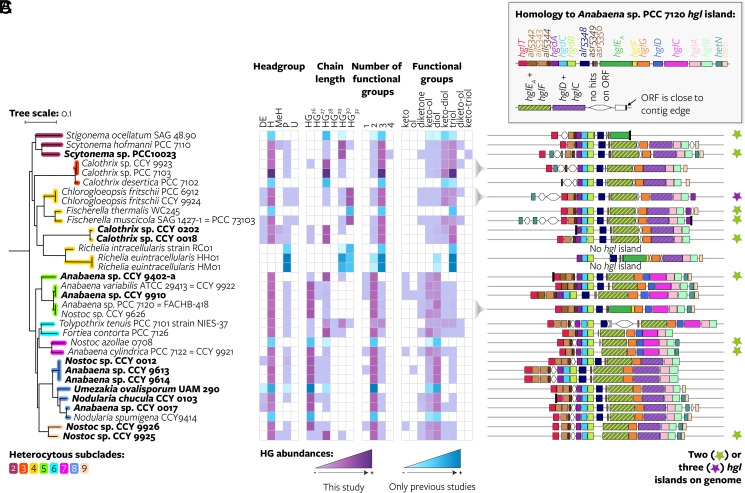
Distribution of 49 HGs grouped according to their structure’s characteristics throughout heterocytous *Cyanobacteriia*. (*A*) Maximum likelihood cyanobacterial phylogeny based on 24 core vertically transferred genes including genomes with a known HG lipid profile (pruned from Fig. 2*A*). Genomes sequenced in this study are shown in bold. The scale bar represents the mean number of substitutions per site. (*B*) Heatmap of HG relative abundances grouped according to the headgroup (H, hexose; DeH, deoxyhexose; MeH, methyl-hexose; P, pentose; U, unknown), chain length, number, and type of functional groups of each HG. The same figure with all individual HGs is shown in *SI Appendix,* Fig. S9. Relative abundances of each HG characteristic are calculated in relation to the sum of all HGs produced by each strain. HG abundances obtained from either this study or previous studies are shown per row as purple or blue heatmaps, respectively. When data from both literature and this study were available only the data collected in this study are shown in the heatmap. Data originally represented in the literature as symbols were converted to percentages as follows: “+++,” 90%; “++,” 40%, “+,” 15%; “tr.,” 1%. Data used to generate this heatmap are found in *SI Appendix,* Table S10. (*C*) Schematic representation of the genes present on the most extended *hgl* island on the genome in terms of number of *Anabaena* sp. PCC 7120 genes with homologs on the island. Stars indicate the presence of two (green) or three (purple) *hgl* islands on the genome; these additional islands are not drawn. ORF, open reading frame; contig, contiguous sequence.

To characterize the structural diversity of HGs produced by heterocytous cyanobacteria, we carried out the largest HG screen to date using ultrahigh-performance liquid chromatography coupled with multistage high-resolution mass spectrometry (UHPLC-HRMS*^n^*). We analyzed the lipid extracts of 24 cyanobacterial cultures evenly sampled throughout the heterocytous phylogeny (Fig. 2*A*). All cultures were grown under nitrogen-limiting conditions, which triggered the production of HGs, comprising 3 to 18% of their total lipids (Fig. 3, *SI Appendix,* Fig. S9 and Tables S1 and S10, and Dataset S5). We screened for 88 theoretically possible HG structures (with different variations of chain lengths, headgroups, and functional groups on their alkyl chain; *SI Appendix*, Tables S8–S10), 19 of which were previously reported in heterocytous cyanobacteria (8, 10, 11, 20, 23, 24). This revealed the presence of 30 previously unreported HGs, increasing the number of described HGs to 49 (*SI Appendix*, Figs. S10–S13 and Table S9). Previously, only HGs with an even number of carbon atoms in their alkyl chain had been described (9, 12), yet we also detected odd-numbered HGs (hexose HG_27_ diol and hexose HG_29_ keto-ol) albeit in small amounts. Although most HGs described thus far contain a hexose headgroup, we identified HGs with various other headgroups, including a still undetermined C_6_ sugar. In addition, our broad screening reveals unique chain length and headgroup combinations. The functional groups on the alkyl chain of the HGs are usually diols, keto-ols, keto-diols, or triols, but we also observe atypical combinations of keto and alcohol groups such as diketo-ols, monofunctional HGs, and an HG with four functional groups. The identification of these lipids highlights the wide diversity of HGs that are produced by heterocytous cyanobacteria today and opens a broad avenue in the study of the role of HGs in heterocyte impermeabilization and its link to nitrogen fixation.

### HG Structure Is Mostly Independent of *hgl* Island Gene Composition.

We next analyzed the distribution of HGs grouped according to their structure’s characteristics—headgroup, alkyl chain length, and type and number of functional groups on their alkyl chain (Fig. 3). Most cyanobacteria produce predominant HGs with a single combination of headgroup and chain length and with two or three functional groups, and a variety of other HGs in low abundance (Fig. 3*B*). This confirms previous observations of heterocytous cyanobacteria harboring two predominant HGs that are structurally identical except for differences in the number and stereochemistry of keto and alcohol groups they carry (8). For example, the two most abundant HGs produced by *Chlorogloeopsis fritschii* PCC 6912 are hexose HG_32_ keto-diol and hexose HG_32_ triol [this study and (8)]. However, others such as *Tolypothrix tenuis* PCC 7101 produce multiple HGs with different headgroups, chain lengths, and number of functional groups in high abundance [Fig. 3*B* and *SI Appendix*; this study and (8)].

No direct relationship was found between the type of HGs cyanobacteria produced and the composition of their (most extended) *hgl* island (Fig. 3*C* and *SI Appendix,* Fig. S9). This shows that differences in the composition of the *hgl* islands (e.g., the presence or absence of homologs of *all5342*, *alr5348*, *asr5349*, *asr5350*, *hetN*, or *hetI*, gene fusions or fissions, or the presence of ORFs that are not homologous to the queried proteins) do not drive the production of predominant HGs. Moreover, we did not find specific HGs associated with the additional presence of an *hgl*-like island on the genome, further suggesting that the more compact *hgl*-like islands are not involved in HG biosynthesis. The observed variation between cyanobacteria in the HG structures produced (Fig. 3*B*) is thus likely not caused by differences in the composition of the *hgl* islands, but due to small differences in gene sequence that result in different enzymatic activities of the proteins encoded by the *hgl* island (see section “*HG Structure Evolved Convergently*”)—as for example has also been observed in long-chain fatty acid biosynthesis catalyzed by related PKSs (51), or by differences in available precursors, cell physiology, environmental conditions, or a combination of these factors. An exception is *hglT*, the glycosyltransferase (GT) that attaches the glucose headgroup to the AG (*SI Appendix,* Fig. S1), which is absent from the genomes of three *Richelia* species that form symbiotic relationships with marine diatoms (*SI Appendix*, Figs. S9 and S14). *Richelia euintracellularis* HM01 and *Richelia intracellularis* RC01 both exclusively produce HGs with a pentose instead of the common hexose headgroup (Fig. 3*B*), which have been suggested as biomarkers for diatom-diazotroph associations (10, 21), and the HG profile of *Richelia rhizosoleniae* SC01 is unknown. Thus, the production of pentose HGs may be directly related to the absence of *hglT* from the *hgl* island, and its possible replacement by an alternative GT with pentose specificity. We thoroughly searched for this alternative GT on the genome of *R. euintracellularis* HH01 and identified four candidate genes based on sequence homology to *hglT*, and functional annotation as GT together with gene localization close to the *hgl* island (see *SI Appendix* for details). However, heterologous expression of the four candidate genes in an *hglT*-deficient *Anabaena* sp. PCC 7120 strain did not result in production of pentose HGs (*SI Appendix*, Figs. S15–S17 and Tables S11–S15 and Dataset S6).

### HG Structure Evolved Convergently.

**Fig. 4.**
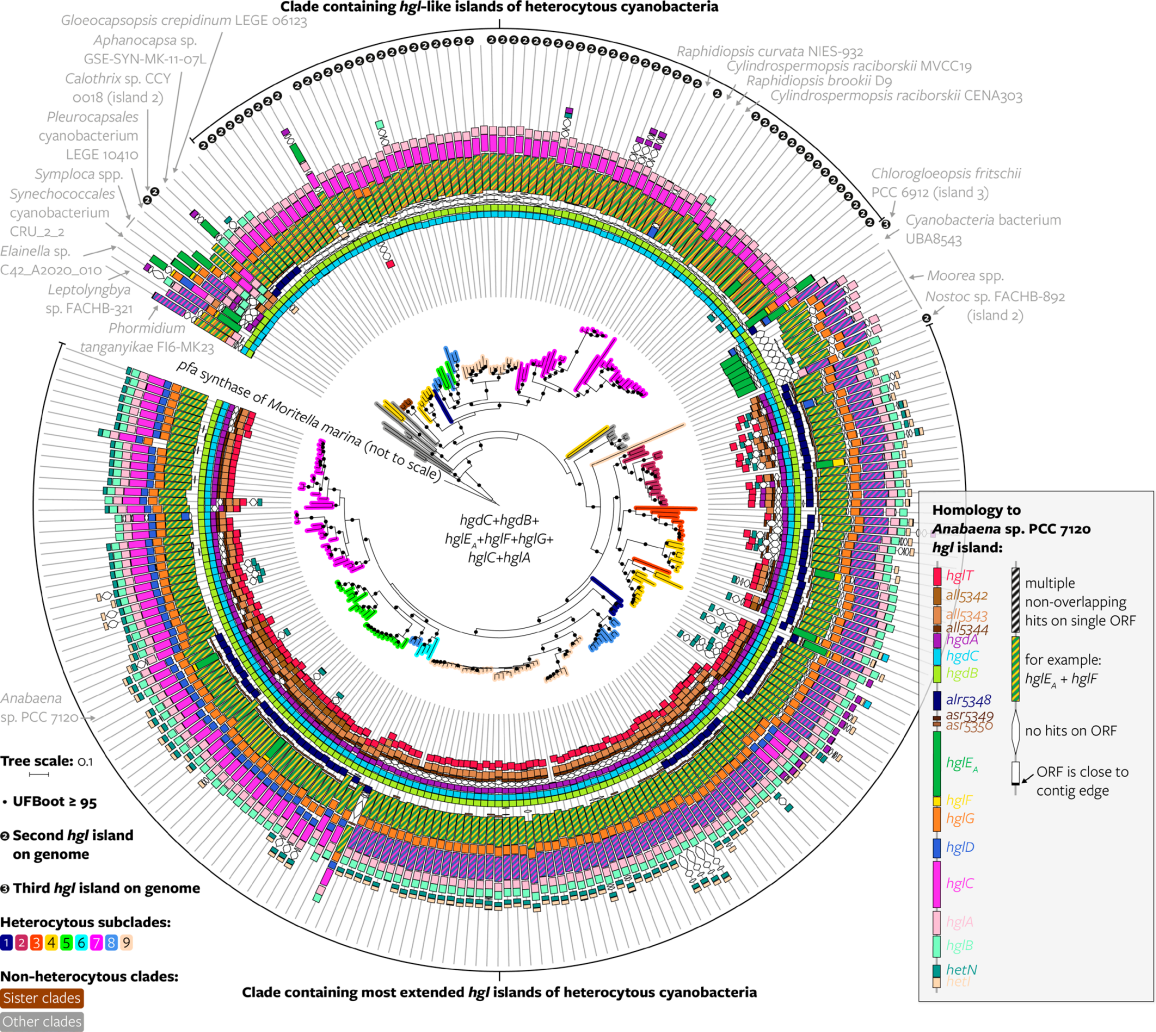
Evolutionary history of *hgl* islands within *Cyanobacteriia*. Maximum likelihood phylogeny of the *hgl* island created using a concatenated alignment of homologous sequences of seven HG biosynthesis genes (*hgdCB* and *hglE_A_FGCA*) that are often present on *hgl* islands. Heterocytous and nonheterocytous subclades represent the position of the genome in the manually defined monophyletic clades of Fig. 2*A* based on a concatenation of 24 core vertically transferred genes. Genomes that are not part of a manually defined clade in Fig. 2*A* fall within the “other clades” category in this figure. The same species might appear more than once in the tree depending on how many islands it possesses; “first,” “second,” and “third” *hgl* islands are based on the presence of other islands on the genome, where the “first” *hgl* island (not marked) is the most extended island in terms of number of *Anabaena* sp. PCC 7120 genes with homologs on the island, the second *hgl* island (marked with a 2) the second-most extended island, and the third island (marked with 3) the third-most extended island. The *hgl*-like islands of *Raphidiopsis curvata* NIES-932, *Cylindrospermopsis raciborskii* CENA303, and *R. brookii* D9 are indicated because they are nondiazotrophic cyanobacteria from within the heterocytous clade. The tree is rooted in between the cyanobacterial *hgl* islands and the *pfa* synthase of the gammaproteobacterium *M. marina* ATCC 15381—this *pfa* cluster does not contain *hgdCB* homologs, and their alignments were filled with gaps. The root is artificially shortened for legibility. The scale bar represents the mean number of substitutions per site. ORF, open reading frame; contig, contiguous sequence; UFBoot, ultrafast bootstrap approximation. A tree with all leaves labeled and with an unshortened root can be found in *SI Appendix,* Fig. S19, and an unrooted version in *SI Appendix,* Fig. S20. Source files in Dataset S7.

**Fig. 5.**
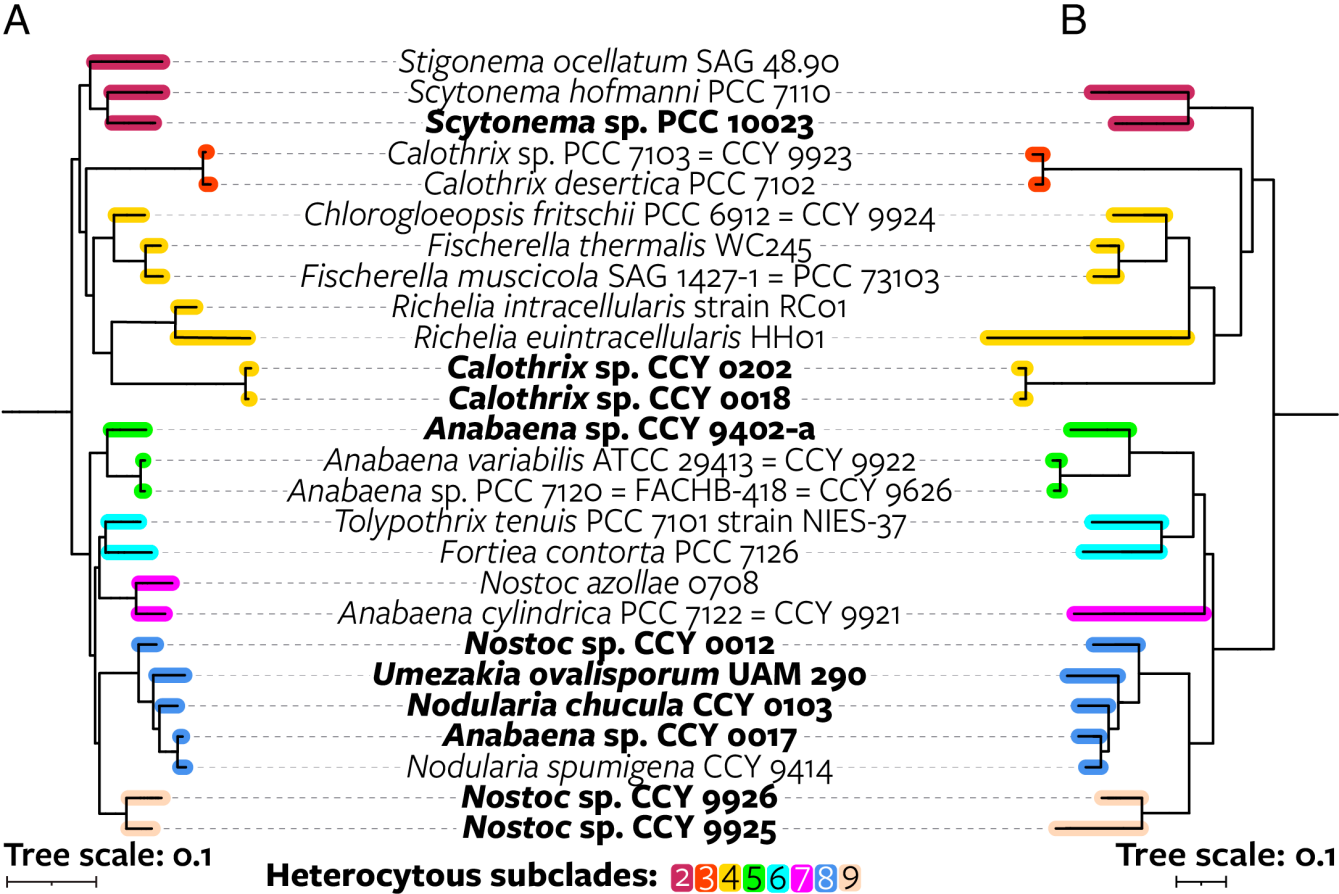
Comparison between maximum likelihood phylogenies based on 24 core vertically transferred genes and seven *hgl* island genes of genomes with a known HG profile. (*A*) Cyanobacterial phylogeny based on 24 core vertically transferred genes. (*B*) Phylogeny of the *hgl* island based on homologous sequences of seven HG biosynthesis genes (*hgdCB* and *hglE_A_FGCA*) that are often present on *hgl* islands. For each cyanobacterium, only the most extended *hgl* island in terms of number of *Anabaena* sp. PCC 7120 genes with homologs on the island is shown. These *hgl* islands closely resemble the *hgl* island of *Anabaena* sp. PCC 7120 in terms of gene composition as opposed to the more compact *hgl*-like islands. The tree is rooted outside the depicted clade. Genomes sequenced in this study are shown in bold. No genomes from subclade 1 are present in the figure because this subclade does not contain cultured representatives with lipid data. Scale bars represent the mean number of substitutions per site.

Next, we assessed whether the variation in HG production between cyanobacteria can be explained by evolutionary relatedness, by evaluating the observed HG structural diversity in the context of the core gene phylogeny (Fig. 3 and *SI Appendix,* Fig. S9), which represents the evolutionary history of the vertically transferred part of the genome. Since clusters of HG biosynthesis genes can be horizontally transferred—as suggested by their identification on a plasmid (*SI Appendix,* Fig. S2)—and may thus have a different evolutionary history, we constructed phylogenies of seven genes that are shared among most *hgl* and *hgl*-like islands (*hgdCB* and *hglE_A_FGCA*) to reconstruct the evolutionary history of the islands. As the seven phylogenies (*SI Appendix,* Fig. S18) had similar tree topologies—showing that the genes have congruent evolutionary histories—we also constructed a phylogeny based on the concatenated alignment of the seven genes to increase the phylogenetic signal (Fig. 4, *SI Appendix*, Figs. S19–S22, and Dataset S7). In this phylogeny of the seven concatenated HG biosynthesis genes, the most extended *hgl* islands of heterocytous cyanobacteria cluster closely together and separately from the *hgl* islands of nonheterocytous cyanobacteria and *hgl*-like islands within heterocytous cyanobacteria (Fig. 4). Additionally, the most extended *hgl* islands of heterocytous cyanobacteria have a tree topology largely similar to that of the core vertically transferred genes (Fig. 5), suggesting a shared evolutionary history of the core genes and *hgl* islands within heterocytous cyanobacteria (*SI Appendix*). Because of this shared history, similar HG distribution patterns can be observed across both phylogenies (Fig. 3 versus *SI Appendix,* Fig. S23).

Specific HGs are common within heterocytous subclades, such as the above discussed pentose HGs of two *Richelia* species. Strains from subclades 2 to 4 mainly produce dominant HGs with three functional groups and even-numbered alkyl chains with ≥28 carbon atoms. Strains from subclades 5 to 9 produce primarily HG_26_’s with two functional groups. However, few HGs are unique to a subclade, and most HGs—especially those that are dominant in one of the cultures—are found throughout the phylogeny even if in low abundance (Fig. 3 and *SI Appendix*, Figs. S9 and S23). For example, the pentose headgroup, which was previously detected in only a few strains, is present in most cultures in relatively low abundance, although possibly in pentopyranose form (*SI Appendix*). Exceptions are HGs with a single functional group and odd-chained HGs, which are both restricted to subclades 5 to 9, and pentose HG_30_ keto-triol—only found in *Scytonema* sp. PCC 10023 (*SI Appendix,* Fig. S9). In addition, although cultures from closely related strains usually produce similar HGs, this is not always the case. For example, *Nostoc* sp. CCY 9925 and *Nostoc* sp. CCY 9926 synthesize primarily hexose HG_28_ and hexose HG_26_, respectively.

Thus, the same HGs can be found in distantly related strains and closely related strains can differ in their abundant HGs, hence challenging the common use of HGs as taxonomic biomarkers, for example for members of the *Nostocaceae* and *Aphanizomenonaceae* families and for the genus *Fortieaceae* (*SI Appendix*, Table S16). These findings suggest that the biosynthetic process leading to HGs is flexible and can evolve convergently, i.e., independent evolution of the same HG structures in different taxonomic groups (*SI Appendix*), possibly reflecting similar environmental pressures, leading to a chemically diverse pool of HGs to accommodate them.

### *Hgl* Islands Predate Heterocyte Formation.

The identification of *hgl* islands in nonheterocytous cyanobacteria and of *hgl*-like islands allowed for a reconstruction of the acquisition of HG biosynthesis within the *Cyanobacteriia* (*SI Appendix,* Fig. S21). As discussed above, the most extended *hgl* islands of heterocytous cyanobacteria cluster together in the phylogeny based on seven HG biosynthesis genes, as do their *hgl*-like islands (Fig. 4), and the tree topology of the most extended *hgl* islands of heterocytous cyanobacteria in this phylogeny resembles that of the core genes (Fig. 5). A duplication that gave rise to both island types in heterocytous cyanobacteria thus predated the Last Heterocytous Cyanobacterial Common Ancestor (LHeCCA) and potentially took place further back in time before the last common ancestor of the heterocytous cyanobacteria and their sister clades or even before their last common ancestor with the genus *Moorea* and *Cyanobacteria* bacterium UBA8543 (*SI Appendix,* Fig. S21*A*). Phylogenetic placement of the *hgl* islands of nonheterocytous cyanobacteria—as deeper branching sister clades and in between the two heterocytous phylogenetic clusters (Fig. 4 and *SI Appendix,* Fig. S21*A*)—suggests they were not acquired via recent horizontal transfer from heterocytous cyanobacteria and supports the presence of *hgl* islands further back in time.

To further elucidate the origin of *hgl* islands in *Cyanobacteriia*, we constructed an extended phylogeny of homologs of the longest gene in the queried *hgl* island, *hglE_A_*, which included homologs from islands that did not encode all seven HG biosynthesis genes used to construct the concatenated HG biosynthesis genes tree (like the *hgl* island of *G. violaceus* PCC 7421), and homologs that were not present on an *hgl* island by our definition. In this phylogeny (*SI Appendix*, Figs. S24 and S25 and Dataset S8), *hglE_A_* homologs present in the *hgl* islands of basally branching *Cyanobacteriia*—*G. violaceus* PCC 7421, *Aphanocapsa lilacina* HA4352-LM1, and *Gloeobacteraceae* cyanobacterium “cyano 109”—also represent the oldest branching lineages. This may suggest that their *hgl* islands are ancestral remnants rather than more recent transfer from around the time of LHeCCA, supporting the presence of homologs of HG biosynthesis genes with yet unknown biosynthetic product in (or very close in time to) the ancestor of all *Cyanobacteriia*. Both the phylogenies of *hglE_A_* homologs and of the seven concatenated HG biosynthesis genes imply that a complex evolutionary history of loss, duplication, and horizontal transfer shaped the distribution of *hgl* islands in contemporary cyanobacterial genomes (*SI Appendix*).

Hence, the *hgl* islands in specific nonheterocytous cyanobacteria likely represent a phylogenetic lineage of the island from before the emergence of the heterocyte. Their diverse gene compositions suggest that this ancestral island already contained many homologs of the contemporary HG biosynthesis genes—except for possibly *hglT* which is absent from all nonheterocytous *hgl* islands—and reflect subsequent differential loss of individual genes.

### Potential Origin of HGs from Ancient 1,3-Diols.

**Fig. 6.**
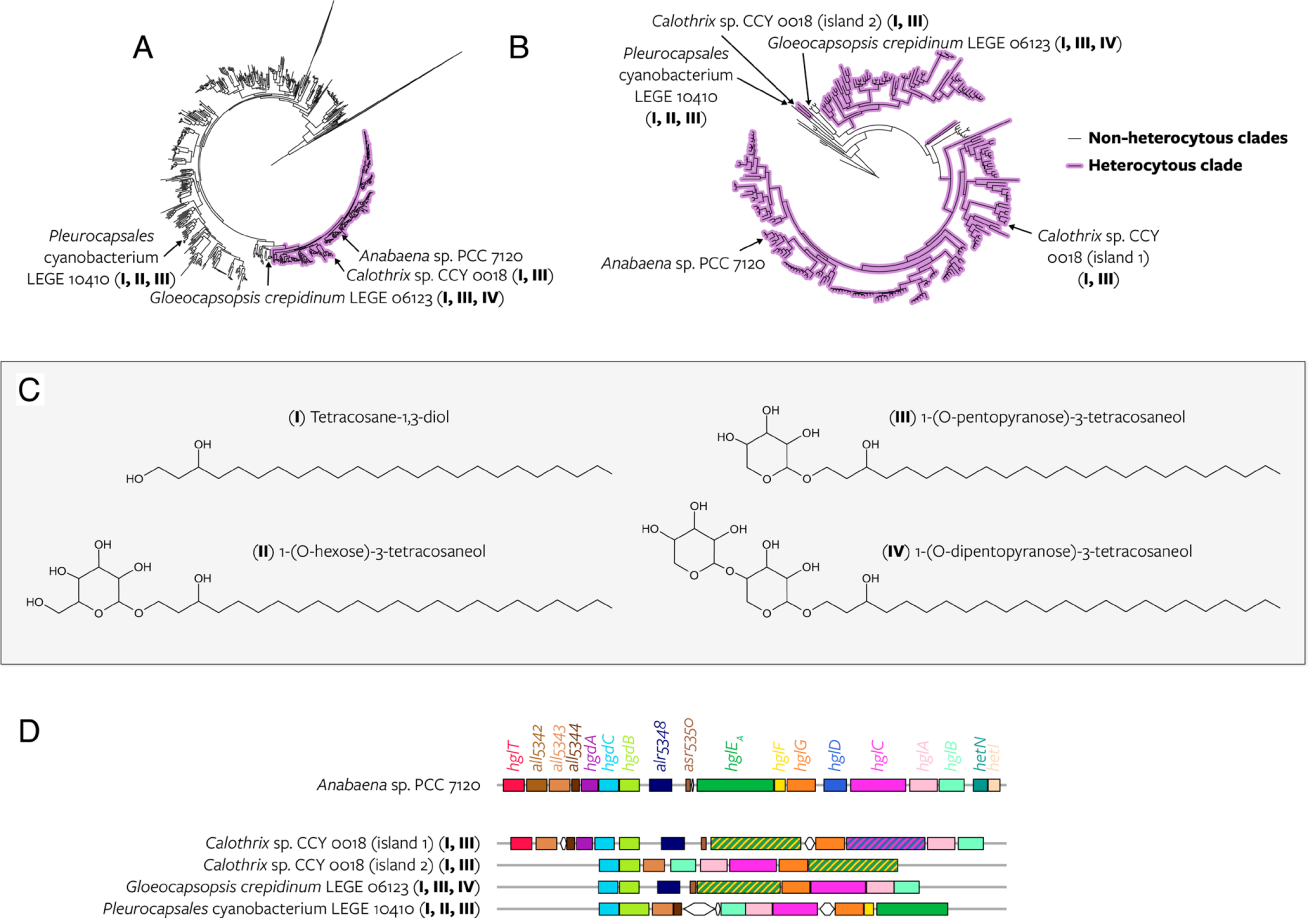
Two nonheterocytous and one heterocytous cyanobacteria produce HG analogs. (*A*) Position of cyanobacteria that produce HG analogs in the phylogeny based on the concatenated alignment of 24 core vertically transferred genes (Fig. 2*A*). The HG analogs that they produce are depicted in the panel below and indicated with Roman numerals I-IV throughout the figure. *Pleurocapsales* cyanobacterium LEGE 10410 and *G. crepidinum* LEGE 06123 are the only nonheterocytous cyanobacteria whose lipids were analyzed. *Anabaena* sp. PCC 7120 does not produce HG analogs but is indicated for reference. (*B*) Position of the *hgl* islands of the same cyanobacteria in the concatenated alignment of homologous sequences of seven HG biosynthesis genes (Fig. 4). Both *hgl* islands of *Calothrix* sp. CCY 0018 are indicated. (*C*) Tentative structure of HG analogs. (*D*) *Hgl* islands encoded by the genomes of *Anabaena* sp. PCC 7120 and the three cyanobacteria that produce HG analogs.

To elucidate the evolutionary origin of HG biosynthesis, we selected two cultured strains from outside the heterocytous clade that contain an *hgl* island and are capable of nitrogen fixation—*Pleurocapsales* cyanobacterium LEGE 10410 and *Gloeocapsopsis crepidinum* LEGE 06123 (Fig. 6 *A* and *B*)—and exposed them to heterocyte- and akinete-inducing stresses, namely nitrogen deficiency and aging (31, 52). Microscopic analysis revealed that upon division, daughter cells of both strains remain attached to the parent cell and are surrounded by a polysaccharide layer, thus appearing as aggregates (*SI Appendix,* Fig. S26), under all growth conditions. Because their *hgl* islands lack *hglT* homologs and contain potential adenosine triphosphate (ATP)-binding cassette (ABC) transporters expressed by the *hgdBC* homologs, we hypothesized they can be involved in the biosynthesis and export of AGs.

Although no C_26_–C_32_ HGs nor derived AGs were detected, both strains synthesize lipids that structurally resemble canonical HGs and AGs, but with a shorter C_24_ alkyl chain and lacking a characteristic functional group at the ω-1 position (*SI Appendix*, Figs. S27–S30). While some are observed in diol form (tetracosane-1,3-diol), thus resembling AGs, others consist of this same C_24_ alkyl chain compound bound to different sugars [1-(O-hexose)-3-tetracosaneol, 1-(O-pentopyranose)-3-tetracosaneol, and 1-(O-dipentopyranose)-3-tetracosaneol], thus resembling HGs (hereafter “HG analogs”) (Fig. 6*C* and *SI Appendix*, Tables S10, S17, and S18). We speculate that these lipids are produced by the enzymes encoded by the *hgl* islands of the nonheterocytous strains (Fig. 6*D* and *SI Appendix*). Analysis of the lipid extracts of the heterocytous cultures revealed that *Calothrix* sp. CCY 0018, which encodes an additional *hgl* island closely related to the *hgl* island of *Pleurocapsales* cyanobacterium LEGE 10410 (Fig. 6*B* and *SI Appendix,* Fig. S21*A*), also produces HG analogs (*SI Appendix,* Fig. S31 and Table S10), providing further evidence for the biosynthesis of these lipids by an ancient phylogenetic lineage of the *hgl* island (*SI Appendix*).

As the ancestor of these islands was likely present in *Cyanobacteriia* before the emergence of the heterocyte (see above), and the three islands are distantly related yet may produce similar products (*SI Appendix,* Fig. S21), we propose that ancestors of LHeCCA were already capable of synthesizing and exporting C_24_ alkyl chain lipids resembling HGs and AGs for still unknown purposes (*SI Appendix*). Because these lipids were detected under all growth conditions including in nitrogen-rich media (therefore not promoting nitrogen fixation), and because *hgl* islands are also present in nondiazotrophic cyanobacteria outside the heterocytous clade (Fig. 2*A* and *SI Appendix*, Table S7) the HG analogs produced by contemporary cyanobacteria, and conceivably by extension by the ancestors of LHeCCA, are probably not associated with nitrogen fixation nor with (a process similar to) akinete formation.

In this scenario, the modified biosynthetic steps encoded by one of the copies of the *hgl* island of LHeCCA would have allowed for the emergence of HGs from HG analogs by chain length extension and the addition of keto, alcohol, and methyl groups (*SI Appendix*) and facilitated their unique role as nitrogenase protectors in an increasingly oxygenated atmosphere. The absence of contemporary HGs with a C_24_ alkyl chain may reflect selection for longer chain length potentially providing a higher oxygen impermeability to the heterocyte membrane throughout the course of the evolution of heterocytous cyanobacteria.

Even though the last common ancestor of the *hgl* islands of *Pleurocapsales cyanobacterium* LEGE 10410, *G. crepidinum* LEGE 06123, and *Calothrix* sp. CCY 0018 predated LHeCCA, it is not the last common ancestor of all nonheterocytous *hgl* islands (*SI Appendix,* Fig. S21). Therefore, it remains unknown whether the first cyanobacterial *hgl* island—potentially already present in the last common ancestor of all *Cyanobacteriia* (see above)—could already make the HG analogs. Given the close relationship of cyanobacterial *hgl* islands to *pfa* synthase in other taxonomic groups, the first cyanobacterial *hgl* island may also have been involved in PUFA biosynthesis. PUFAs have been linked to antioxidative roles (53–56) and may be beneficial to facultative and strict anaerobic bacteria (38). Comprehensive high-resolution characterization of the lipids produced by basally branching *Cyanobacteriia* with an *hgl* island, like *G. violaceus* PCC 7421, and elucidation of the enzymatic potential encoded by their genomes, will further our understanding of the evolution of HG analogs in *Cyanobacteriia*.

## Conclusions

The broad screening of HG composition in cultures revealed a large structural diversity of HGs within heterocytous cyanobacteria—nearly tripling known HG structural diversity. Our findings also highlight the reduced taxonomic value of HGs, which complicates their use to identify their producers in present and past environments.

Our results support the origin of HGs from ancient HG analogs that were not associated with nitrogen fixation—remnants of which may still be found in specific nonheterocytous and heterocytous cyanobacteria today. Genomic clusters of HG biosynthesis genes were probably present in *Cyanobacteriia* before the rise of the heterocyte. Neofunctionalization of this existing genomic machinery for the production and export of 1,3-diols and related sugar-bound lipids allowed for the biosynthesis of HGs and the formation of an oxygen-impermeable membrane layer to further protect nitrogenase, the key enzyme for nitrogen fixation. These findings suggest the usefulness of AG, HGs, and HG analogs to trace the evolution of multicellularity and division of labor in cyanobacteria, which remains challenging to pinpoint due to scarcity of fossil evidence. Future studies will need to evaluate the preservation potential and diagenetic transformations of these molecules to fully conclude their use as fossils for molecular clock calculations of key events in Earth’s history and microbial evolution.

## Materials and Methods

More detailed *Materials and Methods* are provided in *SI Appendix*.

### Bacterial Strains and Growth Conditions.

Most cyanobacterial biomass from heterocytous strains was obtained from frozen (−80 °C) culture pellets stored in the former Culture Collection Yerseke (CCY) and from starter cultures provided by the Pasteur Culture Collection (PCC) in the appropriate growth media (*SI Appendix*, Tables S1 and S2). The exceptions were *Umezakia ovalisporum* strains UAM290 (57) and UAM292 (formerly known as *Chrysosporum ovalisporum* or *Aphanizomenon ovalisporum*), kindly provided by Samuel Cirés, and *Anabaena* sp. PCC 7120 *wild type* (*wt*) and ∆*hglT* (16), kindly provided by Koichiro Awai. Nonheterocytous strains were kindly provided by the Blue Biotechnology and Ecotoxicology Culture Collection (LEGE-CC) (58). For further details regarding the growth conditions see *SI Appendix*.

### Genome Sequencing.

Genomic DNA was extracted from the 14 strains described in *SI Appendix*, Table S3 using the DNeasy PowerSoil kit (Qiagen, Hilden, Germany) with minor modifications, sequenced using the Illumina NovaSeq 6000 sequencing platform and assembled using BiosyntheticSPAdes (v3.14.1) (59) as described in detail in *SI Appendix*.

### Query of HG Biosynthesis and *nif* Genes in Cyanobacterial Genomes and Island Definition.

A total of 3,657 genomes including plasmids that had a taxonomic assignment as “phylum *Cyanobacteria*” were selected from the PATRIC genome database (60) [currently part of the BV-BRC database (32)] as described in detail in *SI Appendix*. Predicted proteins were queried for the 19 protein sequences that are encoded by the *hgl* island of *Anabaena* sp. PCC 7120 and for 14 *nif* protein sequences which are part of a nitrogenase gene cluster in *Anabaena* sp. PCC 7120. ORFs with hits were considered part of a genomic cluster if they were separated by ≤3 ORFs on the contig. We examined genomic clusters containing homologs of at least seven of the queried HG biosynthesis genes—i.e., a genomic cluster of ORFs with hits to at least seven different HG biosynthesis genes irrespective of the number of ORFs on which they were encoded or the copy number of the gene on the cluster—as “*hgl* islands.” “*Nif* islands” were defined similarly but with a minimum of five different *nif* homologs. Further details are provided in *SI Appendix*.

### Cyanobacterial Core Gene Phylogeny.

Species-level groups were identified in a set of 2,777 genomes (selection criteria in *SI Appendix*) with dRep (v3.4.0) (61) using a similarity cutoff of 95% average nucleotide identity (ANI), which has been suggested as species-level boundary (62). The representative genome of each dRep cluster was used for constructing a phylogeny based on a concatenation of 24 core genes that were selected from the 27 Clusters of Orthologous Gene (COG) families that showed evidence of being primarily vertically transferred in ref. 39 (identification and selection criteria in *SI Appendix*). Genes were individually aligned with MAFFT (63), the alignment was trimmed with trimAl (v1.4.rev15) (64), and the aligned sequences were concatenated per genome. The final concatenated alignment contained 1,260 representative genomes (representing 2,758 genomes) and 6,933 amino acids.

A phylogenetic tree was constructed with IQ-TREE (v2.1.2) (65) (further details in *SI Appendix*), and visualized and decorated in Interactive Tree of Life (iTOL) (66). The branches of this phylogeny are thus representative genomes of dRep clusters that may contain multiple genomes. We decorated each branch on the core gene phylogeny with *hgl* and *nif* islands of one selected genome from the dRep cluster, and the genome that was chosen for this visualization was not always the dRep representative genome that was used to construct the core gene phylogeny (see *SI Appendix* for details).

### Phylogenies of Seven *hgl* Island Genes and of a Concatenated Alignment of These Genes.

We constructed seven phylogenies of homologs of seven HG biosynthesis genes—*hgdCB* and *hglE_A_FGCA*. Only homologs from the *hgl* islands that were drawn on the core gene phylogeny were included, and homologs from *hgl* islands that did not contain a hit to all seven genes were excluded. We also constructed a phylogeny based on a concatenation of these homologs of seven HG biosynthesis genes, excluding five heterocytous islands whose placements in the individual gene trees were incongruent, and including the *hgl* islands of four heterocytous cyanobacteria that were not drawn on the core gene phylogeny (see *SI Appendix* for details) and the *pfa* synthase of the gammaproteobacterium *Moritella marina* ATCC 15381 as outgroup which encodes *hglE_A_FGCA* homologs but no homologs of *hgdCB*. Further details are provided in *SI Appendix*.

### Query of HG Biosynthesis Genes throughout Prokaryotes.

*Hgl* islands were also identified in 225,388 prokaryotic genomes from the PATRIC genome database. Taxonomy of the genomes (encompassing 182 prokaryotic phyla) was based on the “genome_lineage” file on the PATRIC ftp server. The genome of *Beggiatoa* sp. 4572_84 and its contigs were taxonomically reannotated with Bin Annotation Tool (BAT) and Contig Annotation Tool (CAT), respectively, from the CAT pack software suite (v6.0.1) (67). Further details are provided in *SI Appendix*.

### Extended Phylogeny of *hglE_A_* Homologs.

A second *hglE_A_* phylogeny was constructed including a larger set of sequences. We included the *hglE_A_* homologs that were included in the phylogeny of a concatenated alignment of seven HG biosynthesis genes, the *hglE_A_* homologs on *hgl* islands that were not included in this phylogeny but were drawn on the cyanobacterial core gene phylogeny, and all *hglE_A_* hits that had a bit-score ≥1,150 of the genomes that were selected to be drawn on the core gene phylogeny from the dRep cluster (see above). The latter group includes cyanobacterial hits on additional *hgl* islands that do not encode homologs of all seven HG biosynthesis genes—and are thus not drawn on the core gene phylogeny and not included in the phylogeny based on seven HG biosynthesis genes—and cyanobacterial hits that are not present on an *hgl* island. We moreover added noncyanobacterial *hglE_A_* hits that had a bit-score ≥1,150. Further details are provided in *SI Appendix*.

### Plasmid Construction and Transformation.

Prior to further genetic modifications, we confirmed the substitution of *hglT* for the *npt* kanamycin resistance cassette in the Δ*hglT Anabaena* strain via PCR using two different primer sets (*SI Appendix,* Fig. S15 and Table S11). Our results confirmed that *hglT* had been successfully deleted in all genome copies.

The following genes of interest were selected from the *R. intracellularis* HH01 genome (taxon ID 2579778779) and their sequence obtained by using The Integrated Microbial Genomes & Microbiomes system (IMG/M) (68): RINTHH_5560, RINTHH_5570, RINTHH_17770, and RINTHH_20790. Because RINTHH_5560 and RINTHH_5570 are closely located on the genome, a construct containing both genes and their intergenic region (42 bp) (RINTHH_5560_5570) was also included. Additionally, an alternative ORF annotated as a glycosyltransferase by RAST (69) that was highly similar to RINTHH_20790, albeit shorter (39 bp at the 5′) was also selected. All genes were synthesized by Baseclear BV (Leiden, the Netherlands) under the control of P*_glnA_*, a constitutive promoter active in heterocyte and vegetative cells (70). The genes of interest and the streptomycin/spectinomycin resistance cassette *aadA* (71) were inserted in pAM5404 (71) using NEBuilder HiFi DNA Assembly Cloning Kit (New England Biolabs) according to the manufacturer’s instructions, transformed into NEB 5-alpha chemically competent *Escherichia coli* cells and plated on LB agar plates containing spectinomycin and streptomycin. Genetic modification by triparental mating of *Anabaena* sp. PCC 7120 ∆*hglT* was carried out as described in refs. 72 and 73. Further details are provided in *SI Appendix*.

### Lipid Extraction and Analysis.

Extraction of intact polar lipids (IPL) from freeze-dried biomass was carried out using a modified Bligh Dyer (BD) extraction as described in ref. 74. A known amount of deuterated diacylglyceryltrimethylhomoserine (DGTS D-9, Avanti Polar Lipids, USA) dissolved in dichloromethane (DCM): Methanol (MeOH) (1:9, v:v) was added to the extracts as internal standard, filtered through a true regenerated cellulose 4 mm syringe filter (0.4 µM, BGB, USA) and analyzed on an Agilent 1290 Infinity I UHPLC with a thermostatted autoinjector, coupled to a Q Exactive Orbitrap Mass Spectrometer with an Ion Max source and heated electrospray ionization probe (HESI; ThermoFisher Scientific, Waltham, MA) according to ref. 75 (modified from ref. 9). HGs were identified using a targeted approach based on fragmentation of the theoretical HGs and AGs (extrapolated from the fragmentation of closely related HGs) (*SI Appendix*, Table S8).

In order to screen for AG-like components in the two LEGE strains, their BD extracts were also analyzed by gas chromatography-mass spectrometry (GC-MS, Agilent 7990B GC coupled to Agilent 5977A MSD equipped with a fused silica capillary column Agilent CP Sil-5, 25 m × 0.32 mm × 0.12 µm) after methylation and silyilation. In addition, some extracts were desilylated by washing five times in DCM (500 µL) and drying under N_2_, and then resilylated by the method above, but with addition of deuterated N,O-Bis(trimethylsilyl)trifluoroacetamide (5 µL) before reanalysis. Further details are provided in *SI Appendix*.

## Supplementary Material

Appendix 01 (PDF)

Dataset S01 (XLSX)

## Data Availability

Sequencing data and genome assemblies were deposited in the European Nucleotide Archive under project number PRJEB67822 (76). The code used for this manuscript, the identified *hgl* islands, phylogenetic trees raw files, UHPLC-HRMS*^n^* (Orbitrap) and GC-MS datafiles, and plasmid maps can be found in Datasets S1–S8 on Zenodo at https://doi.org/10.5281/zenodo.14019165 (77).
