# Supplementary material for: Emergence and evolution of heterocyte glycolipid biosynthesis enabled specialized nitrogen fixation in cyanobacteria
Source: Proc Natl Acad Sci U S A. 2025 Jan 27;122(5):e2413972122. doi: 10.1073/pnas.2413972122 (PMC11804610; doi:10.1073/pnas.2413972122)
Supplement: Supplementary file 1 — Appendix 01 (PDF) [file pnas.2413972122.sapp.pdf]

Emergence and evolution of heterocyte glycolipid biosynthesis enabled specialized nitrogen fixation in cyanobacteria

Ruth Pérez Gallego*, F. A. Bastiaan von Meijenfeldt*,

Nicole J. Bale, Jaap S. Sinninghe Damsté, and Laura Villanueva

Supplementary Information

Includes

Supplementary Results and Discussion,

Supplementary Materials and Methods,

Supplementary Figs. 1-31,

and 103 Supplementary References.

Supplementary Tables 1-18 are supplied as an additional file,

and Supplementary Data 1-8 can be found on Zenodo at https://doi.org/10.5281/zenodo.14019165.

Supplementary Results and Discussion

Genomic prediction of heterocyte glycolipid (HG) biosynthesis in *Cyanobacteriia*

Homology search of HG biosynthesis genes

The HG gene cluster on the genome of the model cyanobacterium for heterocyte formation *Anabaena* sp. PCC 7120 was initially referred to as an 'expressed island', a group of physically clustered genes encompassing multiple transcription units that were regulated in a coordinated manner (1). However, since it included known '*hgl*' genes, it was later renamed to '*hgl* island' (2). Here, we use the latter name.

The *hgl* island has traits of iterative type I and type II polyketide synthases (PKSs), containing large enzymes consisting of several functional domains (for example ketoacyl synthase, acyl transferase, and acyl carrier protein (ACP) domains on $hglE_A$ (2)) as well as monofunctional enzymes (for example glycosyltransferase *hglT* (3, 4) and ketoacyl synthase *hglD* (2)) (Fig. 1b). Other distantly related PKSs in cyanobacteria are responsible for the production of a wide variety of secondary metabolites with diverse functions, such as cytotoxins (e.g. cylindrospermopsin, lyngbyatoxin), antimycotics (e.g. toyocamycin, tubercidin), and photoprotectors (e.g. shinorin), and some even have anticancer properties (e.g. dolastatin, apratoxin) (5, 6). PKSs are also present in many other organisms besides cyanobacteria, and some are closely related to the *hgl* island but produce different biosynthetic products—particularly PKSs found in *Proteobacteria*, *Bacteroidetes*, and *Chloroflexi*, which include the polyunsaturated fatty acid synthase *pfa* which is responsible for the biosynthesis of polyunsaturated fatty acids (PUFAs) like eicosapentaenoic acid (EPA) and docosahexaenoic acid (DHA) (see section '*Hgl* and *hgl*-like islands are related to non-cyanobacterial PKSs involved in polyunsaturated fatty acid biosynthesis' below and Supplementary Fig. 4).

We hypothesized that genomic colocalization of homologs of HG biosynthesis genes in cyanobacterial genomes implies similar function in HG biosynthesis to the *Anabaena* sp. PCC 7120 *hgl* island, discerning them from PKSs that share protein domains but produce other products. Using a subset of 143 genomes of cyanobacteria with known morphological traits including their capacity for heterocyte formation (7), we confirmed that most heterocytous cyanobacteria (26 out of 28 in the subset) encode an island with homologous hits to at least 13 genes of the queried *Anabaena* sp. PCC 7120 *hgl* island (allowing for at most three open reading frames (ORFs) in between hits and multiple non-overlapping hits per ORF, see Materials and Methods; Supplementary Fig. 2). The two exceptions are *Raphidiopsis brookii* D9 (homologs of seven different genes) and *Calothrix parietina* PCC 6303 (homologs of eight different genes). *Raphidiopsis brookii* D9 has heterocytous ancestors and is therefore characterized as heterocytous cyanobacterium in the subset (7), but has lost the ability to fix nitrogen and is incapable of

heterocyte formation (8). The absence of a complete *hgl* island in its genome therefore likely reflects gene loss or genomic rearrangements due to loss of function (see section 'Evolutionary reconstruction of HG biosynthesis within *Cyanobacteriia*' below). The *hgl* island of *Calothrix parietina* PCC 6303 is interrupted by several CRISPR-associated proteins flanked by two CRISPR arrays, and its complete *hgl* island (homologs of 14 different genes; Supplementary Table 6) is therefore not detected by our genomic island search, which only allows for at most three ORFs in between hits.

In contrast, 109 of the 115 non-heterocytous cyanobacteria in the subset have at most homologs of three or less of the queried genes clustered on their genome (Supplementary Fig. 2). Only two of the 115 genomes have an island with homologs of more than five of the queried genes: the unicellular *Gloeobacter violaceus* PCC 7421 (homologs of seven different genes), whose homologs of *hgl* island genes with unknown function have been noticed before (2), and the unicellular *Gloeocapsa* sp. PCC 7428 (homologs of 10 different genes). We conclude that the ability to make heterocytes can be identified with high certainty from the genome sequence based on the presence of the here-defined *hgl* island. Based on this benchmark, we investigated islands with homologs of at least seven of the 19 queried genes as potentially involved in the biosynthesis of HGs, which we called *hgl* islands from hereon. This relatively permissive definition allowed us to detect PKSs that are likely involved in the biosynthesis of unknown HG-related products and reconstruct the evolutionary history of HG biosynthesis.

Next, we explored the *hgl* islands in our full set of cyanobacterial genomes (Supplementary Data 2). These included 3,579 genomes and plasmids from the PATRIC genome database (now part of the BV-BRC database) (Supplementary Data 1) and 14 genomes that were newly sequenced here (see Materials and Methods). The 14 sequenced genomes were from cultures of which we analyzed the lipids or whose lipids were analyzed in previous studies (see below) but that did not have their genome sequenced yet. Of the 3,593 genomes, 3,339 contained at least one hit to a queried protein, and 500 contained one or more *hgl* island(s) as defined here (i.e. a genomic cluster of homologs of at least seven of the queried HG biosynthesis genes) (Supplementary Data 2).

For further confirmation that the presence of an *hgl* island is associated with heterocyte formation, we screened the genomes for the presence of nitrogenase (*nif*) genes involved in the process of nitrogen fixation, which are also encoded by a genomic island in *Anabaena* sp. PCC 7120 (Supplementary Table 5; ref. (9)). This screen revealed that most genomes with an *hgl* island also encode a *nif* island (here defined as a genomic cluster of at least five *nif* genes; Supplementary Fig. 3), and are thus capable of nitrogen fixation.

Hgl and *hgl-like* islands are present throughout *Cyanobacteriia*

Identification of monophyletic heterocytous clade within Cyanobacteriia

We reconstructed the cyanobacterial species phylogeny based on a concatenated alignment of 24 conserved vertically-transferred genes (selected from ref. (10)) including one representative genome per species based on sequence similarity (average nucleotide identity ≥95%) and using *Vampirovibrionia* and *Sericytochromatia* genomes as outgroup for rooting (11, 12) (see Materials and Methods). Plotting the *hgl* islands on this phylogeny (Fig. 2a) revealed a large monophyletic group of cyanobacteria in which almost all genomes contain an *hgl* island composed of homologs of at least 10 of the queried genes, and which includes the genomes of known heterocyte-producing cyanobacteria mentioned earlier. We call this clade the 'heterocytous clade'. The clade contains uncultured genomes with unknown morphology. The most closely related sister clade to the heterocytous clade contains a single uncultured cyanobacterium from Antarctic soil (the metagenome-assembled genome *Nostocaceae* cyanobacterium MGR_bin409) that does not encode an *hgl* nor *nif* island and we thus assume is non-heterocytous. The most closely related sister clade to the heterocytous clade and *Nostocaceae* cyanobacterium MGR_bin409 contains genomes with known unicellular and baeocytous morphology and contains mainly non-diazotrophic cyanobacteria based on the absence of a *nif* island (Fig. 2a). Nevertheless, some of the inferred non-heterocytous genomes in this sister clade contain an *hgl* island (Fig. 2a; see Results and Discussion and below for a discussion on *hgl* islands in non-heterocytous cyanobacteria).

Gene conservation on hgl islands with homologs of at least 10 HG biosynthesis genes

First, we explored the genetic content of the identified islands. The genomic compositional variation between the islands includes gene absences and fusions/fissions, gene duplications, and positional rearrangements (Fig. 2a). For the *hgl* islands containing homologs of at least 10 queried genes, we examined the most common genes present. Homologs of 13 genes were present on ≥88% of these islands (Fig. 2c, Supplementary Fig. 6b), and their position on the island was largely conserved (Supplementary Fig. 7). In previous mutagenesis studies, 12 of the 13 genes have been shown to be essential to *Anabaena* sp. PCC 7120 for survival and growth in nitrogen-depleted conditions (Supplementary Table 4, Supplementary Fig. 6b; refs. (2–4, 13–18)). They include the epimerase *hgdA* and two genes encoding ATP-binding cassette (ABC) transporter proteins, *hgdC* and *hgdB*, responsible for transport of the HG and its deposition in the cell envelope; the gene encoding ketoacyl synthase, acyl transferase, and ACP domains, *hglE*$_A$; the gene *hglF* of unknown function; the gene *hglG* containing ketoreductase and dehydratase domains; the ketoacyl synthase *hglD*; the gene *hglC* containing a chain length factor and acyl transferase domains; the enoyl reductase *hglA*; and the gene *hglB* (also known as *hetM*), containing ACP and thioester reductase domains (Fig. 2c, Fig. 1, Supplementary Fig. 1). Additional studies have shown that although *hglT*, which encodes the glycosyltransferase (GT) that attaches the glucose

headgroup to the aglycone (AG), and *all5343*, a gene of unknown function, are required for proper heterocyte development and HG layer deposition, they are not essential for survival and growth in nitrogen-depleted conditions (Supplementary Table 4; refs. (2–4)). The role of *all5344* in HG biosynthesis has not yet been elucidated with mutagenesis studies. The 13 genes are likely evolutionary conserved on the *hgl* island to preserve function in heterocyte formation. The genes *all5343*, *all5344*, and *hglF* have yet unknown function but are present in 88%, 90%, and 99% of the *hgl* islands with homologs of at least 10 queried genes, respectively (Fig. 2c), which may further indicate an essential role in HG biosynthesis.

Genes that are often absent from the *hgl* islands may be non-essential for HG biosynthesis, be present somewhere else on the genome (Supplementary Fig. 8), or their catalytic function may be replaced by unrelated genes. For example, homologs of *alr5348,* encoding a protein with SAM-dependent methylase domains, and *asr5350*, encoding a protein of unknown function, are both clade-specific and only present in approximately half of the *hgl* islands containing homologs of at least 10 queried genes (Fig. 2a,c). The 4'-phosphopantetheinyltransferase (PPTase) *hetI* is present on 52% of these *hgl* islands. PPTases are essential enzymes required for the biosynthesis of a wide variety of compounds such as fatty acids, polyketides, and non-ribosomal peptides (19), and all genomes with diverse *hgl* islands encode *hetI* homologs, even if not always in the vicinity of the other HG biosynthesis genes (Supplementary Fig. 8). Many members of subclade 6 (see Fig. 2a) lack homologs of GTs and of proteins involved in HG deposition (*hgdACB*) on their *hgl* island. The GT *all5342* is rarely present on *hgl* islands (Fig. 2c), and *asr5349* is restricted to *Anabaena* sp. PCC 7120, reflecting clade-specific genes in our *hgl* island query.

Even though our protein queries represented individual open reading frames (ORFs), homologous sequences of *hglD* and *hglC*, and of *hglE*$_A$ and *hglF*, were often encoded by the same ORF (90% and 48% respectively, Fig. 2a,c), which was previously reported as anecdotical (2). The two independent ORFs of *hglE*$_A$ and *hglF* in *Anabaena* sp. PCC 7120 thus likely represent a rare gene fission from a single ancestral ORF containing the domains encoded by both genes.

The role of *hgl*-like island in heterocytous cyanobacteria is unclear

Our analysis thus reveals that HG biosynthesis genes are evolutionary conserved as a genomic cluster closely resembling the *Anabaena* sp. PCC 7120 *hgl* island—containing homologs of at least 10 of the queried genes—within a monophyletic heterocytous clade in *Cyanobacteriia*. These *hgl* islands are likely involved in HG biosynthesis and heterocyte formation. Moreover, various heterocytous cyanobacteria also encode an '*hgl*-like' island with a highly conserved gene composition containing homologs of only seven HG biosynthesis genes (the transporters *hgdCB* and PKS *hglE*$_A$*FGCA*) encoded by five ORFs in addition to a more extended *hgl* island (see Results and Discussion; Fig. 2, Supplementary Fig. 6c). The *hgl*-like island is not present in genomes from subclades 2, 3, and 6, but is notably present in subclade 1—the most basally branching heterocytous subclade—suggesting its

presence in the Last Heterocytous Cyanobacterial Common Ancestor (LHeCCA) (see section 'Evolutionary reconstruction of HG biosynthesis within *Cyanobacteriia*' below). Given that many heterocytous strains do not possess an *hgl*-like island (Fig. 2a) we suspect that it does not always have an essential function. However, its potential preservation during over 2 billion years of evolution in *Cyanobacteriia* suggests an important function in those strains where it is present.

Based on its prevalence in heterocytous cyanobacteria we hypothesized that the *hgl*-like island might produce and export compounds structurally similar to HGs which may also be associated with nitrogen fixation. However, genomic analysis revealed that the *hgl*-like island is likely not associated with nitrogen fixation. Certain strains such as *Raphidiopsis curvata* NIES-932, *Cylindrospermopsis raciborskii* CENA303, and the earlier discussed *Raphidiopsis brookii* D9 do not possess a *nif* island (Supplementary Data 2) and are thus incapable of nitrogen fixation even though they evolutionary branch from within the heterocytous clade (see also refs. (8, 20)). These non-diazotrophic strains possess a single *hgl* island that is more similar in sequence to the *hgl*-like islands of heterocytous cyanobacteria than to the more extended *hgl* island in those strains (see section 'Evolutionary reconstruction of HG biosynthesis within *Cyanobacteriia*' below). Thus, even though they have lost their more extended *hgl* island likely in relation to the loss of heterocyte formation, they have kept their *hgl*-like island, suggesting that it conveys a function independent of nitrogen fixation.

Since akinetes are thought to be the precursors of heterocytes (21) and are shown to be surrounded by the same layer of glycolipids and polysaccharides in some cultures (22, 23), we also speculated a potential role of the *hgl*-like island in akinete formation. However, mutational studies have shown that the HGs found in akinetes and heterocytes are synthesized by the same set of genes in *Anabaena variabilis* ATCC 29413 (17). In addition, our data show that not all strains that form akinetes possess an *hgl*-like island (e.g. *Anabaena variabilis* ATCC 29413). Therefore, we deemed this hypothesis unlikely. Thus, the potential role of the *hgl*-like island within heterocytous cyanobacteria remains unclear.

Heterocytous cyanobacteria produce a wide diversity of new HGs

Identification of new HGs in heterocytous cyanobacterial cultures

We analyzed HGs in the biomass of 24 heterocytous cyanobacterial cultures (Supplementary Table 10). In 23 of these cultures, HGs were previously reported. The cultures were evenly selected throughout the heterocytous cyanobacterial phylogeny and included representatives from all subclades in Fig. 2a with the exception of subclade 1.

HG biosynthesis involves several rounds of condensation, reduction, and dehydration of acyl-CoA precursors (Supplementary Fig. 1), and different combinations of these steps result in a large

theoretically possible number of structures. We screened the lipid extracts of the cultures for 88 theoretically possible HG structures including 19 HGs previously described in the literature, using ultra-high-performance liquid chromatography coupled with multistage high-resolution mass spectrometry (UHPLC-HRMS^n). A wide range of HGs were detected (Supplementary Table 10, Supplementary Data 5), including the 19 HGs previously described in the literature, which had been identified from their accurate masses and mass spectral fragmentation using other techniques such as high performance liquid chromatography coupled to electrospray ionization tandem mass spectrometry (HPLC–ESI-MS^2, refs. (24, 25)) or ultra-high performance liquid chromatography coupled to an ultra-high-resolution quadrupole time-of-flight (QToF) mass spectrometer (26). In addition, 30 HGs were detected that, to the best of our knowledge, have not previously been described in the literature (Supplementary Table 10). With UHPLC-HRMS^n, we thus detected a wider diversity of HGs than previously identified in these cultures, particularly HGs occurring in low relative abundance (Supplementary Fig. 9).

The previously reported HGs included thirteen hexose HGs with either 26, 28, 30 or 32 *n*-alkyl chains and with either two (keto-ol, diol, or diketone) or three (triol and keto-diol) oxygen moieties on their alkyl chain (24–28). Additionally, we detected several HGs with alternative sugar moieties that had been previously described in the literature, including four pentose HGs (pentose HG_{26} diol, pentose HG_{30} diol, pentose HG_{30} triol, and pentose HG_{32} triol (26, 29, 30), plus the previously described methyl-hexose HG_{28} (ref. (31)), and deoxy-hexose HG_{26} (ref. (26)). We note that, while 'pentose' refers to a C_5 sugar, the pentoses detected in the cultures in this study may be in the pentopyranose form (see also section 'Potential origin of HGs from ancient 1,3-diols') instead of the furanose detected in *Richelia* species (29) .

Of the 30 novel HGs detected in this study, 22 HGs had a known sugar moiety and either a 26, 28, 30 or 32 carbon alkyl chain with alcohol or keto moieties—as described above for the known HGs, but in combinations not previously described. These were thus straightforward to identify from their accurate masses, their fragmentation patterns, and their elution order (Supplementary Table 9). Six of these were hexose HGs, six were pentose HGs, eight were methyl-hexose HGs and one was a deoxy-hexose HG. Finally, we found nine novel HGs with more unusual structures (Supplementary Table 10, Supplementary Fig. 9), which we discuss below.

One of the unusual novel HGs gave rise to a dominant ion at mass-to-charge ratio (*m/z*) 576.483, assigned as $C_{32}H_{66}O_7N$. Under MS^2 fragmentation (Supplementary Fig. 10a), this ion underwent neutral loss of 179.080 Da ($C_6H_{13}O_5N$), indicative of a hexose and NH_3 loss from a $[M+NH_4]^+$ ion, to produce a fragment ion at *m/z* 397.403 ($C_{26}H_{53}O_2$). This fragment ion also underwent a subsequent loss of H_2O to produce an ion at *m/z* 379.393 ($C_{26}H_{51}O$), followed by another loss of H_2O resulting in an ion at *m/z* 361.382 ($C_{26}H_{49}$). The combination of these neutral losses provides evidence that the parent ion at *m/z* 576.483 is the $[M+NH_4]^+$ of a hexose HG_{26} keto. To the best of our knowledge, HGs with a single

oxygen moiety on the alkyl chain have not previously been reported. Similarly, a second novel HG gave rise to an ion at *m/z* 578.499 ($C_{32}H_{68}O_7N$), exhibited a similar fragmentation in MS^2 (Supplementary Fig. 10b) as the previously described compound, but eluted several minutes earlier. Hence, this component was assigned as a hexose HG_{26} ol (see also section ‘Potential origin of HGs from ancient 1,3-diols’ below). One or both of these two components were detected in low levels (<2% of total HG sum) in six cyanobacterial cultures.

Three structurally similar components gave rise to ions at *m/z* 617.462, *m/z* 645.494 and *m/z* 673.525. All three contained a hexose sugar moiety, as evidenced by the primary neutral loss of 162.053 Da ($C_6H_{10}O_5$) in MS^2 (Supplementary Fig. 11). The neutral loss of the sugar gave rise to primary fragments ions at *m/z* 455.409 ($C_{28}H_{54}O_4$), *m/z* 483.441 ($C_{30}H_{58}O_4$), and *m/z* 511.472 ($C_{32}H_{62}O_4$), respectively. These all underwent four sequential losses of H_2O. Based on their accurate masses and their fragmentation patterns, these three components were assigned as hexose HG_{28} diketo-ol, hexose HG_{30} diketo-ol, and hexose HG_{32} diketo-ol, respectively.

Another unusual HG gave rise to an assumed $[M+H]^+$ ion at *m/z* 591.484 ($C_{33}H_{67}O_8$) and was detected in eight cyanobacterial cultures. This HG contained a hexose sugar moiety (as revealed from the 162.053 Da neutral loss from the parent ion) in MS^2 (Supplementary Fig. 12a). This neutral loss led to the formation of a fragment ion at *m/z* 429.430, equivalent to $C_{27}H_{57}O_3$. This suggests that this HG contains a 27-carbon alkyl chain, in contrast to all previously described HGs that have even-numbered alkyl chains. From the *m/z* 429.430 fragment ion there were consecutive losses of H_2O, giving rise to ions at *m/z* 411.418 ($C_{27}H_{55}O_2$), *m/z* 393.408 ($C_{27}H_{53}O$) and *m/z* 375.397 ($C_{27}H_{51}$), a fragmentation pattern similar to that regularly described for HG diols (24) . Hence, this novel HG was assigned as a hexose HG_{27} diol. Similarly, another novel ‘HG-like’ component gave rise to a $[M+H]^+$ at *m/z* 617.499 ($C_{35}H_{69}O_8$), which underwent loss of a hexose moiety in MS^2 (Supplementary Fig. 12b), resulting in fragment ions with a C_{29} chain. This novel component, which was detected in two cultures (*Tolypothrix tenuis* PCC 7101 and *Fortiea contorta* PCC 7126), was assigned as a hexose HG_{29} keto-ol.

Another novel HG had an $[M+H]^+$ at *m/z* 633.530 ($C_{35}H_{69}$O9) and was detected in just one culture (*Scytonema* sp. PCC 10023). In MS^2 (Supplementary Fig. 13a) this ion gave rise to a fragment ion at *m/z* 501.488 ($C_{30}H_{61}O_5$), due to a neutral loss of 132.043 Da ($C_5H_8O_4$), characteristic of a pentose sugar moiety (29). The fragment ion at *m/z* 501.488 unusually underwent five subsequent losses of H_2O, resulting in fragment ions at *m/z* 483.440 ($C_{30}H_{59}O_4$), *m/z* 465.429 ($C_{30}H_{57}O_3$), *m/z* 447.419 ($C_{30}H_{55}O_2$), *m/z* 429.409 ($C_{30}H_{53}O$), and finally *m/z* 411.399 ($C_{30}H_{51}$), indicative of a component with four oxygen moieties on the alkyl chain. Hence this novel HG was assigned as a pentose HG_{30} keto-triol.

Finally, a novel HG-like compound was detected in three cultures (*Chlorogloeopsis fritschii* PCC 6912, *Chlorogloeopsis fritschii* CCY 9924, and *Fischerella muscicola* PCC 73103) and gave rise to an apparent $[M+H]^+$ ion at *m/z* 613.504. The MS^2 spectrum arising from fragmentation of this ion

(Supplementary Fig. 13b) revealed a fragmentation pattern characteristic of an HG with a C_{30} keto-ol component, e.g., dominant ions at *m/z* 469.463 ($C_{30}H_{61}O_3$), *m/z* 451.450 ($C_{30}H_{59}O_2$), *m/z* 433.440 ($C_{30}H_{57}O$) and *m/z* 415.429 ($C_{30}H_{55}$). However, the initial neutral loss normally associated with loss of the sugar moiety was 144.043 Da, equivalent to $C_6H_8O_4$. Structural identification of the assumed sugar moiety was not possible based on its accurate mass and hence the component is assigned as an HG-like, C_{30} keto-ol with an unknown headgroup.

On the other hand, out of the 88 theoretically possible HG structures that we screened for, 39 HGs were not detected in the cultures. These include hexose HG_{26}, HG_{28}, HG_{30} or HG_{32} without functional groups. Additionally, no hexose HG_{26} diketo-ols or keto-diols were detected, nor HG_{28}, HG_{30} or HG_{32} containing only one functional group (neither ketone nor alcohol) or three ketone groups. The pentose HGs that were screened for but were not detected include pentose HG_{26} diol, pentose HG_{28} keto-diol, pentose HG_{30} keto-ol, pentose HG_{30} diketone, pentose HG_{32} keto-ol, and pentose HG_{32} diol. The methyl-hexose and deoxy-hexose HGs that were searched for but not detected include methyl-hexose HG_{28} diol, methyl-hexose HG_{30} diketone, methyl-hexose HG_{30} keto-diol, methyl-hexose HG_{32} keto-ol, methyl-hexose HG_{32} diol, methyl-hexose HG_{32} keto-diol, deoxy-hexose HG_{28} keto-ol, deoxy-hexose HG_{28} diol, deoxy-hexose HG_{28} keto-diol, deoxy-hexose HG_{28} triol, deoxy-hexose HG_{30} keto-ol, deoxy-hexose HG_{30} diol, deoxy-hexose HG_{30} diketone, deoxy-hexose HG_{30} keto-diol, deoxy-hexose HG_{30} triol, deoxy-hexose HG_{32} keto-ol, deoxy-hexose HG_{32} diol, deoxy-hexose HG_{32} keto-diol, and deoxy-hexose HG_{32} triol (Supplementary Table 8).

These undetected HGs may be selected against because of ecological reasons, or because of biosynthetic restrictions. For example, we speculate—pending future membrane mechanistic studies—that the broad absence of HG_{26}'s with three functional groups (only hexose HG_{26} triol was detected, with a relative abundance <1% in four cultures) may reflect that the addition of a third functional group to relatively short HGs does not improve the protective function of the membrane against O_2 or disrupts membrane packing. As another example, variation in the abundance of hexose HG_{26} diols relative to hexose HG_{26} keto-ols has been associated with temperature adaptation (with a higher proportion of keto-ols produced at higher temperatures, likely to limit the flux of O_2 into the heterocyte) (32), yet hexose HG_{26} diketones are rarely detected and only in low abundances. Thus, even though the presence of keto groups in HGs as keto-ols may limit the flux of O_2 into the heterocyte, diketones may not have the same effect. Other combinations of headgroup, chain length, and functional groups may strongly influence membrane stability or have thus far unknown ecological implications that are selected against. Alternatively, the absence of 39 theoretically possible HGs in our cultures may also reflect the specific growth conditions of our experiments, and a different set of culturing conditions (e.g. different temperature, agitation, or light availability) or different sampling time in the growth curve, may reveal the production of some of these HGs. Finally, some of the HGs may be produced by the cultures in amounts below the current detection limit of our analytical techniques.

HG structure is mostly independent of *hgl* island gene composition

HG abundances and distribution throughout the heterocytous cyanobacterial phylogeny

Next, we studied the distribution of the detected HGs across the 24 cultures analyzed. We focused on the three characteristics of the HG structures: headgroup, functional groups on the alkyl chain, and alkyl chain length.

Headgroups. In all the cultures analyzed, hexose was the most abundant HG headgroup, followed by pentose (possibly in pentopyranose form), deoxyhexose, and methyl-hexose (Supplementary Table 10). The capacity to produce HGs with three different sugar headgroups was present in strains scattered across the heterocytous cyanobacterial phylogeny (Fig. 3). In general, HGs with alternative headgroups to hexose appeared in small amounts (<0.1 - 3.8%) and usually only in conjunction with the abundant production (>20%) of that same HG with a hexose headgroup. However, in some cases HGs with headgroups other than hexose were found in relatively high abundances such as pentose HG_{26} diol in *Nostoc* sp. CCY 0012 (14.4%), deoxy-hexose HG_{26} diol in *Anabaena* sp. CCY 0017 (20.1%) and *Nodularia chucula* CCY 0103 (7.9%), methyl-hexose HG_{28} triol in *Calothrix* sp. CCY 0202 (17.2%), and methyl-hexose HG_{30} triol in *Tolypothrix tenuis* PCC 7110 (16.8%). In addition, pentose HG_{30} keto-triol was the only HG with a pentose headgroup for which a hexose equivalent has not been detected, neither here nor in previous studies (Supplementary Tables 10).

Functional groups. Overall, most cultures analyzed synthesized most of their HGs (>93%) with either two (diol and keto-ol) or three (keto-diol and triol) functional groups on the alkyl chain besides their sugar moiety. Only one of the analyzed cultures, *Scytonema* sp. PCC10023, produced an HG with four functional groups (pentose HG_{32} keto-triol, 2.7%). Cultures of strains found in subclades 2-4 (Fig. 3, Supplementary Fig. 9) produced mostly HGs with three functional groups, usually keto-diols and triols, but also in some cases diketo-ols. Cultures of strains in subclades 5-9 (Supplementary Fig. 9) mostly produced HGs with only two functional groups, such as diols and keto-ols, but they also synthesized diketones and HGs with only one keto or alcohol group. Even though we found clade-specific dominance of the number of functional groups, HGs with two and three functional groups were also found in low abundance throughout subclades 2-4, and subclades 5-9, respectively. Strains capable of producing diketones (with or without an additional alcohol group) were also found throughout the phylogeny.

Chain length. Although most cultures analyzed produced most (>91%) of their total HGs with only one specific *n*-alkyl chain length, they were also capable of producing HGs with different *n*-alkyl chain lengths. One exception to the dominance of a single chain length was *Tolypothrix tenuis* PCC 7101, a strain found in subclade 6 (Supplementary Fig. 9), which produced hexose HG_{28} and hexose HG_{30} in

similar proportions (42% and 57%, respectively). Notably, cultures of strains from subclades 2-4 were capable of producing $HG_{28,30,32}$, whilst cultures of strains from subclades 5-9 usually produced $HG_{26,28}$ (Supplementary Fig. 9). An exception was again *Tolypothrix tenuis* PCC 7101, which besides hexose HG_{28} also produces hexose HG_{30}. Although cultures from closely related strains usually produced HGs of the same length, this was not always the case. For example, *Nostoc* sp. CCY 9925 and *Nostoc* sp. CCY 9926 (both from subclade 9) synthesized hexose HG_{28} and hexose HG_{26}, respectively. Additionally, odd-chain HGs were only found in cultures of strains from subclades 5-9 (Fig. 3, Supplementary Fig. 9, Supplementary Table 10).

HglT is absent from pentose HG producing symbiotic cyanobacteria

As mentioned above, the protein product of *hglT* is responsible for attaching the sugar headgroup to the AG in the last step of HG biosynthesis by *Anabaena* sp. PCC 7120. However, homologs of this gene are not always present on the *hgl* islands (Fig. 2c).

The alignment scores of hits obtained during our homology search of the *hglT* of *Anabaena* sp. PCC 7120 show a bimodal distribution, where hits with a bit-score >350 are mostly located on the *hgl* island (Supplementary Fig. 14, orange bars). All *hglT* hits that are on an *hgl* island have a high bit-score, suggesting that the bit-score reflects conserved enzymatic functionality. In contrast, most genomes in which the best hit for *hglT* has a bit-score <200 do not contain an *hgl* island (Supplementary Fig. 14, purple bars). Hence, hits with low-scoring alignments may be indicative of distant homologs with a potentially divergent function unrelated to HG biosynthesis. Incidentally, there are some genomes with high-scoring *hglT* hits that do not contain the homolog on their island (Supplementary Fig. 14, cyan bars with bit-score >350) or that do not contain an *hgl* island (Supplementary Fig. 14, purple bars with bit-score >350). This could be due to (based on manual curation) fragmented assemblies of genomes (in which case the *hgl* island is absent or located close to a contiguous sequence (contig) edge), or disruption of the *hgl* island for example by transposases or by CRISPR arrays (e.g *Calothrix parietina* PCC 6303 discussed above, Supplementary Table 6). We assume that these genomes encode conventional *hglT* activity even though its homolog is not found within an *hgl* island based on our automated searches. However, there are some cyanobacteria that do contain an *hgl* island but lack a high-scoring *hglT* hit anywhere on their genome (Supplementary Fig. 14, cyan bars with bit-score <200). For example, high-scoring *hglT* hits are absent from *Richelia euintracellularis* HM01*, Richelia intracellularis* RC01 and *Richelia rhizosoleniae* SC01 genomes. These three cyanobacteria form symbiotic relationships with marine diatoms, and previous studies have shown that *Richelia euintracellularis* HM01 and *Richelia intracellularis* RC01 exclusively produce HGs with pentose headgroups in the furanose form (29, 30), in contrast to other analyzed cultures which predominantly produce hexose HGs (Fig. 3). The HG profile of *Richelia rhizosoleniae* SC01—a symbiont of the marine diatom *Chaetoceros compressus*—is not known, but since it also does not encode a high-scoring *hglT* hit on its genome, we predict that it also produces pentose HGs like the other two *Richelia* species.

We hypothesized that a gene other than a high-scoring *hglT* homolog with substrate specificity for pentose instead of hexose (hereafter *hglT2*) is responsible for the addition of the sugar headgroup to the AG in these species. To find the gene responsible in *Richelia euintracellularis* HH01, an endosymbiont of *Hemiaulus hauckii*, we used two approaches: (a) sequence homology to *hglT*, and (b) functional annotation as a GT and colocalization on the *hgl* island. Each approach returned two candidate genes in *Richelia euintracellularis* HH01: (a) *RINTHH_17770* and *RINTHH_20790*, and (b) *RINTHH_5560* and *RINTHH_5570.* The candidate genes were heterologously expressed in an *Anabaena* sp. PCC 7120 strain that could only produce AGs and no HGs due to the deletion of *hglT* (Δ*hglT*) (4) (see Materials and Methods, Supplementary Figs. 15-17 and Supplementary Tables 11-15, Supplementary Data 6). Since *RINTHH_5560* and *RINTHH_5570* were only separated by 42 base pairs (bp), we expressed them together including their intergenic region. *Anabaena* sp. PCC 7120 *wild-type* (*wt*) was reported to only produce hexose HGs (33), while here for the first time we also detected the presence of a very low amount of pentose HGs in the *wt* phenotype (0.28%; Supplementary Table 15), although possibly in the pentopyranose form instead of the furanose found in *Richelia* species. Hence, upon expression of *hglT2* in the HG-deficient strain, we expected mainly production of HGs with a pentose headgroup.

However, although the control strain expressing the deleted *hglT* on a plasmid (ARP001) did recover the original *wt* phenotype, producing hexose HGs (97%) and small amounts of pentose HG_{26} (0.19%), none of our candidate genes led to the generation of pentose HGs (Supplementary Table 15). Low amounts of hexose HG_{26} diol (<0.17%) were still detected in strain Δ*hglT* and in all mutants generated in this study (Supplementary Table 15), potentially caused by spontaneous reaction or by the unspecific activity of another GT. There are several possible explanations for why no pentose HGs were detected in any of the mutants: (i) none of the candidate genes is *hglT2*; (ii) we did not express the functional sequence, as for example the ORF of *RINTHH_20790* differs when using different gene calling methods (See Materials and Methods); (iii) *Richelia euintracellularis* HH01 proteins are not functional in *Anabaena* sp. PCC 7120 due to physiological differences potentially affecting their folding and catalytic activity (e.g. intracellular pH, redox potential, absence of required chaperones, cofactors or ligands, etc.); (iv) limited availability of free pentose sugars in *Anabaena* sp. PCC 7120, and (v) *hglT2* acts as a molecular caliper and can only catalyze the reaction when the substrate AG has the correct chain length (note that *Richelia euintracellularis* HH01 produces $HG_{30,32}$, and *Anabaena* sp. PCC 7120 synthesizes $HG_{26,28}$). Given that strains where *hglT* is expressed (*wt* and ARP001) do synthesize small amounts of pentose HGs, and that *Anabaena* sp. PCC 7120 is known to have abundant pentoses (34), we consider explanation (iv) unlikely. To test explanation (v), one could express the GT of a known hexose HG_{30} or hexose HG_{32} producer (such as *Chlorogloeopsis fritschii* PCC 6912) in the Δ*hglT* background strain. If no hexose HGs are produced, this would suggest that the chain length of the AG is a determining factor in the GT activity, but it would not rule out possible explanations (i), (ii), or (iii).

HG structure evolved convergently

Re-evaluating HGs as biomarkers

For years, the chemical structure of HGs and their relative abundance were thought to have chemotaxonomical value and were used to distinguish between cyanobacteria of different orders and families (28, 30–32). However, the increasing number of strains analyzed and the recent changes in cyanobacterial taxonomy, combined with our high-resolution lipid analysis of 24 heterocytous cultures, are challenging this approach. For example, large concentrations of hexose HG_{26}'s coupled with small amounts of hexose HG_{28}'s were associated with the former *Nostocaceae* family (32) (currently *Nostocaceae* and *Aphanizomenonaceae* families according to the latest taxonomic classification (35, 36) (Supplementary Table 16)). However, in some members of these families HG_{28}'s account for a large proportion of the total HGs (e.g. 46% and 12%, in *Dolichospermum* sp. BIR169 and *Aphanizomenon* sp. TR83, respectively) (37). Another example is the recent identification of hexose HG_{28} diketone in *Microchaete* sp. PCC 7126 (currently *Fortiea contorta*, placed in subclade 6) (38) and its proposal as potential biomarker to track the *Microchaete* genus (currently representing members of the *Microarchaete* and *Fortiea* genera) (25). However, here we show that this diketone is also produced by *Anabaena* and *Nostoc* strains from subclades 5 and 9, respectively (Supplementary Table 10, Supplementary Fig. 9), hence rendering it unusable as an exclusive taxonomic biomarker for the *Fortiea* or the *Microchaete* genera. Additionally, the evolutionary evidence presented in this study suggests that the HG profiles observed in extant cyanobacteria resulted from convergent evolution (Fig. 3 and Supplementary Figs. 9 and 23), a process where the same trait evolves independently in different species likely triggered by environmental factors, thus further challenging their use as taxonomy biomarkers. For instance, survival under extreme temperatures or high oxygen concentrations might have been only possible in strains capable of producing HGs with 'long' alkyl chains, which might provide increased structural integrity and impermeability (27, 29).

Hgl islands predate heterocyte formation

Hgl and hgl-like islands provide genomic evidence to elucidate the origin of HGs within Cyanobacteriia

As mentioned above (see also Results and Discussion), HG biosynthesis genes were evolutionary conserved as a genomic cluster closely resembling the *Anabaena* sp. PCC 7120 *hgl* island—containing homologs of at least 10 of the queried genes—within a monophyletic heterocytous clade in *Cyanobacteriia*. In addition, several distantly related non-heterocytous cyanobacteria also encode *hgl* islands with a more diverged gene composition, and various heterocytous cyanobacteria encode an additional *hgl*-like island with a highly conserved gene composition containing homologs of only seven

HG biosynthesis genes encoded by five ORFs (see Results and Discussion and Fig. 2). The broad presence of *hgl* and *hgl*-like islands in heterocytous and non-heterocytous cyanobacteria provides genomic evidence to elucidate the origin of HGs within *Cyanobacteriia*. We will first discuss the relationship of the *hgl* and *hgl*-like islands to other PKSs found in *Cyanobacteriia* and in other taxonomic groups, and then reconstruct their evolution in *Cyanobacteriia* via the phylogeny of homologs of *hglE$_A$*, the longest gene in the queried *hgl* island, and the phylogeny of a longer concatenated alignment of homologs of seven HG biosynthesis genes that are shared among most *hgl* and *hgl*-like islands (*hgdCB* and *hglE$_A$FGCA*).

Hgl and hgl-like islands are related to non-cyanobacterial PKSs involved in polyunsaturated fatty acid biosynthesis

To assess the evolutionary relationship between *hgl* and *hgl*-like islands of heterocytous cyanobacteria to other PKSs in cyanobacteria and in other taxonomic groups, we screened the 225,388 prokaryotic genomes from the PATRIC genome database (now part of the BV-BRC database) with at least medium-quality draft status (estimated completeness ≥50% and contamination <10%) for clusters of homologs of HG biosynthesis genes (Supplementary Fig. 4, Supplementary Data 3), applying the same approach we used earlier to identify these clusters in *Cyanobacteriia*. The genomes represent a wide diversity of prokaryotes, with representatives of 182 prokaryotic phyla. We evaluated the clusters in the context of the best *hglE$_A$* hit, as this is the longest gene in the queried *hgl* island, it encodes ketoacyl synthase and acyl transferase domains (Supplementary Fig. 1) and is thus likely an essential element of related PKSs.

This screen revealed that all clusters of HG biosynthesis genes that contain *hglE$_A$* hits with a high alignment score (bit-score >1,500) are found in cyanobacteria (Supplementary Fig. 4a), and in almost all cases they are present in an *hgl* island according to our definition (i.e. a gene cluster containing homologs of at least seven of the 19 queried genes) (Supplementary Fig. 4b). The *hglE$_A$* hits in *hgl*-like islands (that contain homologs of seven of the queried genes) have a lower bit-score (~1,450-1,850) than the *hglE$_A$* hits in most other *hgl* islands (Supplementary Fig. 4b). The bit-score region between 1,150 and 1,450 contains the *hglE$_A$* homologs on some *hgl* islands and hits within cyanobacteria that are not part of an *hgl* island by our definition (Supplementary Fig. 4b), which are found in 11 genera but primarily in the genus *Microcystis* (Supplementary Fig. 4e). The region also includes an *hglE$_A$* homolog within Anabaena sp. PCC 7120 itself (bit-score 1,216) that is not part of the queried *hgl* island; this hit is encoded on a cluster containing homologs of five different queried genes and thus also has a different gene composition than the *hgl*-like islands. This *hglE$_A$* homolog had been previously identified as *hglE2* (14), and although its product is unknown, it has been experimentally shown not to be involved in the formation of the HG layer (14). In addition, many cyanobacteria contain *hglE$_A$* hits that are not present in an *hgl* island by our definition and have a relatively low bit-score (<900) (Supplementary Fig. 4b).

We infer that these *hglE*$_A$ hits with a bit-score <900 may be part of more distantly-related cyanobacterial PKSs that are involved in the biosynthesis of other products than HGs or structurally related molecules.

Our screening throughout the tree of life also identified clusters of homologs of HG biosynthesis genes outside cyanobacteria that contain similarly low-scoring *hglE*$_A$ hits (Supplementary Fig. 4c), which we likewise attribute to distantly-related PKSs producing other products than HGs or structurally related molecules. However, some non-cyanobacterial *hglE*$_A$ hits have a relatively high bit-score (≥1,150) (Supplementary Fig. 4c), suggestive of a close evolutionary relationship to the *hgl* and *hgl*-like islands in heterocytous cyanobacteria. Clusters with high-scoring *hglE*$_A$ hits are found in the phyla *Proteobacteria*, *Bacteroidetes*, and *Chloroflexi* (Supplementary Fig. 4d), and are restricted to specific genera—within *Proteobacteria* primarily to *Vibrio*, *Shewanella*, *Moritella*, *Colwellia*, and *Psychromonas* (Supplementary Fig. 4f). Only two non-cyanobacterial genomes encode *hglE*$_A$ hits with a bit-score >1,450—both metagenome-assembled genomes (MAGs) of unknown *Chloroflexi* genera (bit-scores 1,460 and 1,471).

The biosynthetic products of some of these high-scoring non-cyanobacterial *hglE*$_A$ hits are known or suggested. They include the omega-3 polyunsaturated fatty acid synthase gene *pfaA* of *Photobacterium profundum* SS9 (39, 40) (bit-score 1,187), *Moritella marina* (formerly *Vibrio marinus*) (41) (bit-score 1,294 for strain ATTC 15381), *Shewanella oneidensis* MR-1 (42) (bit-score 1,168), and other previously suggested *pfaA* genes (43), for example of *Shewanella baltica*, *Shewanella benthica*, *Shewanella denitrifcans*, *Shewanella halifaxensis*, *Vibrio* sp. MED222, *Vibrio splendidus*, and *Colwellia psychrerythraea* 34H (Supplementary Data 3). In these bacteria, the *hglE*$_A$ homologs are part of a PKS (called *pfa* synthase) that is responsible for the production of polyunsaturated fatty acids (PUFAs) like eicosapentaenoic acid (EPA) and docosahexaenoic acid (DHA), which have antioxidative roles (44–47) and may be beneficial to facultative and strict anaerobic bacteria (48).

Some known or suggested *pfaA* genes have a bit-score <1,150 to the *hglE*$_A$ query, for example the *pfaA* homologs of *Shewanella pealeana* ATCC 700345 (40) (bit-score 978), arachidonic acid-producing *Aureispira* species (49) (bit-score ≤1,100), *Geobacter bemidjiensis* Bem (48) (bit-score 771), and *Psychroflexus torquis* ATCC 700755 (50) (bit-score 1,043), suggesting a large diversity of *pfaA* genes—some of which are more closely related to *hglE*$_A$ than others.

Finally, we searched in our dataset for the best *hglE*$_A$ hit in *Azotobacter vinelandii* (five strains in our dataset), which makes dormant cysts that contain fatty acid-based phenolic lipids (51), and phenolic lipids may serve a similar function in *Azotobacter vinelandii* as the HGs in heterocytes and akinetes serve in cyanobacteria. However, the low alignment score of this *arsA* gene to the *hglE*$_A$ query (a bit-score of 629 for *Azotobacter vinelandii* NBRC 13581 and of 628 for *Azotobacter vinelandii* CA, CA6, DJ, and DSM 279) suggests that it is not as closely related to *hglE*$_A$ as *pfaA*, and the supposedly similar

role of their biosynthetic products is thus likely due to convergent evolution rather than shared ancestral function.

We conclude that the *hglE*$_A$ homologs on *hgl* and *hgl*-like islands of heterocytous cyanobacteria are closely related to *hglE*$_A$ homologs on PKSs of heterocytous and non-heterocytous cyanobacteria (including the non-heterocytous *hgl* islands, and the *hglE2*-containing cluster of *Anabaena* sp. PCC 7120) and to *hglE*$_A$ homologs on PKSs involved in PUFA biosynthesis in specific genera of *Proteobacteria*, *Bacteroidetes*, and *Chloroflexi*, confirming previous suggestions of the evolutionary close association between PUFA and HG biosynthesis (43, 48).

Phylogeny of hglE$_A$ *homologs reveals single origin in Cyanobacteriia*

To investigate the evolutionary history of HG biosynthesis within *Cyanobacteriia*, we constructed a phylogeny of *hglE*$_A$ homologs including representatives from: (i) *hgl* and *hgl*-like islands of heterocytous cyanobacteria, (ii) *hgl* islands of non-heterocytous cyanobacteria, (iii) cyanobacterial homologs that were not present in an island by our definition but had a bit-score ≥1,150 (such as *Microcystis* homologs and *hglE2*), and (iv) homologs from *Proteobacteria*, *Bacteroidetes*, and *Chloroflexi* with a bit-score ≥1,150 (such as *pfaA*). In this phylogeny (Supplementary Figs. 24 and 25, Supplementary Data 8), *Cyanobacteriia* form a clear distinct group from *Proteobacteria*, *Bacteroidetes*, and *Chloroflexi*—the exception being a single *Proteobacteria* genome that branches from within *Cyanobateriia* (*Beggiatoa* sp. 4572_84, grey star in Supplementary Figs. 24 and 25) and whose placement within *Cyanobacteriia* we attribute to horizontal transfer, cyanobacterial contamination of the MAG (estimated contamination 7.8%, based on the PATRIC 'genome_summary' file), or taxonomic misannotation. To test the last two explanations, we taxonomically annotated the MAG and the contig on which the *hglE*$_A$ hit was located with Bin Annotation Tool (BAT) and Contig Annotation Tool (CAT), respectively (52). Both the MAG and the contig were annotated as *Gammaproteobacteria*, suggesting that the genome is indeed from a gammaproteobacterium and the contig did not originate from cyanobacterial contamination. This suggests that a horizontal transfer event from *Cyanobacteriia* is a likely explanation for the presence of this *hglE*$_A$ homolog in *Beggiatoa* sp. 4572_84.

When a root is placed in between the *hglE*$_A$ hits of *Cyanobacteriia* and the non-cyanobacterial hits (those of *Proteobacteria*, *Bacteroidetes*, and *Chloroflexi*), the deepest branching cyanobacterial *hglE*$_A$ hits are from *Gloeobacteraceae* cyanobacterium 'cyano 109', *Aphanocapsa lilacina* HA4352-LM1, and *Gloeobacter violaceus* PCC 7421 (Supplementary Figs. 24 and 25), which are also the deepest branching *Cyanobacteriia* with an *hgl* island in the phylogeny created using the concatenated alignment of 24 core vertically-transferred genes (Fig. 2a, Supplementary Fig. 21b). This suggests a correct root placement and single origin of *hglE*$_A$ homologs and associated PKSs within *Cyanobacteriia*, likely predating the radiation of the crown group of *Cyanobacteriia*. The *hgl* islands in deeply-branching cyanobacterial genomes are thus likely ancestral remnants rather than more recent transfers from around the time of

LHeCCA. In this scenario, the absence of *hgl* islands in most contemporary non-heterocytous cyanobacteria can be explained by widespread loss.

In addition, the phylogeny of *hglE*$_A$ homologs shows evidence of multiple ancient duplications, as several genomes contain multiple *hglE*$_A$ hits belonging to distinct deeply-branching phylogenetic clusters (Supplementary Fig. 25)—including a duplication that led to the two types of islands on single genomes of heterocytous cyanobacteria (*hgl* and *hgl*-like islands) (see also section 'Evolutionary reconstruction of HG biosynthesis within *Cyanobacteriia*' below). The cyanobacterial *hglE*$_A$ homologs that are not part of an *hgl* island by our definition form separate clades from *hglE*$_A$ homologs of most *hgl* islands, and include the previously mentioned *Microcystis* hits as well as *hglE2* and a closely related hit in *Anabaena variabilis* ATCC 29413 (Supplementary Fig. 25). The clusters of homologs of HG biosynthesis genes on which these *hglE*$_A$ homologs are located do not encode homologs of the *hgdCB* transporters in most cases, whereas those identified as *hgl* islands often do encode homologs of the transporters (Supplementary Fig. 25). The genomes that encode these *hglE*$_A$ homologs that are not part of an *hgl* island often also do encode an *hgl* island (like *Anabaena* sp. PCC 7120 and *Anabaena variabilis* ATCC 29413). Their distant placement from the *hgl* and *hgl*-like islands in the *hglE*$_A$ homologs phylogeny suggests that they arose from a duplication that postdated the split of the basal *Cyanobacteriia* like *Gloeobacter violaceus* PCC 7421 and the other *Cyanobacteriia* and predated the duplication that led to *hgl* and *hgl*-like islands in heterocytous cyanobacteria (Supplementary Fig. 25).

However, the phylogeny of *hglE*$_A$ homologs cannot be fully explained by vertical transfer, loss, and duplications. For example, *Gloeobacteraceae* cyanobacterium 'cyano 109' contains another *hglE*$_A$ homolog in its genome that is closely related to the *hglE*$_A$ homologs found in the heterocytous *hgl*-like islands (Supplementary Fig. 25), and many heterocytous and non-heterocytous homologs are interspersed in the phylogeny (Supplementary Figs. 24 and 25). The cases in which the phylogeny of *hglE*$_A$ does not follow the vertically transferred part of the genome, are evidence of ancient or more recent horizontal gene transfers. In any case, horizontal gene transfers were always restricted within the *Cyanobacteriia*, except for a potential (above discussed) transfer from *Cyanobacteriia* to (the ancestor of) *Beggiatoa* sp. 4572_84. In line with this phylogenetic evidence of horizontal transfer, we identified one plasmid (pNFSY07) of *Nostoc flagelliforme* CCNUN1, a strain isolated from desert soil of Inner Mongolia (53), containing a cluster of homologs of five queried genes (*hglE*$_A$*FGCA*; see Supplementary Data 1 and 2, PATRIC genome ID 2038116.9), showing that homologs of HG biosynthesis genes may occasionally transfer together horizontally in mobile genetic elements.

Based on this phylogeny of *hglE*$_A$ homologs, and given that these homologs are in almost all cases encoded on a cluster of homologs of HG biosynthesis genes (Supplementary Figs. 24 and 25), we conclude that clusters of homologs of HG biosynthesis genes with yet unknown biosynthetic product were already present in (or very close in time to) the ancestor of all *Cyanobacteriia*, and that a complex

history of subsequent loss, duplication, and horizontal transfer shaped their distribution in contemporary cyanobacterial genomes.

Evolutionary reconstruction of HG biosynthesis within Cyanobacteriia

To further study the evolutionary history of HG biosynthesis, we constructed phylogenies of homologs of the seven genes (*hgdCB* and *hglE$_A$FGCA*) that are shared among most *hgl* and *hgl*-like islands (Supplementary Fig. 18), including only islands that contained homologs of all seven genes. These seven phylogenies had similar tree topologies—with *hgl* and *hgl*-like islands of heterocytous cyanobacteria each forming distinct groups, and the *hgl* islands of non-heterocytous cyanobacteria branching in between these clusters. The overall congruency between the phylogenies indicates similar evolutionary histories of genes within the vast majority of islands. We identified five heterocytous islands in which some genes clustered with the *hgl* islands and other genes clustered with the *hgl*-like islands (Supplementary Fig. 18), indicative of an incongruent evolutionary history which can be explained by the inference that part of the island originates from vertical descent and that part of the island originates from duplication and replacement—for example by homologous recombination of genes of the other island in the genome. The genes of three islands from subclade 4 and one island from subclade 9 did not cluster with the other heterocytous islands but rather with the *hgl* islands of non-heterocytous cyanobacteria, potentially indicative of horizontal transfer. In addition, even though the *hgl* islands of non-heterocytous cyanobacteria consistently clustered in between the two heterocytous island types, the position of their deep branches differed between the phylogenies, and these branches often had low bootstrap support. We attribute this to the low phylogenetic signal of the individual gene trees.

Because the seven genes have in general similar evolutionary histories, we constructed a new phylogeny of the concatenated alignment of the individual genes—excluding the five heterocytous islands with incongruent gene histories, to reconstruct a better supported evolutionary history of the *hgl* and *hgl*-like islands within *Cyanobacteriia*. We added the islands of three cyanobacteria within the heterocytous clade which have lost the ability to form heterocytes (*Raphidiopsis brookii* D9, *Raphidiopsis curvata* NIES-932, and *Cylindrospermopsis raciborskii* CENA303), and the two islands of *Cylindrospermopsis raciborskii* CYRF because its islands lack *hglT*. We moreover added the *pfa* cluster of the gammaproteobacterium *Moritella marina* ATCC 15381 as outgroup. *PfaA* is homologous and closely related to *hglE$_A$* as discussed above (see section 'Phylogeny of *hglE$_A$* homologs reveals single origin in *Cyanobacteriia*'), and the *pfa* cluster of *Moritella marina* ATCC 15381 also contains homologs of *hglFGCA* (and of *hglD*, which is not part of the seven genes used to construct this phylogeny) (Supplementary Fig. 24). As the *pfa* cluster of *Moritella marina* ATCC 15381 does not encode *hgdCB* homologs, their alignments were filled with gaps. The inclusion of a *pfa* cluster allowed us to root the tree and infer the direction of time.

In this phylogeny (Fig. 4, Supplementary Figs. 19, 20, 21a and 22), the more extended *hgl* islands with homologs of at least 10 genes discussed above and the more compact *hgl*-like islands each formed distinct groups, in line with the $hglE_A$ phylogeny (Supplementary Figs. 24 and 25). This shows that a duplication that gave rise to the two types of islands on single genomes of heterocytous cyanobacteria (*hgl* and *hgl*-like islands) predated LHeCCA. With a root placed in between the cyanobacterial islands and the *pfa* synthase of the gammaproteobacterium *Moritella marina* ATCC 15381 (Fig. 4, Supplementary Figs. 21a and 22), both island types had a similar tree topology to that of the phylogeny created using the core vertically-transferred genes (Fig. 2a)—subclades that were monophyletic in the concatenated core gene tree were also largely monophyletic in the concatenated HG biosynthesis gene tree—suggesting primarily vertical inheritance of both island types (Fig. 5). The islands of *Raphidiopsis brookii* D9, *Raphidiopsis curvata* NIES-932, and *Cylindrospermopsis raciborskii* CENA303—cyanobacteria within the heterocytous clade which have lost the ability to form heterocytes—cluster with the *hgl*-like islands (Fig. 4), indicating that they lost their more extended island and kept their *hgl*-like island even when they lost their heterocyte-forming ability, and suggesting that the *hgl*-like island is not associated with nitrogen fixation.

Even though islands from heterocytous subclade 1 branched deep within both groups (*hgl* and *hgl*-like islands), they did not branch as the most basal clade like they did in the phylogeny created using the core vertically-transferred genes (Fig. 4). Differences in branching of deeper clades between the two phylogenetic trees may be explained by the shorter alignment length of the concatenated HG biosynthesis gene tree (3,677 amino acids) compared to the concatenated core gene tree (6,933 amino acids) or by different evolutionary histories of individual genes, for example due to horizontal gene transfer after radiation of both groups. Alternatively, horizontal transfer of the complete island early in heterocytous cyanobacterial radiation can explain the observed discrepancies. The widespread presence of *hgl* islands in heterocytous cyanobacteria combined with a branching pattern that is largely consistent with that of the vertically transferred part of the genome strongly suggests that the *hgl* island was already present in LHeCCA. Additionally, even though no *hgl*-like island is present in genomes from subclades 2, 3, and 6, the deep placement of the single *hgl*-like island of subclade 1, and a close association to islands present in the sister clade of heterocytous cyanobacteria (Fig. 4), suggests that the *hgl*-like island was also present in LHeCCA.

Genomes of three heterocytous cyanobacteria from subclades 4 and one from subclade 9 contain additional *hgl* islands with a gene composition that differs from the *hgl*-like islands (Fig. 4). These islands did not cluster with the other *hgl* and *hgl*-like islands from their respective subclade. Instead, they were phylogenetically placed—together with the *hgl* islands of non-heterocytous cyanobacteria—between the two broad heterocytous phylogenetic clusters discussed above (i.e. the more extended *hgl* island and *hgl*-like islands of heterocytous cyanobacteria, whose evolutionary histories were similar to that of the concatenated core gene tree) (Fig. 4, Supplementary Fig. 21a). Even though the detailed

evolutionary history of these *hgl* islands is unclear, we assume that their phylogenetic placement suggests an origin predating LHeCCA.

The phylogenetic placement of *hgl* islands of genomes of non-heterocytous cyanobacteria in the concatenated HG biosynthesis gene tree as deeper branching sister clades and in between the two broad heterocytous phylogenetic clusters (Fig. 4, Supplementary Fig. 21a), depicts an ambiguous evolutionary history. The two broad heterocytous phylogenetic clusters form a clade with several non-heterocytous cyanobacteria, one of the two additional *hgl* islands found in the heterocytous *Chlorogloeopsis fritschii* PCC 6912, and the additional island of *Nostoc* sp. FACHB-892 (Supplementary Fig. 21a). The islands of a closely related sister clade of heterocytous cyanobacteria (Fig. 2a, Supplementary Fig. 21b) cluster closely together with *hgl*-like islands instead of with the more extended heterocytous *hgl* islands, and the *hgl* islands of the more distantly related filamentous *Moorea* species are more similar to the more extended heterocytous *hgl* islands than these sister clade islands are (Supplementary Fig. 21a). Based on this phylogeny and given that a sister clade of heterocytous cyanobacteria is also a close sister to the *hgl*-like islands in this tree and branches off after the duplication, we place the duplication that led to the two types of heterocytous islands before LHeCCA, and before the last common ancestor of the heterocytous cyanobacteria and their sister clade (Supplementary Fig. 21a). Similarly, the duplication may predate the last common ancestor of these clades and of *Moorea* spp. and *Cyanobacteria* bacterium UBA8543 (Supplementary Fig. 21a). However, because the tree topology (Supplementary Fig. 21a) of the deeper branches of the phylogeny differs from that of the phylogeny created using the core vertically-transferred genes, we also infer that horizontal transfer shaped the deeper branches of the phylogeny (Supplementary Fig. 21b). Therefore, the phylogenetic evidence is inconclusive and does not allow for the identification of the exact moment in time of the duplication that led to both types of islands in heterocytous cyanobacteria.

In summary, clusters of homologs of HG biosynthesis genes were likely already present in the last common ancestor of *Cyanobacteriia*—based on the deep branching of basal cyanobacteria in the phylogeny of *hglE$_A$* homologs followed by a complex history of subsequent loss, duplications, and horizontal transfer. In addition, a duplication predating LHeCCA led to two types of islands in heterocytous cyanobacteria.

Potential origin of HGs from ancient 1,3-diols

Two cultures of non-heterocytous cyanobacteria with an hgl island do not produce HGs

To further investigate the origin of HG biosynthesis, we screened two strains from outside the heterocytous clade that contained an *hgl* island—*Pleurocapsales* cyanobacterium LEGE 10410 (*hgl* island with homologs of 10 different genes; *hgdCB*, *all5343*, *all5344*, and *hglBACGFE$_A$*; Supplementary

Data 2) and *Gloeocapsopsis crepidinum* LEGE 06123 (*hgl* island with homologs of 10 different hits; *hgdCB*, *alr5348*, *asr5350*, and *hglE$_A$FGCAB*; Supplementary Data 2)—for the presence of HGs and HG-related compounds. *Gloeocapsopsis crepidinum* LEGE 06123 is part of a closely related sister clade of the heterocytous cyanobacteria, and *Pleurocapsales* cyanobacterium LEGE 10410 is much more distantly related (Figs. 2a and 6a, Supplementary Fig. 21b). Based on the presence of a *nif* island (Supplementary Table 7) both these non-heterocytous strains are capable of nitrogen fixation. As the *hgl* islands in these genomes likely represent a phylogenetic lineage of the island that predates LHeCCA (see section 'Evolutionary reconstruction of HG biosynthesis within *Cyanobacteriia*' above), their biosynthetic products may be 'remnants' of molecules that were produced before the emergence of the heterocyte, and thus give insights into the evolutionary acquisition of HGs for heterocyte formation.

The gene compositions of the *hgl* islands of both strains were relatively similar (Fig. 6d). Out of the 13 genes that are commonly conserved on regular *hgl* islands (see section 'Gene conservation on *hgl* islands with homologs of at least 10 HG biosynthesis genes' above), homologs of eight genes were present on the *hgl* islands of both strains (*hgdCB* and *hglE$_A$FGCAB*), and *Pleurocapsales* cyanobacterium LEGE 10410 also encoded homologs of *all5343* and *all5344*. Absent from both islands were homologs of *hglT*, *hgdA*, and *hglD*. Whereas *Pleurocapsales* cyanobacterium LEGE 10410 does not contain a high-scoring *hglT* homolog anywhere on its genome (bit-score of best hit: 137; see section '*HglT* is absent from pentose HG producing symbiotic cyanobacteria' above for a discussion of 'high-scoring' *hglT* hits), a high-scoring homolog of *hglT* is encoded close to the *hgl* island of *Gloeocapsopsis crepidinum* LEGE 06123 (6 ORFs from the *hglB* homolog, with a bit-sore of 549; Supplementary Data 2).

In culture, daughter cells of both strains remained attached to the parent cell upon division, and were surrounded by a polysaccharide layer, thus appearing as aggregates under the microscope (Supplementary Fig. 26), under all growth conditions. We did not identify HGs in any of the tested culturing conditions, neither after ageing nor in media without nitrogen (Supplementary Fig. 26) which would induce akinete and heterocyte formation (respectively) in heterocytous cyanobacteria. Based on the absence of HGs, we hypothesized that the *hgl* islands of the two strains produce compounds that resemble HGs or AGs but are structurally different, which we call 'HG analogs' from hereon. Based on the absence of *hglT*—the GT that attaches the glucose headgroup to the AG in canonical HG biosynthesis (Supplementary Fig. 1)—from the *hgl* islands of both strains, our initial targets for potential HG analogs produced by both non-heterocytous cultures were the AGs (Fig. 1a).

Identification of HG analogs in non-heterocytous cyanobacteria

As C_{26} - C_{32} alkyl diols, keto-diols etc. are more amenable to analysis by gas chromatography (GC) than by liquid chromatography (LC) (24), we first examined the lipid extracts of the biomass of *Gloeocapsopsis crepidinum* LEGE 06123 and *Pleurocapsales* cyanobacterium LEGE 10410 by gas chromatography mass spectrometry (GC-MS). Based on published spectra of acid hydrolyzed HGs (24)

we examined extracted ion chromatograms (MS^2) of *m/z* 219, a diagnostic fragment found in both silylated triols, tetrols and triol-ketos. In both strains we detected a compound of interest eluting at 29.9 min, which gave rise to a spectrum (Supplementary Fig. 27a) with a diagnostic *m/z* 219 fragment (indicative of a 1,3-diol) but lacking a *m/z* 117 fragment, diagnostic for a component with a hydroxy moiety at the ω-1 position, and lacking ions at *m/z* 58 and 130, diagnostic of a keto group at the ω-1 position (24). To further elucidate the structure of this component, we carried out re-analysis after silylation with deuterated N,O-bis(trimethylsilyl)trifluoroacetamide (BSFTA). This gave rise to a mass spectrum (Supplementary Fig. 27b) with an ion of interest that contained either 9 or 18 deuterations, indicative that the fragment ion contained either one or two silylated hydroxy (OH) groups. Of particular interest was the ion at *m/z* 397 in the non-deuterated component, which appeared at *m/z* 406 after deuterated silylation. This 9 Da increase indicates that this fragment contains one hydroxy moiety, and hence the ion at *m/z* 397 was assigned as a C_{22} with a single silylated OH group. This ion assignment, in combination with the presence of the *m/z* 219 ion indicative of a 1,3-diol and the absence of diagnostic ions for oxygen moieties at the ω-1 position allowed us to tentatively assign this component as tetracosane-1,3-diol.

Additionally, a later eluting component was detected in *Pleurocapsales* cyanobacterium LEGE 10410, eluting at 45.1 min, while two similar other late eluting components were detected in *Gloeocapsopsis crepidinum* LEGE 06123, eluting at 43.6 and 52.9 min. All three late eluting components gave rise to spectra indicative of compounds containing a silylated sugar moiety, e.g. with dominant ions at *m/z* 204 and 217 (ref. (54)) (Supplementary Fig. 28). Of note was the presence in all three spectra of an ion at *m/z* 397, providing evidence that these four components were also composed of tetracosane-1,3-diols. The sugar in *Pleurocapsales* cyanobacterium LEGE 10410 was identified (Supplementary Fig. 28a) as a hexose on comparison of its mass spectrum with library mass spectra (NIST Mass Spectral Library, Version 2.0, 2012). The two sugars in *Gloeocapsopsis crepidinum* LEGE 06123 (Supplementary Fig. 28b,c) were identified as pentose sugars, in pentopyranose form, based on spectral library matches (NIST).

To further probe into the structure of these sugar-bearing tetracosane-1,3-diols, we examined the extracts by ultra-high-performance liquid chromatography coupled to high-resolution mass spectrometry (UHPLC–HRMS). In both the lipid extracts of *Pleurocapsales* cyanobacterium LEGE 10410 and *Gloeocapsopsis crepidinum* LEGE 06123, we first screened for the presence of tetracosane-1,3-diol ($[M+H]^+$ $C_{24}H_{51}O_2$, *m/z* 371.389). This component was present in both extracts, as two isomers, eluting at 16.3 min (*Pleurocapsales* cyanobacterium LEGE 10410 and *Gloeocapsopsis crepidinum* LEGE 06123) and 19.3 min (*Pleurocapsales* cyanobacterium LEGE 10410 only), both of which gave rise to similar but not identical MS^2 spectra (Supplementary Fig. 29). In addition, we detected three larger components which gave rise to fragment ions at *m/z* 371.389, distributed across the two strains. The first of these components, present only in *Gloeocapsopsis crepidinum* LEGE 06123, eluted at 14.67 min and

gave rise to a $[M+NH_4]^+$ ion at *m/z* 652.499. Based on its accurate mass and its MS^2 spectrum (Supplementary Fig. 30a), and based on the above-mentioned sugar assignment from GCMS analysis, this was tentatively assigned as a 1-(O-dipentopyranose)-3-tetracosanol, e.g. a tetracosane-1,3-diol attached to two pentopyranose sugars. The next component eluted at 14.81 min and gave rise to a $[M+NH_4]^+$ ion at *m/z* 550.468. Based on its accurate mass and its MS^2 spectrum (Supplementary Fig. 30b) this was tentatively assigned as a 1-(O-hexose)-3-tetracosaneol, e.g. a tetracosane-1,3-diol attached to one hexose sugar, thus resembling the slightly longer H HG_{26} ol detected in several heterocytous strains (see section 'Heterocytous cyanobacteria produce a wide diversity of new HGs' above). This component was only detected in the lipid extracts of *Pleurocapsales* cyanobacterium LEGE 10410. Finally, the third component eluted at 15.22 min in both *Pleurocapsales* cyanobacterium LEGE 10410 and *Gloeocapsopsis crepidinum* LEGE 06123 and gave rise to a $[M+NH_4]^+$ ion at m/z 520.457. Based on its accurate mass and its MS^2 spectrum (Supplementary Fig. 30c) this was tentatively assigned as a 1-(O-pentopyranose)-3-tetracosaneol, e.g. a tetracosane-1,3-diol attached to one pentopyranose sugar. The distribution of the tetracosane-1,3-diols and the 1-(O-sugar)-3-tetracosaneols arising from UHPLC–HRMS analysis agreed well with the analysis by GC-MS (Supplementary Table 17). However, it should be noted that intact polar lipid species have diverse degrees of ionization efficiencies during UHPLC–HRMS and hence the peak areas, in response units, of different components do not always accurately reflect their relative abundances in the source material.

Potential production of HG analogs by the PKSs encoded by hgl islands of non-heterocytous cyanobacteria

Thus, both non-heterocytous strains produced tetracosane-1,3-diol under all tested culturing conditions, a molecule that structurally resembles an AG of canonical HG biosynthesis but with a shorter 24-carbon chain and no keto or alcohol group at the ω-1 position (Fig. 6c, structure I). In addition, sugar-bound compounds of these molecules were present in higher abundance in both cultures and under all tested conditions, representing structural analogs to canonical HGs (Fig. 6c, structures II, III, and IV). In *Pleurocapsales* cyanobacterium LEGE 10410, these HG analogs contained mainly hexose (mean 64.8% ± 6.5% standard deviation) with a low abundance of pentopyranose (6.8% ± 1.4%), and in *Gloeocapsopsis crepidinum* LEGE 06123 pentopyranose (64.7% ± 3.2%) and dipentopyranose (26.2% ± 2.9%) (Supplementary Tables 9, 10 and 18). Given the structural resemblance to AGs and HGs of the molecules identified in these two phylogenetically distant strains (see Fig. 6a), we hypothesize that their *hgl* islands encode the enzymes responsible for their biosynthesis.

For further confirmation, we screened the heterocytous cultures for the presence of the newly identified HG analogs, and found them only in a single strain, *Calothrix* sp. CCY 0018 (Fig. 6a,b), which produced tetracosane-1,3-diol (3.0% of total HGs + HG analogs) and in higher abundance 1-(O-pentopyranose)-3-tetracosaneol (15.7%; Supplementary Table 10). The genome of this strain contains, in addition to an

expected extended *hgl* island (homologs of 15 different genes) which is probably responsible for canonical HG biosynthesis, an additional *hgl* island with a gene composition that is similar to the *hgl* islands of the two non-heterocytous strains discussed above (*hgl* island with homologs of 9 different genes; *hgdCB*, *all5343*, and *hglBACGE$_A$F*; Fig. 6d). In the concatenated HG biosynthesis gene tree, this additional island is closely related to the *hgl* island of *Pleurocapsales* cyanobacterium LEGE 10410 (Fig. 4, Supplementary Figs. 21a and 22). Thus, the additional *hgl* island of *Calothrix* sp. CCY 0018 is not closely related to the more extended *hgl* islands or *hgl*-like islands of other heterocytous cyanobacteria but instead to the *hgl* islands of non-heterocytous cyanobacteria (see section '*Hgl* and *hgl*-like islands are present throughout *Cyanobacteriia*' above). The close evolutionary association between the additional *hgl* island of *Calothrix* sp. CCY 0018 and that of *Pleurocapsales* cyanobacterium LEGE 10410, in addition to the shared production of HG analogs by these strains, provides further evidence for the biosynthesis of these molecules by an ancient phylogenetic lineage of the *hgl* island. Whereas the canonical HGs of *Calothrix* sp. CCY 0018 are likely produced by the enzymes encoded by its more extended *hgl* island (containing homologs of 15 different genes), the HG analogs may thus be produced by the enzymes encoded by its additional *hgl* island (with homologs of 9 different genes).

The structural differences between the HG analogs and canonical HGs—an alkyl chain containing only 24 carbon atoms compared to at least 26 in canonical HGs and the absence of a keto or alcohol group at the ω-1 position—may derive from different enzymatic activity of the encoded PKSs. Compared to the enzymatic activity encoded by the more extended *hgl* island in heterocytous cyanobacteria (Supplementary Fig. 1), where canonical HGs result from the omission of the first dehydration and reduction steps, the first condensation round of the PKS encoded by the *hgl* islands of *Pleurocapsales* cyanobacterium LEGE 10410, *Gloeocapsopsis crepidinum* LEGE 06123, and the additional *hgl* island of *Calothrix* sp. CCY 0018 could include the reduction of the keto group to an alcohol group, its dehydration into a double bond, and its subsequent reduction to a saturated carbon chain, thus resulting in a lipid lacking the ω-1 alcohol group. In addition, a lower number of extension cycles compared to canonical HG biosynthesis would result in a shorter alkyl chain. Alternatively, the HG analogs could result from modification of a canonical AG after biosynthesis by the PKS via a yet unidentified mechanism. In both cases, the attachment of the glucose headgroup to the tetracosane-1,3-diol could be carried out by a GT encoded by the island, for example the *all5343* homologs on the *hgl* island of *Pleurocapsales* cyanobacterium LEGE 10410 and on the additional *hgl* island of *Calothrix* sp. CCY 0018, or encoded anywhere else on the genome, for example the high-scoring *hglT* hit of *Gloeocapsopsis crepidinum* LEGE 06123.

Even though the exact enzymatic activity of the encoded PKS remains unknown, we note that the *hgl* islands of both non-heterocytous strains and the additional *hgl* island of *Calothrix* sp. CCY 0018 do not encode a homolog of the ketoacyl synthase *hglD*. Moreover, the expression region may encode other genes that are unrelated to the queried *hgl* island genes of *Anabaena* sp. PCC 7120 and which are

therefore not included in our *hgl* island definition, but which may be involved in the biosynthesis of the HG analogs—including potentially carrying out condensation reactions where no functional groups are introduced or shortening of the alkyl chain.

Implications for the evolution of the heterocyte

We thus for the first time identify HG analogs in non-heterocytous cyanobacteria, which we speculate are produced by the *hgl* islands encoded by their genomes. Given that the molecules are produced by the non-heterocytous strains irrespective of nitrogen source or age of the culture, they are likely not associated with nitrogen fixation nor with (a process similar to) akinete formation. Even though the function of the HG analogs thus remains unknown, the presence of *hgdC* and *hgdB* homologs (encoding potential ABC transporter proteins) on the *hgl* islands of the strains that produce them, suggests that the HG analogs are exported across the inner membrane and cell wall. Here, they may be incorporated into a cell envelope like in heterocytes, or be released in the environment. Future investigations into the non-heterocytous *Cyanobacteriia* carrying an *hgl* island that we identified here may shed light on the functional role of these novel natural products.

If the *hgl* islands in non-heterocytous strains are indeed responsible for the production of the here identified HG analogs, the molecules may be 'remnants' of a biosynthetic process that predates LHeCCA. Neofunctionalization of the already existing *hgl* island in LHeCCA, which was previously not used for nitrogen fixation, could have enabled the formation of a new type of cell envelope. Chain length extension and/or the addition of a keto or alcohol group at the ω-1 position may have improved oxygen impermeability and enabled nitrogen fixation by the oxygen-sensitive nitrogenase enzyme in an increasingly oxygenated atmosphere. Alternatively, the here identified lipids may be different from the molecules produced by the ancestors of LHeCCA. For example, the divergent gene composition of *hgl* islands in non-heterocytous cyanobacteria today may suggest that the ancestors of LHeCCA already contained an almost 'complete' *hgl* island to produce canonical HGs, and the *hgl* islands of contemporary non-heterocytous cyanobacteria reflect differential loss of individual genes from their island. Since none of the *hgl* islands of non-heterocytous strains contain *hglT* homologs, the inclusion of this GT into the island may have been a 'late' event relatively close in time to LHeCCA. However, since very similar molecules are biosynthesized by at least three distantly related strains in *Cyanobacteriia* (*Pleurocapsales* cyanobacterium LEGE 10410, *Gloeocapsopsis crepidinum* LEGE 06123, and *Calothrix* sp. CCY 0018), a scenario involving a change in biosynthetic product would require multiple similar independent changes from the ancestral enzymatic functionality to that of the contemporary *hgl* islands in the three strains, or alternatively, multiple horizontal transfer events.

As *hgl* islands were likely already present in the last common ancestor of *Cyanobacteriia*, it is an open question whether these islands also already produced HG analogs like the more recent ancestors of LHeCCA. The common ancestor of the *hgl* islands of the cyanobacteria that we identify here to produce

HG analogs predates the duplication that led to the two types of islands in heterocytous cyanobacteria (Supplementary Fig. 21a). However, it postdates the branching off of the *hgl* islands of the basal *Cyanobacteriia* like *Gloeobacter violaceus* PCC 7421. Comprehensive high-resolution characterization of the lipids produced by non-heterocytous *Cyanobacteriia* with an *hgl* island, like *Gloeobacter violaceus* PCC 7421, and elucidation of the enzymatic potential encoded by their genomes will further our understanding of the evolution of HG analogs in *Cyanobacteriia*.

Supplementary Materials and Methods

Bacterial strains utilized or obtained during cloning and growth conditions

Escherichia coli strains NEB 5-alpha (New England Biolabs) and TOP10 (ThermoFisher Scientific) were used for plasmid manipulation, grown at 37 °C in LB (Miller) Broth (Sigma Aldrich, St. Louis, MO, USA) or in solid LB (Miller) supplemented with bacteriological agar (VWR, Solon, OH, USA). pRL443 (Addgene plasmid # 70261 ; http://n2t.net/addgene:70261; RRID:Addgene_70261) and pRL623 (Addgene plasmid # 58494 ; http://n2t.net/addgene:58494; RRID:Addgene_58494) were a gift from Peter Wolk, and pAM5404 was a gift from Susan Golden (Addgene plasmid # 132660; http://n2t.net/addgene:132660 ; RRID:Addgene_132660) obtained through Addgene (Watertown, MA, USA).

Anabaena sp. PCC 7120 *wild-type (wt)* and Δ*hglT* (4) were routinely grown at 30 °C in liquid BG11 medium or BG11 without nitrogen ($BG11_0$) (55) (see Supplementary Table 2), supplemented with appropriate antibiotics, and incubated under constant white light illumination (60-70 $\mu E/m^2/s$) with or without agitation at 120 rpm (INFORS, Bottmingen, Switzerland). Cells were also grown in solid BG11 medium supplemented with bacteriological agar (VWR, Solon, OH, USA) and the appropriate antibiotics. When required, the following antibiotic concentrations were used: ampicillin (100 µg/ml), kanamycin (20 µg/ml), spectinomycin (25 µg/ml), streptomycin (10 µg/ml), and chloramphenicol (25 µg/ml). Growth of *Anabaena* strains was monitored by measuring OD_{730} in a V-1200 Spectrophotometer (VWR International Europe, Leuven, Belgium). To induce heterocyte formation, cells were either grown directly in $BG11_0$ media or transferred from BG11 to $BG11_0$ by either: (i) washing the culture three times with $BG11_0$ (*wt* and Δ*hglT*) (ii) a minimum of five successive 1:10 culture dilutions in $BG11_0$ (ARP001 and ARP003), (iii) two to five rounds of cell collection and transfer to $BG11_0$ medium (ARP002n, ARP004-007).

Gloeocapsopsis crepidinum LEGE 06123 and *Pleurocapsales* cyanobacterium LEGE 10410 were routinely grown at 21 °C in MN and MN_0 media (see Supplementary Table 2) and incubated under a 12:12 light:dark regime, with white light illumination (30-40 $\mu E/m^2/s$) and without agitation (SANYO MLR-350, Japan). Cultures were either grown directly in MN media or transferred from MN to MN_0 using a 1:10 dilution. After three successive transfers in MN_0, cultures were incubated for 38 days and then harvested. To evaluate the effect of other stressors such as nutrient limitation and culture age, the first transfer of the cultures was also harvested after 77 days.

Biomass was harvested by centrifugation at 2500 rpm for 10 min, washed three times with bi-distilled water and stored at -20 °C until further processing.

Microscopic analysis

Prior to harvesting, strains *Gloeocapsopsis crepidinum* LEGE 06123 and *Pleurocapsales* cyanobacterium LEGE 10410 were analyzed under the microscope. To visualize the presence of external polysaccharide a fresh aliquot of each culture was incubated with an equal volume of a 0.5% solution of Alcian blue (Sigma Aldrich, Steinheim, Germany) at room temperature for 10 min, which was then visualized using bright field microscopy (Axio Imager M2, Zeiss, magnification x200 - x400). Cell lipids were visualized using Nile Red (Sigma Aldrich, Steinheim, Germany). 40 µl of culture were incubated with 50 µl DMSO 40% and 10 µl Nile Red (10 µlg/ µl in 40% DMSO) at room temperature for 5 to 15 min. Stained samples were then visualized using epifluorescence microscopy on an Axio Imager M2 microscope (Zeiss, magnification x400) coupled to a Colibri LED light source (Zeiss, 555 nm excitation wavelength).

Nucleic acids extraction and sequencing

Genomic DNA (gDNA) was extracted from strains described in Supplementary Table 3 using the DNeasy PowerSoil kit (Qiagen, Hilden, Germany) with minor modifications, namely: samples were disrupted using the bead mill homogenizer twice for 10 seconds at a speed of 3.55 m s^{-1} with a 30 second dwell. Concentration of genomic gDNA extracts was analyzed using a Nanodrop ND-1000 Spectrophotometer (NanoDrop Technologies Inc. Wilmington, DE, USA). gDNA was sequenced using Illumina NovaSeq 6000 150bp paired-end sequencing, achieving a total of 13 to 22 million reads per strain. Library preparation using the Illumina TruSeq Nano (350bp insert) library kit and sequencing were carried out by Macrogen Europe (Amsterdam, The Netherlands) (12 strains), and library preparation using Nextera XT DNA Library Preparation Kit and sequencing were carried out by Macrogen Korea (Seoul, Republic of Korea) (two strains) (Supplementary Table 3.

Genome assemblies

Adapter sequences and polyG tails were removed from the Illumina reads using Trimmomatic (v0.36; using arguments 'PE', '-phred33', and 'ILLUMINACLIP: adapters.fa:2:30:10 LEADING:20 TRAILING:20 SLIDINGWINDOW:5:20 MINLEN:40') (56) and Cutadapt (v1.16; using arguments '--nextseq-trim=20', '-m 40', '-q 20', '-n 5', '--discard-trimmed') (57), respectively. Sequence quality was checked using FastQC (v0.11.9) (58). Sequencing reads were assembled into draft genomes using BiosyntheticSPAdes (v3.14.1) (59) with default parameter settings. Quality and potential contamination of the assemblies were assessed with BlobTools2 (v2.3.3) (60), integrating the output of BLASTN (v2.10.1+) (61, 62) to the NCBI nt database downloaded on April 1st 2021, of DIAMOND blastx (v2.0.8.146) (63) to the UniProt 2021_01 database (64), of Minimap2 (v2.17-r941) (65) to map the reads back to the assembly, and of BUSCO (v5.1.1) (66) with database version odb10. Only contiguous sequences (contigs) assigned as 'Cyanobacteria', as 'Bacterial-undefined' or not assigned to any phylum ('no-hit'), were retained. To confirm the absence of contigs of other phyla in the resulting assemblies we used BlobTools2 integrating data of BLASTN, Minimap2, and BUSCO. This procedure was

repeated for four genomes (CCY 9926, CCY 0103, UAM 290 and UAM 292) where proteobacterial contigs were detected. Additionally, in two of these genomes (UAM290 and UAM292) only contigs with a minimum coverage of 3x and a GC percentage of 30-50% were retained.

Selection of publicly available cyanobacterial genomes

We downloaded all genomes from the PATRIC genome database (67) (currently part of the BV-BRC database (68)) that had a taxonomic assignment as 'phylum *Cyanobacteria*' (taxonomy ID 1117 according to NCBI taxonomy) based on the 'genome_lineage' file on the PATRIC ftp server (ftp.patricbrc.org) from April 30, 2022. As some of the genome sequences in PATRIC were identical, we identified replicates based on concatenated DNA sequences and concatenated sorted DNA sequences and kept one genome per group of replicates.

The final selection of 3,657 genomes (Supplementary Data 1) included 258 genomes with genome status 'complete', 3,021 with genome status 'WGS', 342 with genome status 'Plasmid' and 36 with unknown genome status. The genomes were taxonomically reannotated with GTDB-Tk (v2.1.0) (69) using release207_v2 of the Genome Taxonomy Database (GTDB) (70), revealing that most of the chromosomal genomes were from the class *Cyanobacteriia*. The annotation algorithm of GTDB-Tk is based on phylogenetic placement of core genome marker genes and thus does not allow for taxonomic annotation of plasmids. GTDB-Tk placed 52 and five genomes in the non-photosynthetic cyanobacterial classes *Vampirovibrionia* and *Sericytochromatia*, respectively, and 20 genomes outside cyanobacteria (Supplementary Data 1). Genome quality was assessed with CheckM (v1.1.3) (71) in the lineage-specific workflow (Supplementary Data 1). Genomes from strains with known morphology (unicellular, baeocytous, filamentous, heterocytous, and ramified) based on (7) were identified based on their taxonomy ID.

Query of HG biosynthesis genes in cyanobacterial genomes

Proteins were predicted on the PATRIC genomes and on the 14 newly sequenced genomes with Prodigal (v2.6.3) (72) in single genome mode. The 19 protein sequences that are encoded by the *hgl* island of *Anabaena* sp. PCC 7120 (see Supplementary Fig. 1) were extracted from the protein files of GCA000009705.1_ASM970v1 on GenBank (73, 74) and queried against a single concatenated protein file containing all proteins of the PATRIC genomes and of the 14 newly sequenced genomes with BLASTP (v2.12.0+) (61). No protein-coding sequences were predicted on 78 PATRIC genomes, which were all plasmids according to the PATRIC metadata table, and the final set of proteins thus encompassed 3,579 genomes. Hits with an *e*-value ≤1e-5 and query coverage ≥50% were considered homologous.

Definition of overlapping hits and of genomic clusters of HG biosynthesis and *nif* genes

Some of the queried HG biosynthesis gene from *Anabaena* sp. PCC 7120 may be fused in other genomes (or be fissions in *Anabaena* sp. PCC 7120), and thus be encoded together on single ORFs. Therefore,

we allowed for multiple hits per open reading frame (ORF) if these hits did not overlap substantially. Hits were defined as 'overlapping' if at least one of the hits had ≥50% overlap on the subject sequence with another hit. For a group of overlapping hits, the single best hit was chosen according to bit-score. 'Non-overlapping' hits—i.e. hits that had <50% overlap on the subject sequence with any other hit—where all considered present on the subject sequence.

ORFs with hits to HG biosynthesis genes were considered part of a genomic cluster if they were separated by ≤3 ORFs on the contig. For example, the first predicted ORF and the fifth predicted ORF on a contig were considered connected by this definition, but not the first and the sixth unless an ORF in between also contained a hit to an HG biosynthesis gene. An ORF was considered close to a contig edge if it was within three ORFs of the edge, for example the fourth ORF on a contig was considered close to the edge but not the fifth. '*Hgl* islands' were defined as genomic clusters containing homologs of at least seven of the queried HG biosynthesis genes (irrespective of the number of ORFs on which they are encoded or the copy number of the gene on the cluster).

Nif genes and genomic clusters of *nif* genes were defined similar to the HG biosynthesis genes and genomic clusters described above. We queried nitrogenase protein sequences encoded by *nif* genes from *Anabaena* sp. PCC 7120 of GCA000009705.1_ASM970v1 that are part of the nitrogenase gene cluster depicted in Figure 2 of ref. (9): alr1407 (*nifV1*), asr1408 (*nifZ*), asr1409 (*nifT*), all1433 (*nifW*), all1436 (*nifX*), all1437 (*nifN*), all1438 (*nifE*), all1440 (*nifK*), all1454 (*nifD*), all1455 (*nifH*), all1456 (*nifU*), all1457 (*nifS*), alr1459 (*xisF*), and all1517 (*nifB*). Overlapping and non-overlapping hits were defined and dealt with as above for the HG biosynthesis genes, as were genomic clusters of ORFs. '*Nif* islands' were defined as genomic clusters containing homologs of at least five of the queried *nif* genes (irrespective of the number of ORFs on which they are encoded or the copy number of the gene on the cluster).

Identification of glycosyltransferases

To identify glycosyl transferases, we annotated the genomes with dbCAN2, using dbCAN HMMdb (v10.0) (75) and hmmscan, with an *e*-value <1e-15 and coverage >0.35.

Cyanobacterial core gene phylogeny

We selected the 2,777 genomes that were taxonomically annotated as 'phylum *Cyanobacteria*' according to GTDB-Tk, and had at least a medium-quality draft status according to the minimum information about metagenome-assembled genome (MIMAG) criteria (76) (estimated completeness ≥50% and contamination <10%). Similar genomes were identified with dRep (v3.4.0) (77) using the fastANI algorithm and a similarity cut-off of 95% average nucleotide identity (ANI), which has been suggested as species-level boundary (78).

The representative genome of each dRep cluster was used for constructing a phylogeny based on core genes. We started with the 27 Clusters of Orthologous Gene (COG) families that showed evidence of being primarily vertically transferred in ref. (10). For identification of these genes in the genomes, fasta files from the COG 2020 database were downloaded from the NCBI ftp server (ftp.ncbi.nih.gov/pub/COG/COG2020/data/fasta/), and aligned with MAFFT (v7.505) (79) with the '--anysymbol' flag, using the L-INS-i algorithm if the COG family contained ≤800 sequences and default parameters otherwise. HMM profiles were constructed with hmmbuild and hmmpress from the HMMER package (v3.2.2) (80). The representative genomes, with proteins longer than 100.000 amino acids removed, were annotated with the COG database using hmmscan from the HMMER package and an *e*-value <1e-5. The best hit was selected per protein sequence based on *e*-value. We counted the occurrence of the COG families in each genome and selected the 24 COG families that were present in single-copy in ≥75% of the representative genomes as our final set of core genes. These were COG0016, COG0049, COG0051, COG0052, COG0064, COG0081, COG0085, COG0087, COG0090, COG0091, COG0092, COG0093, COG0094, COG0096, COG0098, COG0099, COG0100, COG0103, COG0201, COG0202, COG0532, COG0533, COG0541, and COG0552.

Genes that were present single-copy in a genome were extracted and aligned with MAFFT using the L-INS-i algorithm. The alignments were trimmed with trimAl (v1.4.rev15) (81) in gappyout mode. If a sequence contained ≥50% gaps after trimming, it was removed from the alignment. The aligned sequences were concatenated per genome, filling in gaps when a gene was absent from the alignment—i.e. when it was not present on the genome or present in multiple copies or when it was removed after trimming. We removed the 18 representative genomes whose concatenated alignment was based on <7 genes. The final concatenated alignment contained 1,260 representative genomes (representing 2,758 genomes) and 6,933 amino acids.

A phylogenetic tree was constructed with IQ-TREE (v2.1.2) (82), using 1,000 ultrafast bootstraps (83) and model selection (84) based on nuclear models. The best-fit model (LG+R10) was chosen based on the Bayesian Information Criterion (BIC). The phylogenetic tree was visualized and decorated in Interactive Tree of Life (iTOL) (85).

Visualization of *hgl* and *nif* islands on core gene phylogeny

The branches of the core gene phylogeny are representative genomes of dRep clusters that may contain multiple genomes. We decorated each branch on the core gene phylogeny with *hgl* and *nif* islands of one selected genome from the dRep cluster, and the genome that was chosen for this visualization was not always the dRep representative genome that was used to construct the core gene phylogeny. When the dRep cluster contained a genome from a culture with associated lipid data, this genome was chosen. Alternatively, the genome from the dRep cluster that had the most extended *hgl* island in terms of number of queried genes with hits on the island was chosen.

The most extended *hgl* island in terms of number queried genes with hits on the island was drawn on the phylogeny. Presence and absence of a *nif* island in a branch of the core gene phylogeny was based on the same selected genome. Drawing direction of the *hgl* islands was based on the orientation of the *hgdC* / *hgdB* pair, or if the island had no hits to both genes of the *hglG* / *hglC* pair. The oriented *hgl* islands were aligned based on their nucleotide position relative to *hgdB* (no offset) or *hglC* (an offset of 10,135 nucleotides because this is the distance between *hgdB* and *hglG* on the genome of *Anabaena* sp. PCC 7120 (BV-BRC genome identifier 103690.50)).

<u>Phylogenies of seven *hgl* island genes and of a concatenated alignment of these genes</u>

We extracted all hits of seven HG biosynthesis genes—*hgdCB* and *hglE$_A$FGCA*—from the 324 *hgl* islands that were drawn on the core gene phylogeny. Only the protein region of the BLASTP hit was extracted, and when an island contained multiple hits to a gene, we picked the best hit in terms of bit-score. 69 *hgl* islands did not contain a hit to all seven genes and these islands were discarded. The protein sequences were aligned with MAFFT using the E-INS-i algorithm, trimmed with trimAl in gappyout mode, and phylogenetic trees were constructed with IQ-TREE, using 1,000 ultrafast bootstraps and model selection based on nuclear models. The best-fit model (*hgdC*: JTTDCMut+F+R6, *hgdB*: JTTDCMut+F+R6, *hglE$_A$*: JTTDCMut+F+R7, *hglF*: JTTDCMut+F+R5, *hglG*: JTTDCMut+F+R7, *hglC*: JTTDCMut+F+R6, *hglA*: JTTDCMut+R6) was chosen based on the BIC.

We also constructed a phylogeny based on a concatenation of these homologs of seven HG biosynthesis genes. The homologs of five heterocytous islands were excluded because their placements in the individual gene trees were incongruent, and we incorporated the hits from the *hgl* islands of four cyanobacteria that were not drawn on the core gene phylogeny. These cyanobacteria were chosen because they are non-diazotrophic cyanobacteria from within the heterocytous clade (*Raphidiopsis curvata* NIES-932, *Cylindrospermopsis raciborskii* CENA303, and *Raphidiopsis brookii* D9) or because they possess an *hgl* island but without *hglT* homologs (*Cylindrospermopsis raciborskii* CYRF; the highest-scoring *hglT* hit anywhere on its genome has a bit-score of 68.6). For these four genomes, we included *hgl* islands with hits to at least six of the queried genes. In addition, we added the *pfa* synthase of the gammaproteobacterium *Moritella marina* ATCC 15381 as outgroup which encodes *hglE$_A$FGCA* homologs but no homologs of *hgdCB*. The sequences were individually aligned and trimmed as described above, and the aligned sequences were concatenated—filling in gaps when a gene was absent from the alignment (for example for *hgdCB* in *Moritella marina* ATCC 15381). A phylogenetic tree was constructed with IQ-TREE as described above, and the best-fit model (JTTDCMut+F+R8) was chosen based on the BIC.

Phylogenetic trees were visualized and decorated in iTOL.

Query of HG biosynthesis genes throughout prokaryotes

We queried the 19 *hgl* island protein sequences of *Anabaena* sp. PCC 7120 in 225,388 prokaryotic genomes from the PATRIC genome database that had an estimated completeness ≥50% and contamination <10% based on the 'genome_summary' file on the PATRIC ftp server from January 20, 2023. Taxonomy of the genomes (encompassing 182 prokaryotic phyla) was based on the 'genome_lineage' file on the PATRIC ftp server.

Proteins were predicted in the 225,388 genomes with Prodigal. The genome protein files were individually queried, and hits with an e-value ≤1e-5 and query coverage ≥50% were considered homologous. Genomic clusters were defined as described above for the cyanobacterial genomes.

Taxonomic annotation of Beggiatoa sp. 4572_84 genome and contigs

The genome of *Beggiatoa* sp. 4572_84 (PATRIC genome ID 1972449.3) and its individual contigs were taxonomically reannotated with Bin Annotation Tool (BAT) and Contig Annotation Tool (CAT), respectively, from the CAT pack software suite (v6.0.1) (52), using the NCBI nr reference database (86) downloaded on April 22, 2024, and default parameters. Protein were predicted with Prodigal (v2.6.3) in metagenomic mode (87), and aligned to the reference database with DIAMOND (v2.0.6) (88) with the --top parameter set to 11 for CAT and to 6 for BAT.

Extended phylogeny of *hglE*$_A$ homologs

A second *hglE*$_A$ phylogeny was constructed including a larger set of sequences. We included the *hglE*$_A$ homologs that were included in the phylogeny of a concatenated alignment of seven HG biosynthesis genes (see above), the *hglE*$_A$ homologs on *hgl* islands that were not included in this phylogeny but were drawn on the cyanobacterial core gene phylogeny, and all *hglE*$_A$ hits that had a bit-score ≥1,150 of the genomes that were selected to be drawn on the core gene phylogeny from the dRep cluster (see above). The latter group includes cyanobacterial hits on additional *hgl* islands that do not encode homologs of all seven HG biosynthesis genes—and are thus not drawn on the core gene phylogeny and not included in the phylogeny based on seven HG biosynthesis genes—and cyanobacterial hits that are not present on an *hgl* island. We moreover added non-cyanobacterial *hglE*$_A$ hits that had a bit-score ≥1,150. Only hits from a single genome per non-cyanobacterial species (according to the PATRIC taxonomic annotation) were included, and hits from all genomes whose species name ended with 'bacterium' or 'sp.'—as the species affiliation of these genomes is undefined.

Only the protein region of the BLASTP hit was extracted, and when a genomic cluster contained multiple hits to *hglE*$_A$, we picked the best hit in terms of bit-score. The protein sequences were aligned with MAFFT using the E-INS-i algorithm, trimmed with trimAl in gappyout mode, and phylogenetic trees were constructed with IQ-TREE, using 1,000 ultrafast bootstraps and model selection based on nuclear models. The best-fit model (LG+F+R8) was chosen based on the BIC.

Cloning strategy and recombinant plasmid construction

Prior to further genetic modifications we confirmed the substitution of *hglT* for the *npt* kanamycin resistance cassette in the Δ*hglT Anabaena* strain via PCR using two different primer sets (Supplementary Fig. 15, Supplementary Table 11). The presence of a kanamycin resistance cassette in place of *hglT* was analyzed using primers Fw3_Hali14 and Rv2_Hali14_mod (4), which produced the expected 990 bp fragment. Whilst deletion of *hglT* in all genome copies of the Δ*hglT Anabaena* mutant strain was determined using primers HR1_hglT_F and HR2_hglT_R, if *hglT* was present a ~1700 bp band would be seen, whist if *hglT* had been successfully replaced by *npt* we would observe a ~2000 bp band (Supplementary Fig. 15). Our results confirmed that *hglT* had been successfully deleted in all genome copies. However, in addition to the expected ~2000 bp band we also observed a smaller (~550 bp) band, which might be caused by the partial loss of the *npt* kanamycin cassette (~800 bp) after kanamycin stepdown upon receival of the strain.

The PCRs described above were carried out using 1 µl gDNA extracted as described in the Material and Methods in a 25 µl polymerase chain reaction (PCR) containing, 10 µl PCR water, 5 µl Q solution, 2.5 µl Qiagen Buffer, 2 µl dNTPs (0.25 mM), 1.5 µl $MgCl_2$, 1.25 µl forward primer (4 mM), 1.25 µl reverse primer (4 mM), 0.25 µl BSA (20 mg/mL) and 0.125 µl Qiagen *Taq* polymerase. The conditions for the reaction were as follows: a 10’ initial denaturation step at 95 °C was followed by 1’ at 95 °C, 1’ at 55 °C (Fw3_Hali14 and Rv2_Hali14_mod) or 58 °C (HR1_hglT_F and HR2_hglT_R), 2’ at 72 °C, these steps were repeated 30 times, and were followed by a final extension step of 10’ at 72 °C.

The following genes of interest (GOIs) were selected from the *Richelia intracellularis* HH01 genome (taxon ID 2579778779) and their sequence obtained by using The Integrated Microbial Genomes & Microbiomes system (hereafter IMG/M) (89): RINTHH_5560, RINTHH_5570, RINTHH_17770 and RINTHH_20790. Because RINTHH_5560 and RINTHH_5570 are closely located on the genome, a construct containing both genes and their intergenic region (42bp) (RINTHH_5560_5570) was also included. Additionally, *R. intracellularis* HH01 genome was downloaded and annotated using RAST (90). From this annotation an open reading frame (ORF) annotated as a glycosyltransferase that was highly similar to RINTHH_20790, albeit shorter (39 bp at the 5’) was selected. All the aforementioned genes were synthesized by Baseclear BV (Leiden, the Netherlands) under the control of P_{glnA}, a constitutive promoter active in heterocyte and vegetative cells (91).

The streptomycin/spectinomycin resistance cassette *aadA* (92), used as selective marker, was also synthesized by Baseclear B.V. and inserted into pBluescript II KS (+). All constructs were flanked by the appropriate GC-adaptors in order to make them compatible for cloning using the CYANO-VECTOR approach (93). The GOIs and the resistance cassette were inserted in pAM5404 (92), an RSF1010-based plasmid containing a mutation in *mobA*Y25F to improve cloning efficiency and an additional RK2-*bom*

site to increase its mobilization efficiency (92), as described in (93). In brief, plasmid and inserts were digested using *ZraI I* (New England Biolabs) according to the manufacturer's instructions. Fragments containing the GOIs were purified using a PCR purification kit (QIAquick PCR Purification kit, Qiagen, Hilden, Germany). To remove potential undigested plasmid containing *aadA* in the assembly reaction, the restriction digest of pRP002 was run on a 1% agarose gel, and the appropriate fragment (1279 bp) was cut and purified using QIAquick gel extraction kit (Qiagen, Hilden, Germany).

Final constructs were assembled using NEBuilder HiFi DNA Assembly Cloning Kit (New England Biolabs) according to the manufacturer's instructions, transformed into NEB 5-alpha chemically competent *E. coli* cells and plated on LB agar plates containing spectinomycin and streptomycin. The resulting plasmids were extracted using QIAprep Spin Miniprep Kit (Qiagen, Hilden, Germany) and the presence of the correct insert was confirmed by Sanger sequencing (Baseclear B.V, Leiden, The Netherlands) using the primers PaadA_out, PglnA_F and GOI_R (Supplementary Table 11). In order to make them suitable for tri-parental mating, plasmids (hereafter referred to as cargo plasmids, Supplementary Fig. 17, Supplementary Table 12) were then subcloned in TOP10 cells (Invitrogen, Carlsbad, CA, USA). All genomic and plasmid maps generated and used in this study can be found in Supplementary Data 6.

Recombinant plasmid transfer via triparental mating

Genetic modification by triparental mating of *Anabaena* sp. PCC 7120 Δ*hglT* was carried out as described in (94, 95) with a few modifications. As helper strain, we used an *E.coli* strain containing the helper plasmid pRL623 and the conjugal plasmid pRL443 (96), hereafter referred to as helper strain. This strain was generated by biparental mating (see below) of an *E.coli* DH5alphaMCR strain containing pRL623 and a DH5alpha strain containing pRL443. *E.coli* cultures of the helper strain and TOP10 cells strains carrying the cargo plasmids were grown overnight at 37 °C, shaking at 225 rpm (Innova 43, New Brunswick Scientific), diluted 1:20 in LB without antibiotics and then grown for 2.5h at 37 °C shaking at 200 rpm in an Erlenmeyer flask of volume 10 times larger than the volume of the culture. Cells were then harvested by centrifugation for 5 min at 2500 rpm and resuspended gently in 1 mL LB per every 10 mL of initial culture. Cargo and helper plasmid were then mixed in a 1:1 ratio in a total volume of 2 mL, harvested by centrifugation for 5 min at 2500 rpm, resuspended in 100 µl LB and incubated at 30 °C for 1 h without agitation. For each mating we harvested 1.8-3.5 mL of an *Anabaena* sp. PCC 7120 Δ*hglT* (OD_{730} 0.25-0.5) culture grown in BG11 media plus kanamycin by centrifugation for 5 min at 2500 rpm and resuspended it in 100 µl BG11. *Anabaena* cell concentrates were then mixed with the biparental mating of the *E.coli* strains containing the cargo and helper plasmids, harvested by centrifugation and resuspended in 30 µl BG11. Upon resuspension, cells were plated onto a Supor Membrane Disc Filter (Pall Laboratories) on BG11 agar plates supplemented with 5% LB. The filters were allowed to dry before incubation at 30°C under light dimmed by covering the plates with a tissue paper. After approximately 24h, the filters were washed with BG11 media (Supplementary Table 2) and

the resuspended mating's were plated onto BG11 agar plates supplemented with kanamycin, streptomycin and spectinomycin. Upon appearance of *Anabaena* mutant colonies, single colonies were first replated in BG11 agar plates supplemented with antibiotics and then transferred to liquid BG11 media also with appropriate antibiotics. See Supplementary Table 13 for a summary of all the resulting *Anabaena* strains.

To check that the desired plasmids had been acquired by the host strain, plasmids were extracted using QIAprep Spin Miniprep Kit (Qiagen, Hilden, Germany) following the manufacturer's instructions with minor modifications, namely: cells from liquid cultures were harvested by centrifugation, resuspended in 500 µl P1 and disrupted as previously described (see Materials and Methods). Upon centrifugation the supernatant was processed as described in the manufacturer's protocol. Then the GOI was amplified via 50 µl polymerase chain reaction (PCR) containing, 21 µl PCR water, 10 µl Q solution, 5 µl Qiagen Buffer, 4 µl dNTPs (0.25 mM), 3 µl $MgCl_2$, 2.5 µl PaadA_out (0.2 mM), 2.5 µl GOI_R (0.2 mM), 0.5 µl BSA (20 mg/mL), 0.25 µl Qiagen *Taq* polymerase and 1 µl plasmid DNA. The conditions for the PCR reaction were as follows: a 10' initial denaturation step at 95 °C was followed by 10 cycles of 1' at 95 °C, 1' at 68.4 °C, 2' at 72 °C, 20 cycles of 1' at 95 °C, 1' at 67 °C, 2' at 72 °C followed by a final extension step of 10' at 72°C.

To verify the sequence of the inserted GOIs in the mutant strains, the PCR products of five identical reactions for each strain were pooled and analyzed on a 1% agarose gel. The resulting bands were then purified and concentrated using QIAquick gel extraction kit (Qiagen, Hilden, Germany). The sequences of the concentrated PCR products were confirmed via Sanger sequencing (Baseclear B.V, Leiden, The Netherlands) using the primers PaadA_out, PglnA_F and GOI_R (Supplementary Table 11).

Lipid extraction and analysis

Extraction of intact polar lipids (IPL) from freeze dried biomass was carried out using a modified Bligh Dyer (BD) extraction as described in (97). A known amount of deuterated diacylglyceryltrimethylhomoserine (DGTS D-9, Avanti Polar Lipids, USA) dissolved in dichloromethane (DCM) : methanol (MeOH) (1:9, v:v) was added to the extracts as internal standard and then filtered through a true regenerated cellulose 4 mm syringe filter (0.4 µM, BGB, USA). Filtered extracts were analyzed on an Agilent 1290 Infinity I ultra-high performance liquid chromatographer (UHPLC) with a thermostatted auto-injector, coupled to a Q Exactive Orbitrap MS with an Ion Max source and heated electrospray ionization probe (HESI; ThermoFisher Scientific, Waltham, MA) according to (98) (modified from (26)). Briefly, chromatographic separation was achieved with an Acquity BEH C18 column (2.1 × 150 mm, 1.7 µm, Waters), with A) MeOH:H_2O:formic acid: (14.8M) NH_3aq (85:15: 0.12:0.04 [v:v]) and B) IPA:MeOH:formic acid:(14.8 M) NH_3aq (50:50:0.12:0.04 [v:v]) at a flow rate of 0.2 mL min^{-1}. Compounds were eluted with 5% B for 3 min, followed by a linear gradient to 40% B at 12 min ending at 100% B at 50 min. Lipids were detected using positive ion

monitoring of *m/z* 350–2000 (resolution 70,000 ppm at *m/z* 200), followed by data dependent MS^2 (isolation window 1 m/z; resolution 17,500 ppm at *m/z* 200) of the 10 most abundant ions. HGs were identified using a targeted approach, each sample was screened using the combined molecular mass of the protonated ($[M+H]^+$), ammoniated ($[M+NH_4]^+$) and sodiated ($[M+Na]^+$) adducts and were identified by comparison with published MS^2 spectra (known HGs) and based on theoretical fragmentation for the novel HGs and AGs (extrapolated from the fragmentation of closely related HGs) (Supplementary Table 8).

In order to screen for aglycone-like components in the two LEGE strains, their BD extracts were also analyzed by GC-MS after methylation and silyilation. Methylation was carried out using diazomethane (CH_2N_2), then extracts were dried under N_2, cleaned over a small silica gel column (pore size 60 Å, 0.063-0.2mm, 70-230 mesh, Merck), eluted with three times the column volume of ethyl acetate (EtAc) and dried under N_2. Silylation was caried out by dissolving the methylated extract in and pyridine (10 µL) and allowed to react with N,O-Bis(trimethylsilyl)trifluoroacetamide (BSTFA, Regis Technologies Inc., IL, USA) at 60 °C for 40 min. Samples were then diluted in EtAc and analyzed using a gas chromatographer (Agilent 7990B GC) coupled to a mass spectrometer (Agilent 5977A MSD; GC-MS) equipped with a fused silica capillary column (Agilent CP Sil-5, 25 m x 0.32 mm x 0.12 µm). The temperature program was as follows: start at 70 °C, increased to 130 °C at 20 °C min^{-1}, increased to 320 °C at 4 °C min^{-1}, held at 320 °C for 25 min. Flow was held constant at 2 mL min^{-1}. For further analysis, some extracts were de-silylated by washing five times in DCM (500 µL) and drying under N_2, and then re-silylated by the method above, but with addition of deuterated BSTFA (5 µL) before re-analysis.

HG abundance as percentage of the total lipids (Supplementary Table 10) was estimated based on the sum of the peak areas of all HGs in relation to the total base peak chromatogram using the following formula:

$$\text{HGs and HG-like compounds as \% of total lipids} = \frac{\sum HGs\ and\ HG\text{-}like\ compounds\ areas}{Total\ base\ peak\ area - Internal\ standard\ area}$$

It should be noted that lipid species have diverse degrees of ionization efficiencies and hence the peak areas, in response units, of different components do not necessarily reflect their actual relative abundance. However, we chose this method because it allows for comparison between samples when analyzed together. All lipid datafiles obtained in this study can be found in Supplementary Data 5.

Supplementary Figures

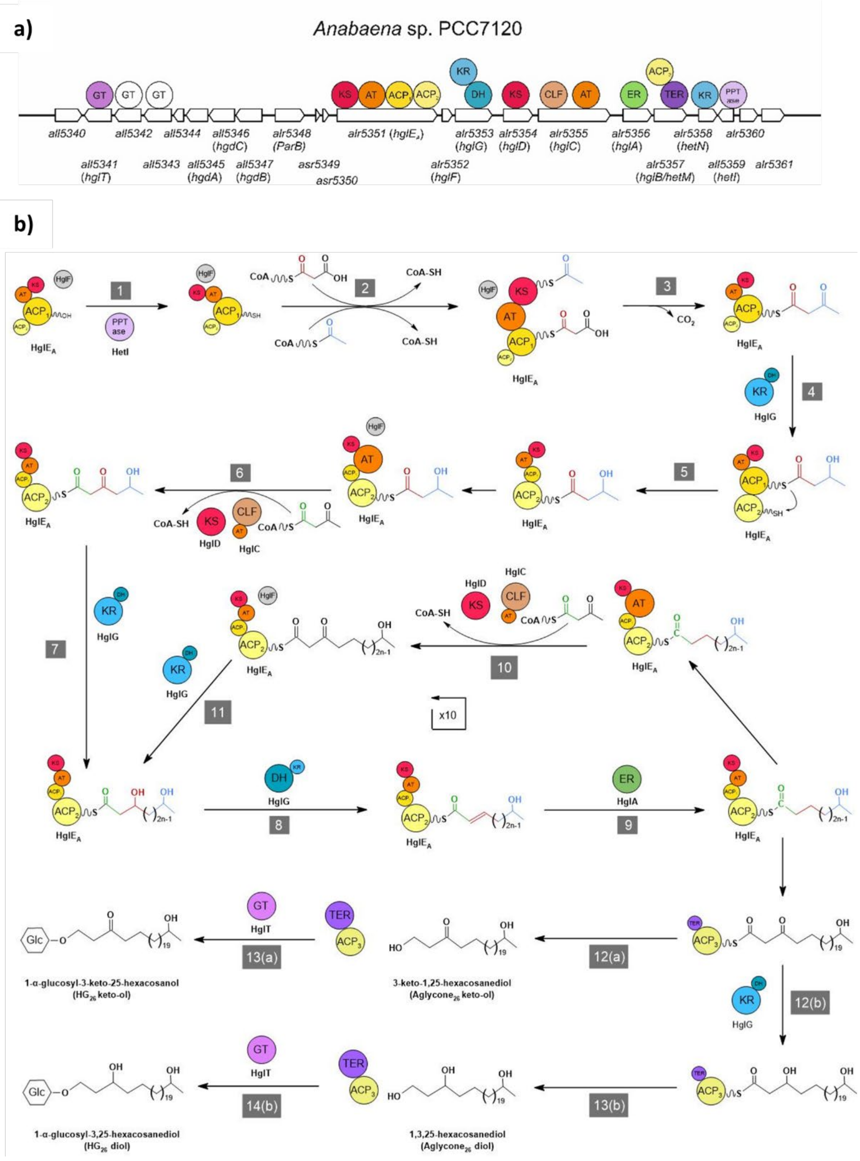

Supplementary Fig. 1. Schematic representation of *Anabaena* sp. PCC7 7120 *hgl* island and the catalytic domains present in each gene (a) and representation of the proposed HG biosynthesis pathway carried out by the encoded proteins (b) (adapted from ref. (99)). (1) Activation of ACP domain in HglE$_A$. (2) HglE$_A$ acyl transferase (AT) adds malonyl group to ACP$_1$ domain and β-ketoacyl synthase (KS) transfers acetyl residue to activated malonyl group. (3) Condensation catalyzed by HglE$_A$ KS domain. (4) Reduction of ketone by HglG KR domain. (5) Chain translocation to the second ACP domain of HglE$_A$. (6) Chain length checked by CLF domain of HglC and chain elongation catalyzed by HglE$_A$ AT and HglD (KS). (7) Ketoreduction catalyzed by HglG KR domain. (8) Dehydration catalyzed by HglG DH domain. (9) Enoyl reduction catalyzed by HglA. (10) Chain elongation catalyzed by HglE$_A$ AT and HglD (KS). Chain length checked by CLF domain of HglC. (11) Ketoreduction catalyzed by HglG KR domain. (12a) Chain termination catalyzed by HglB C-terminal thioester reductase (TER). (13a) Glucose moiety transferred to the aglycone by glycosyltransferase HglT. (12b) Ketoreduction catalyzed by HglG KR domain. (13b) Chain termination catalyzed by HglB C-terminal thioester reductase (TER). (14b) Glucose moiety transferred to the aglycone by glycosyltransferase HglT. GT, glycosyl transferase; KS, ketoacyl synthase; AT, acyl transferase; ACP, acyl carrier protein; KR, ketoreductase; DH, dehydratase; CLF, chain length factor; ER, enoyl reductase; TER, thioester reductase; PPTase, phosphopantetheinyltransferase.

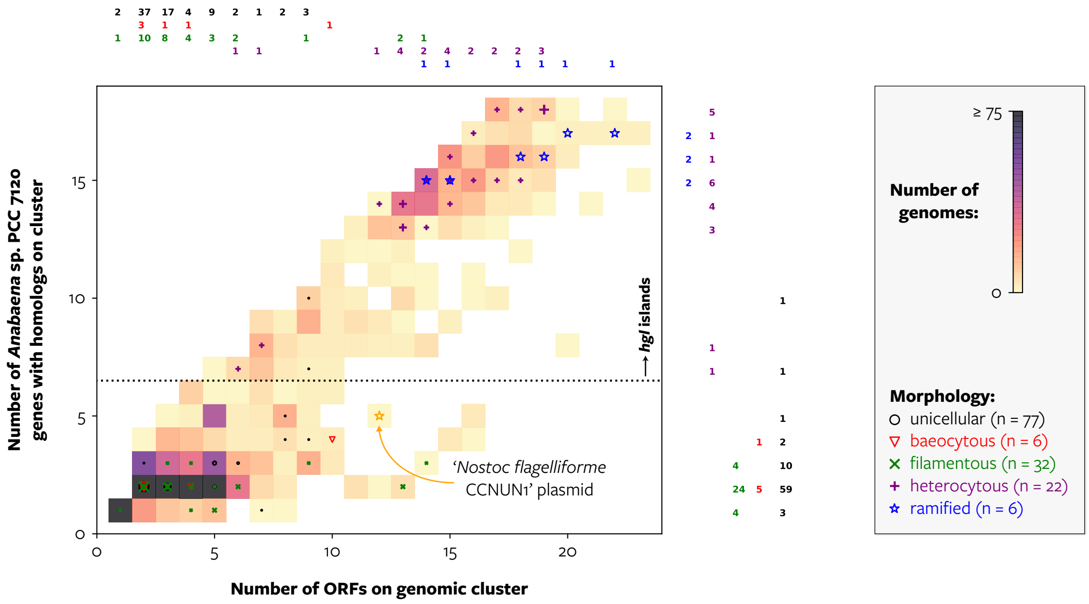

Supplementary Fig. 2. The most extended genomic cluster of homologs of HG biosynthesis genes in genomes from the PATRIC genome database (100) and in 14 newly sequenced genomes. 3,579 genomes with the taxonomic assignment 'phylum *Cyanobacteria'* in the PATRIC 'genome_lineage' file and 14 genomes of heterocytous cultures were queried. The 3,339 genomes with ≥1 hit to an *Anabaena* sp. PCC 7120 HG biosynthesis gene are drawn in the plot, and only the most extended genomic cluster (in terms of number of *Anabaena* sp. PCC 7120 genes with homologs on the cluster) is shown per genome. X-axis represents the total length of the genomic cluster as number of open reading frames (ORFs), y-axis indicates the number of *Anabaena* sp. PCC 7120 genes with homologs on the cluster. The color of the squares indicates the number of genomes represented by that square. Colored numbers on the right and top of the graph indicate the number of genomes from ref. (7) present in each row and column, respectively, divided according to their morphology. Symbols within the square depict the same, where symbol size represents genome count. The orange star indicates a plasmid of strain *Nostoc flagelliforme* CCNUN1 (see Results and Discussion for details).

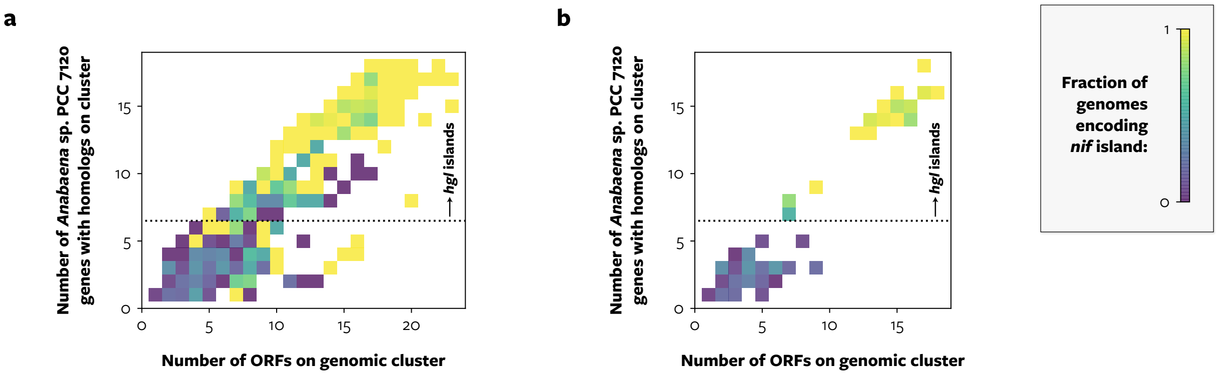

Supplementary Fig. 3. Genomic co-occurrence of genomic clusters of homologs of HG biosynthesis genes and genomic clusters of *nif* genes. a, The same plot as Supplementary Fig. 2 but with the squares colored according to the fraction of genomes in that square that encode a *nif* island (a genomic cluster containing homologs of ≥5 of the queried *nif* genes). **b**, The same plot as panel a but with only the squares that represent more than 10 genomes shown. Panel b represents 3,024 genomes, or 91% of the genomes depicted in panel a. ORF, open reading frame.

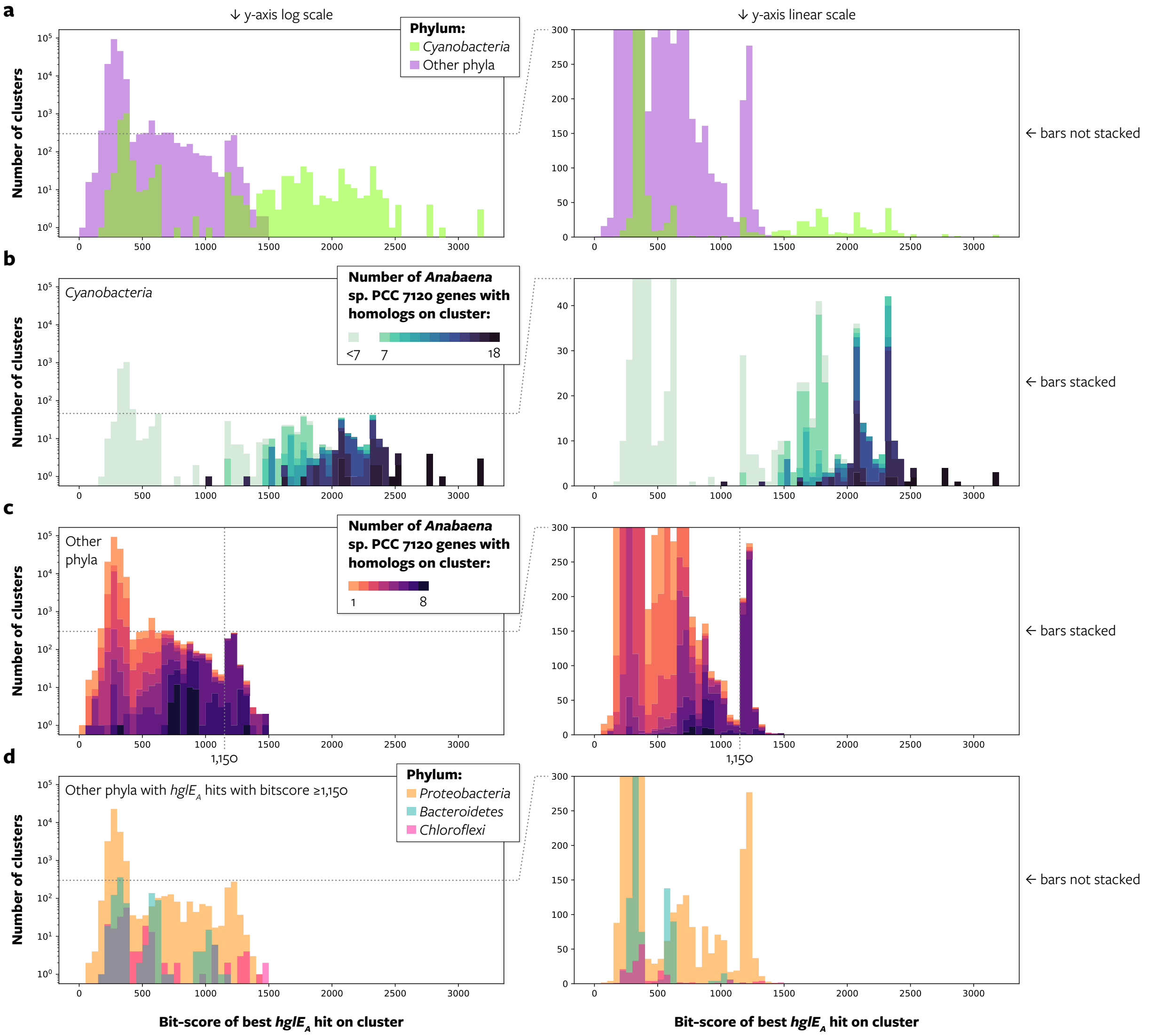

e Cyanobacterial genera with *hglE*$_A$ hits with bit-score ≥1,150 and <1,450

	Genome count:
Cyanobacteria	
Microcystis	27
Nostoc	9
Gloeobacter	3
Trichormus	2
Fischerella	2
[no genus annotation]	2
Thermosynechococcus	2
Trichodesmium	2
Calothrix	1
Cyanothece	1
Moorena	1
Scytonema	1

f Other bacterial genera with *hglE*$_A$ hits with bit-score ≥1,150

	Genome count:		Genome count:
Proteobacteria		***Bacteroidetes***	
Vibrio	352	*Dokdonia*	1
Shewanella	101		
Moritella	21		
Colwellia	14	***Chloroflexi***	
no genus annotation	14	no genus annotation	10
Psychromonas	11	*Candidatus* Chloroploca	1
Pseudoalteromonas	4	*Leptolinea*	1
Photobacterium	3		
Beggiatoa	1		
Desulfofaba	1		
Parashewanella	1		

Supplementary Fig. 4. Clusters of homologs of HG biosynthesis genes throughout the tree of life. (Caption on next page).

Supplementary Fig. 4. Clusters of homologs of HG biosynthesis genes throughout the tree of life. a, Clusters identified in genomes from the PATRIC genome database (now part of the BV-BRC database), divided into hits from cyanobacterial and non-cyanobacterial genomes. The taxonomic annotations of the PATRIC genome_lineage file were used. 225,388 genomes were screened representing 182 prokaryotic phyla. The clusters are evaluated in the context of the best *hglE*$_A$ homolog (x-axis represents the alignment score to the queried *hglE*$_A$ protein), and clusters that do not encode *hglE*$_A$ homologs are not included in this figure. **b**, Cyanobacterial clusters colored according to the number of *Anabaena* sp. PCC 7120 genes with homologs on the cluster. Gene cluster containing homologs of at least seven of the 19 queried genes are considered '*hgl* islands' in this study. **c**, Non-cyanobacterial clusters colored according to the number of *Anabaena* sp. PCC 7120 genes with homologs on the cluster. **d**, Taxonomic affiliation of non-cyanobacterial clusters that contain *hglE*$_A$ homologs with a bit-score ≥1,150. **e**, Cyanobacterial genera with clusters of homologs of HG biosynthesis genes that contain *hglE*$_A$ hits with a bit-score ≥1,150 and <1,450. **f**, Non-cyanobacterial genera with clusters of homologs of HG biosynthesis genes that contain *hglE*$_A$ hits with a bit-score ≥1,150.

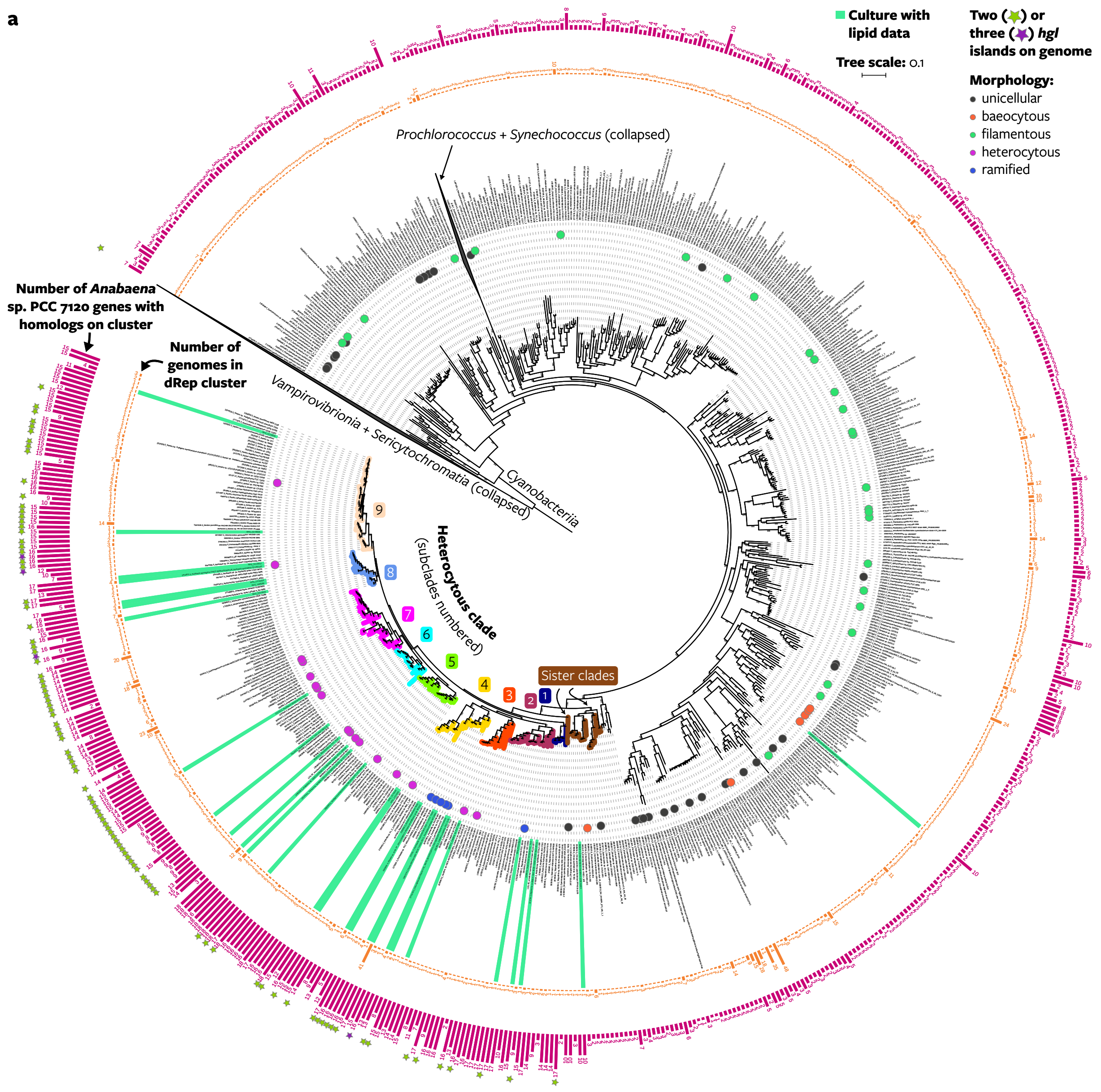

b Presence of different genera in defined clades

Sister clades	Genome count:
Chroococcidiopsis	6
no genus annotation	5*
Gloeocapsopsis	3
Aliterella	2
Gloeocapsa	2
Chlorogloea	1
Chroococcus	1
Chroogloeocystis	1
Cyanosarcina	1
Nostocaceae	1
Scytonema	1
Synechocystis	1

Subclade 2	Genome count:
Scytonema	9
Brasilonema	8
no genus annotation	4
Aetokthonos	3
Tolypothrix	2
Iningainema	1
Iphinoe	1
Mastigocladopsis	1
Stigonema	1

Subclade 4	Genome count:
Fischerella	49
Richelia	16
Chlorogloeopsis	4
Rivularia	3
Calothrix	2
Hapalosiphon	2
Mastigocladus	2
no genus annotation	1
Pelatocladus	1
Plectonema	1
Westiellopsis	1

Subclade 6	Genome count:
Calothrix	10
Nostoc	5
Tolypothrix	5
Aulosira	2
Fremyella	1
Goleter	1
Microchaete	1
Mojavia	1
no genus annotation	1

Subclade 8	Genome count:
Nodularia	16
Nostoc	4
no genus annotation	4
Anabaenopsis	1
Trichormus	1

Subclade 1	
Tolypothrix	5
Scytonema	2
Cyanomargarita	1
Hassallia	1
Spirirestis	1

Subclade 3	
Calothrix	14
Rivularia	1
Scytonematopsis	1

Subclade 5	
Nostoc	24
Anabaena	15
Trichormus	11
Anabaenopsis	1
Aulosira	1
Fortiea	1

Subclade 7	
Dolichospermum	33
Cylindrospermopsis	30
Anabaena	26
Aphanizomenon	22
Sphaerospermopsis	9
no genus annotation	7
Cylindrospermum	3
Trichormus	2
Richelia	2
Raphidiopsis	2
Cuspidothrix	2
Nostoc	1

Subclade 9	
Nostoc	74
Desmonostoc	4
Komarekiella	2
no genus annotation	1
Roholtiella	1

Supplementary Fig. 5. Annotated tree of a concatenated alignment of core vertically-transferred genes. (Caption on next page).

Supplementary Fig. 5. Annotated tree of a concatenated alignment of core vertically-transferred genes. a, Maximum likelihood cyanobacterial phylogeny created using a concatenated alignment of 24 core vertically-transferred genes (10) of 1,260 genomes representing species groups (2,758 genomes of cultured and uncultured cyanobacteria clustered at ≥95% average nucleotide identity). The tree structure is identical to Fig. 2a, but note that it is mirrored along the vertical axis. The strain name of the dRep representative genome is indicated on each leaf, and in case a different genome was used for the visualization of *hgl* islands it is indicated in between '///'. Ultrafast bootstrap approximation values are indicated along the branches of the tree. Scale bar represents the mean number of substitutions per site. Source files in Supplementary Data 4. **b**, Distribution of genera within the different heterocytous subclades and sister clades. The genus names are based on the taxonomic annotation in the 'genome_lineage' file on the PATRIC ftp server. The sister clades consist of two clades, one of which contains only a single genome (the MAG *Nostocaceae* cyanobacterium MGR_bin409) and is indicated with an asterisk.

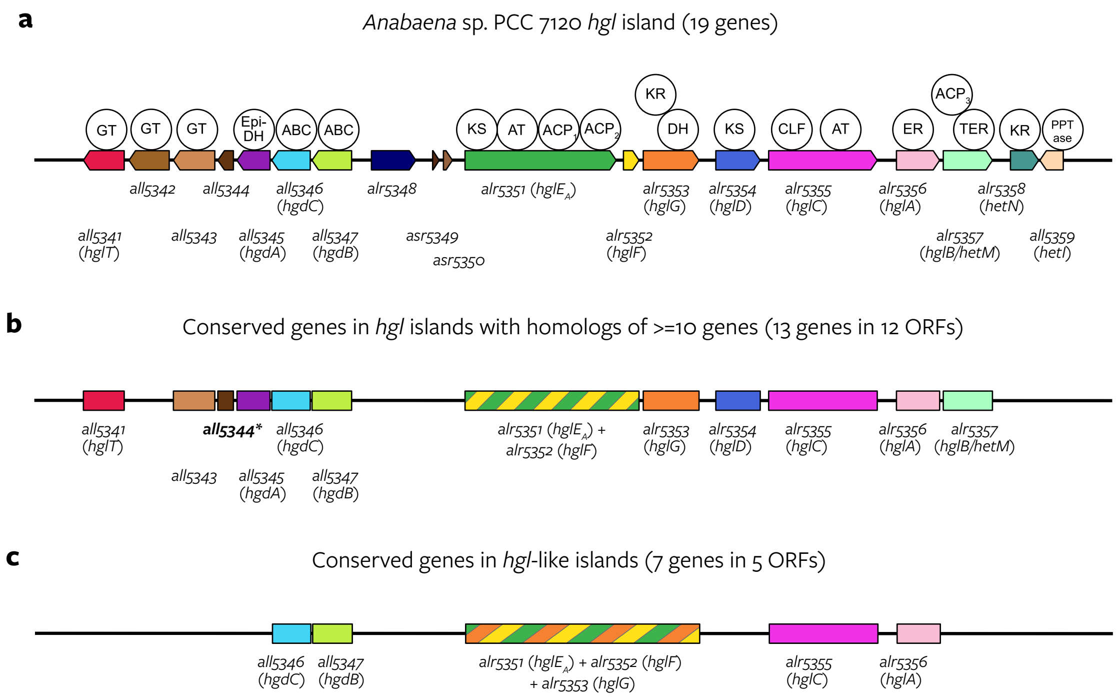

Supplementary Fig. 6. Structures of genomic clusters of (homologs of) HG biosynthesis genes. a, Gene cluster in *Anabaena* sp. PCC 7120 containing 19 genes (the *hgl* island). The known functions of the *Anabaena* sp. PCC 7120 *hgl* island genes are shown in Supplementary Table 4, and the complete HG biosynthetic pathway is depicted in Supplementary Fig. 1. Catalytic domains (circles) are plotted on top. **b,** Genes conserved in *hgl* islands containing homologs of more than 10 genes, usually consisting of 12 ORFs homologous to 13 genes of the *Anabaena* sp. PCC 7120 *hgl* island**.** All genes in this panel, except for *all5344* (* and bold), have been shown to be essential for HG biosynthesis and deposition (Supplementary Table 4). **c,** Genes conserved in *hgl*-like islands usually consisting of five ORFs homologous to seven genes of the *Anabaena* sp. PCC 7120 *hgl* island. Numbered genes depict the genomic naming scheme of *Anabaena* sp. PCC 7120, gene names assigned in previous publications are depicted within brackets. GT, glycosyl transferase; Epi-DH, epimerase-dehydratase; ABC, ATP-binding cassette (ABC) transporter; KS, ketoacyl synthase; AT, acyl transferase; ACP, acyl carrier protein; KR, ketoreductase; DH, dehydratase; CLF, chain length factor; ER, enoyl reductase; TER, thioester reductase; PPTase, phosphopantetheinyltransferase.

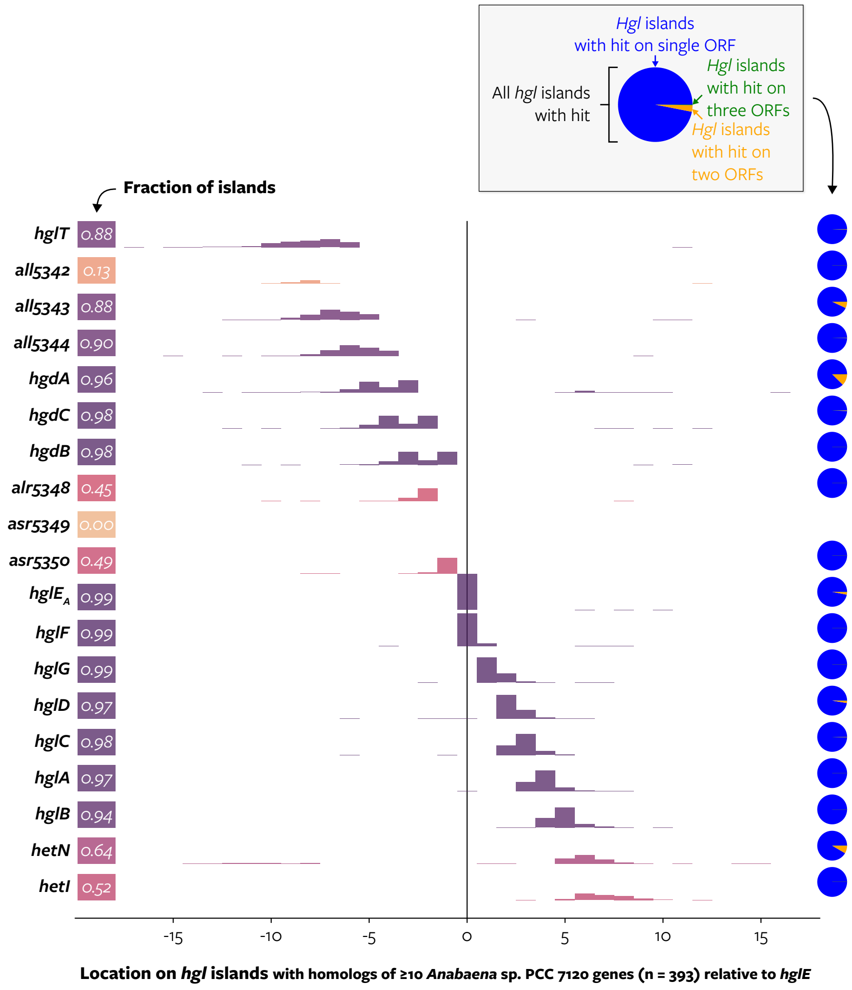

Supplementary Fig. 7. Conservation of gene location on *hgl* islands with homologs of ≥10 *Anabaena* sp. PCC 7120 HG biosynthesis genes. Histograms show the distribution of the location of hits for each queried gene. Location is measured as the distance in number of open reading frames (ORFs) between the hit and the ORF that contains an *hglE$_A$* hit, with orientation based on the *hglE$_A$* / *hglG* pair, or on the *hglT* / *hglE$_A$* pair if the *hgl* island did not contain an *hglG* hit. Three islands did not contain an ORF with an *hglE$_A$* hit, and in those cases, we based location on the *hglG* hit as if it was at location *hglE$_A$* + 2 with orientation based on the *hglT* / *hglG* pair. If an ORF contained multiple non-overlapping hits to the same gene, e.g. a duplication of *hglE$_A$*, it was counted as 1 hit in this plot. Pie charts show the copy number of hits on the islands—for example, in 12% of the islands that contain an *hgdA* hit, two *hgdA* hits are present on two different ORFs. ORF, open reading frame.

a ***Hgl*** **islands with homologs of ≥10 *Anabaena* sp. PCC 7120 genes (n = 393, in 389 genomes)**

	hglT	*all5342*	***all5343***	***all5344***	***hgdA***	***hgdC***	***hgdB***	*alr5348*	*asr5349*	*asr5350*	***hglE$_A$***	***hglF***	***hglG***	***hglD***	***hglC***	***hglA***	***hglB***	*hetN*	*hetI*
**Fraction of islands:**	0.88	0.13	0.88	0.90	0.96	0.98	0.98	0.45	0.00	0.49	0.99	0.99	0.99	0.97	0.98	0.97	0.94	0.64	0.52

b ***Hgl*** **islands with homologs of ≥10 *Anabaena* sp. PCC 7120 genes (n = 393, in 389 genomes)**

	hglT	*all5342*	***all5343***	***all5344***	***hgdA***	***hgdC***	***hgdB***	*alr5348*	*asr5349*	*asr5350*	***hglE$_A$***	***hglF***	***hglG***	***hglD***	***hglC***	***hglA***	***hglB***	***hetN***	***hetI***
**Fraction of genomes:**	1.00	0.39	0.96	0.99	1.00	1.00	1.00	0.48	0.00	0.51	1.00	1.00	1.00	1.00	1.00	1.00	1.00	1.00	1.00

Supplementary Fig. 8. Some genes are absent from the *hgl* island but present elsewhere on the genome. a, Frequency of homologs of *Anabaena* sp. PCC 7120 HG biosynthesis genes on *hgl* islands containing homologs of ≥10 genes. The figure is identical to Fig. 2c. **b**, Frequency of homologs of HG biosynthesis genes in the genome of the cyanobacteria that encode the islands of panel a. Color-coding in panels a and b is for legibility only and reflects the numbers within the cells, with darker colors representing a higher fraction. Genes that are present in ≥88% of the islands or genomes are indicated in bold in panel a and b, respectively.

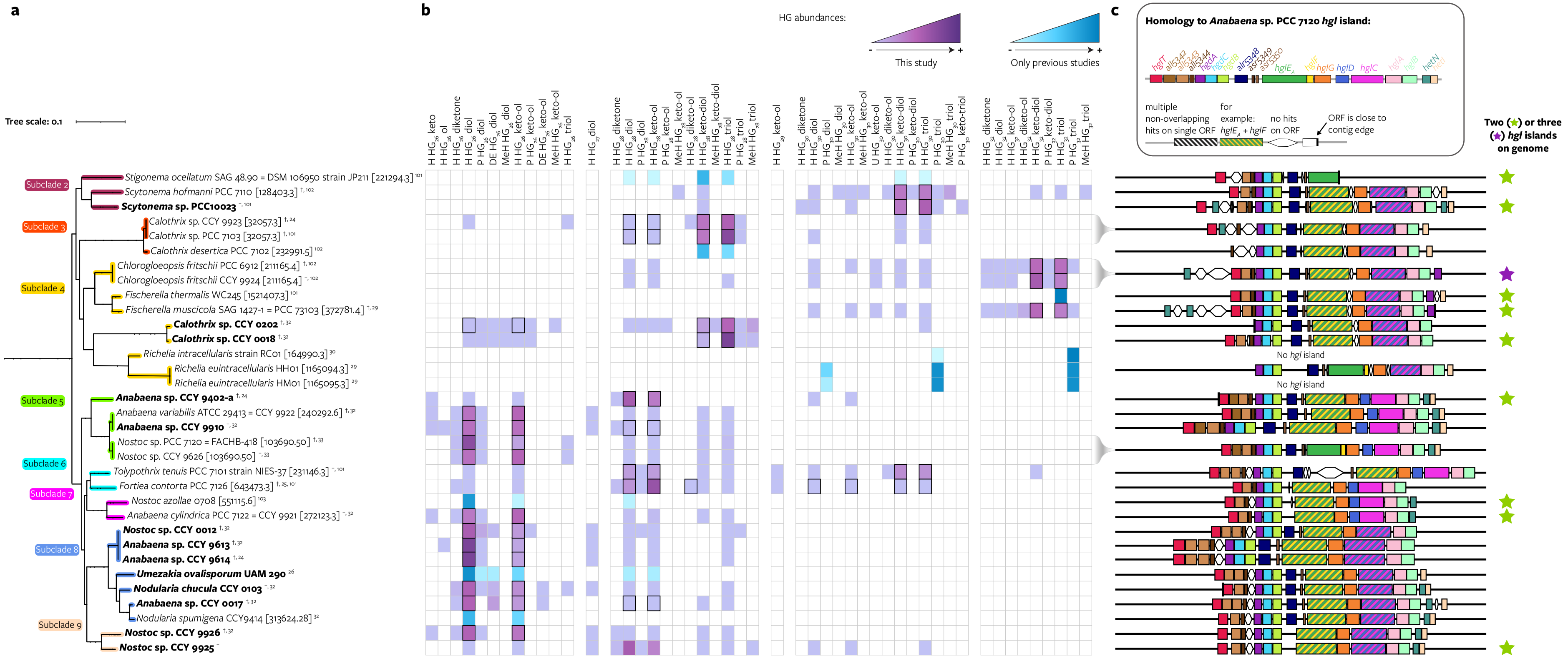

Supplementary Fig. 9. Distribution of 49 HGs throughout heterocytous *Cyanobacteriia*. The figure is identical to Fig. 3 except for panel b. **a**, Maximum likelihood cyanobacterial phylogeny based on 24 core vertically-transferred genes including genomes with a known HG lipid profile (pruned from Fig. 2a). Genomes sequenced in this study are shown in bold. Superscripts indicate studies where HG data of each strain were analyzed. **b**, Relative abundances of each HG characteristic are calculated in relation to the sum of all HGs produced by each strain. HG abundances obtained from either this study or previous studies are shown per row as purple or blue heatmaps, respectively. When data from both literature and this study were available only the data collected in this study are shown in the heatmap. HGs detected in previous studies are indicated by a black cell border. Data originally represented in the literature as symbols were converted to percentages as follows; "+++", 90%; "++", 40%, "+", 15%; "tr.", 1%. Data used to generate this heatmap are found in Supplementary Table 10 (24-27, 29, 30, 32, 33, 101-103), † this work, H, hexose; DeH, deoxyhexose; MeH, methyl-hexose; P, pentose; U, unknown. **c**, Schematic representation of the genes present on the most extended *hgl* island on the genome in terms of number of *Anabaena* sp. PCC 7120 genes with homologs on the island. Stars indicate the presence of two (green) or three (purple) *hgl* islands on the genome; these additional islands are not drawn. ORF, open reading frame; contig, contiguous sequence.

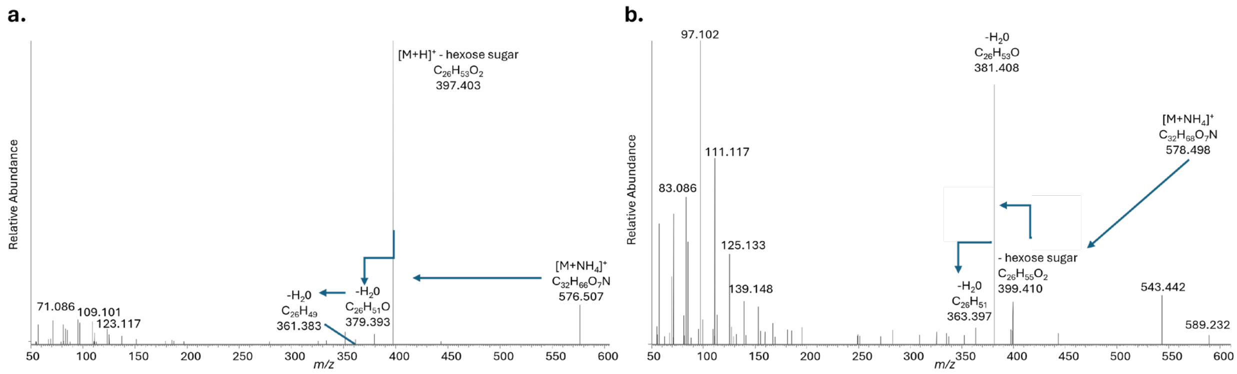

Supplementary Fig. 10. UHPLC-HRMS MS^2 spectra of unusual, novel HGs. a, MS^2 spectrum of the $[M+NH_4]^+$ ion at *m/z* 576.483 identified as hexose HG_{26} keto. **b**, MS^2 spectrum of the $[M+NH_4]^+$ ion at *m/z* 578.499 identified as hexose HG_{26} ol.

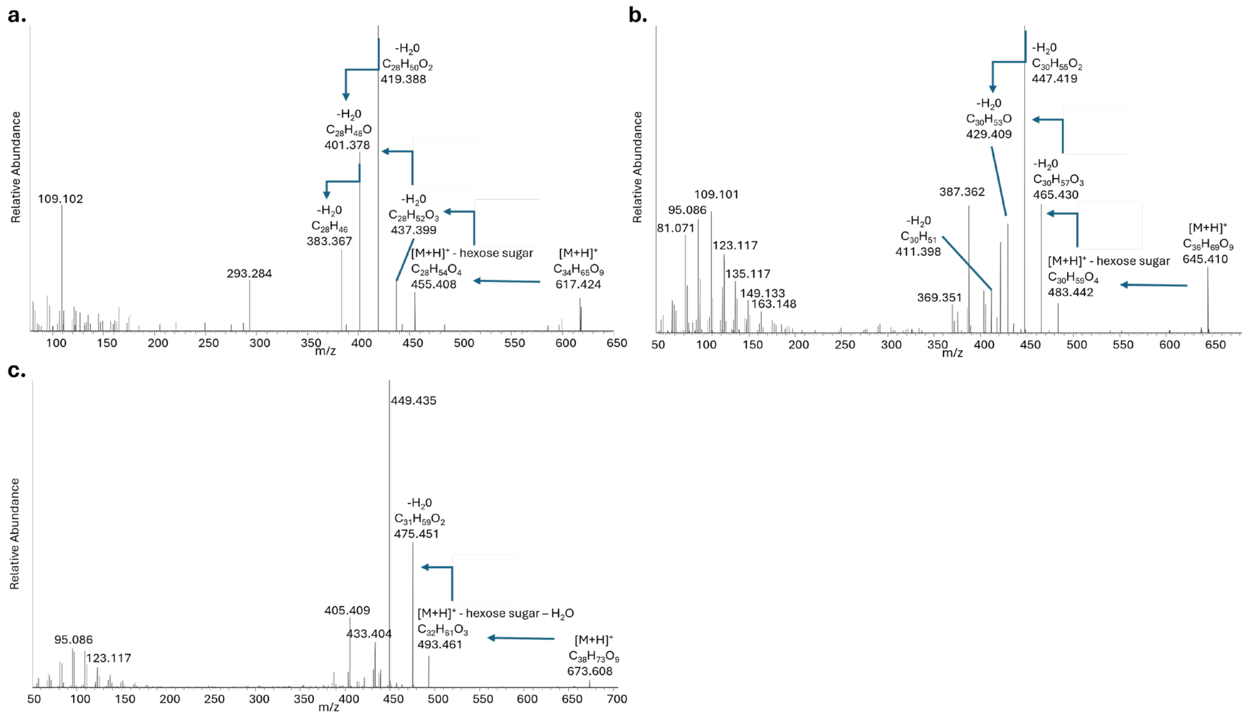

Supplementary Fig. 11. UHPLC-HRMS MS^2 spectra of unusual, novel HGs. a, MS^2 spectrum of the $[M+H]^+$ ion at *m/z* 617.462 identified as HG_{28} diketo-ol. **b**, MS^2 spectrum of the $[M+H]^+$ ion at *m/z* 645.494 identified as hexose HG_{30} diketo-ol. **c**, MS^2 spectrum of the $[M+H]^+$ ion at *m/z* 673.525 identified as hexose HG_{32} diketo-ol.

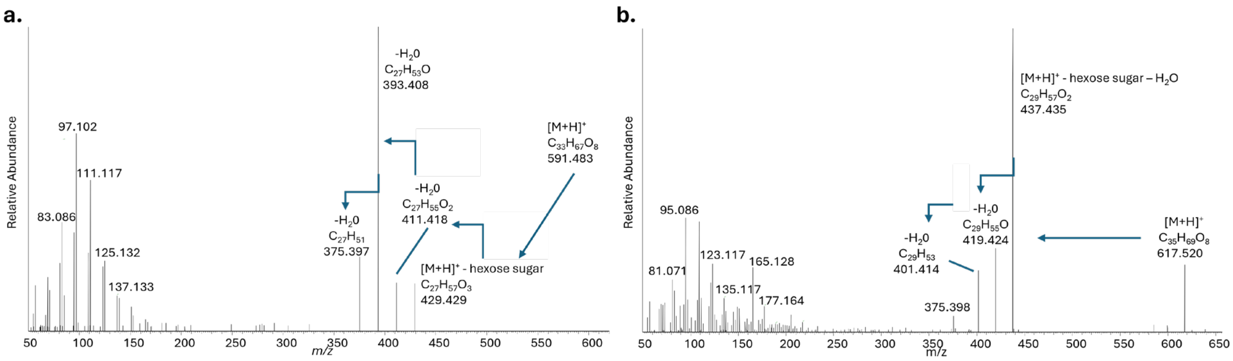

Supplementary Fig. 12. UHPLC-HRMS MS^2 spectra of unusual, novel HGs. a, MS^2 spectrum of the $[M+H]^+$ ion at *m/z* 591.484 identified as hexose HG_{27} diol. **b**, MS^2 spectrum of the $[M+H]^+$ ion at *m/z* 617.49 identified as hexose HG_{29} keto-ol.

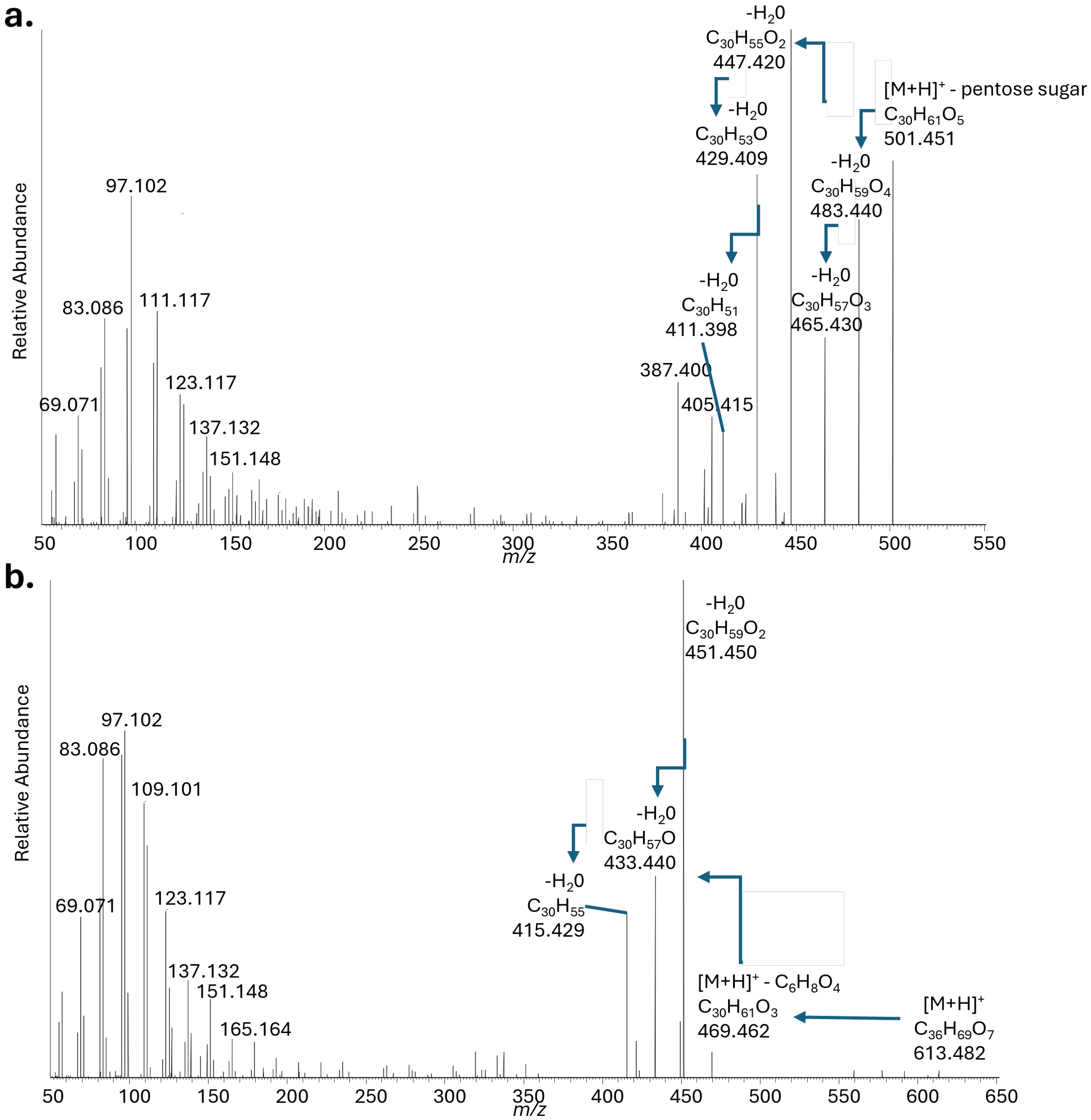

Supplementary Fig. 13. a, UHPLC-HRMS MS^2 spectrum of the $[M+H]^+$ ion at *m/z* 633.530 identified as pentose HG_{30} keto-triol. **b,** MS^2 spectrum of the $[M+H]^+$ ion at *m/z* 613.504 identified as HG-like compound, C_{30} keto-ol with an unknown headgroup.

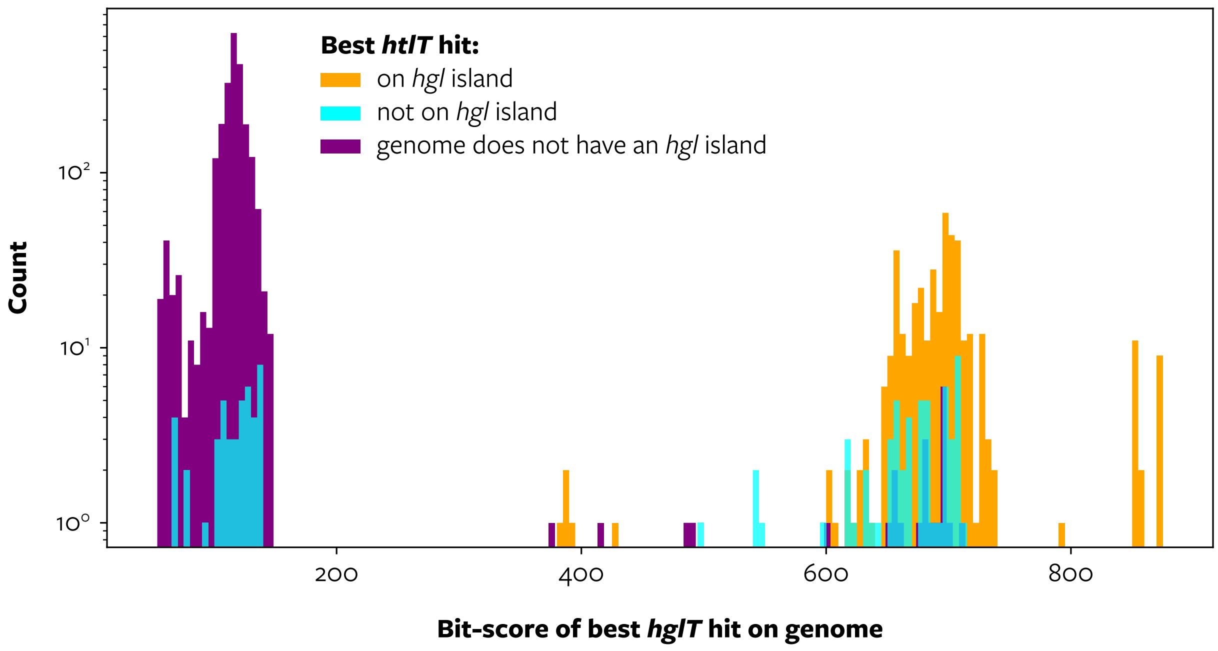

Supplementary Fig. 14. Representation of the number of genomes (y-axis) for which the best *hglT* hit has a given bit-score (x-axis). Hits with a bit-score <200 may be indicative of distant homologs with a potentially divergent function unrelated to HG biosynthesis. Colors represent whether the genome contains an *hgl* island as defined here (cyan and orange) and if so, if the best *hglT* hit is found within the island (orange) or elsewhere (cyan). Genomes without an island are shown in purple. Genomes were binned in cohorts of 5 bit-scores. Note the logarithmic scale of the y-axis.

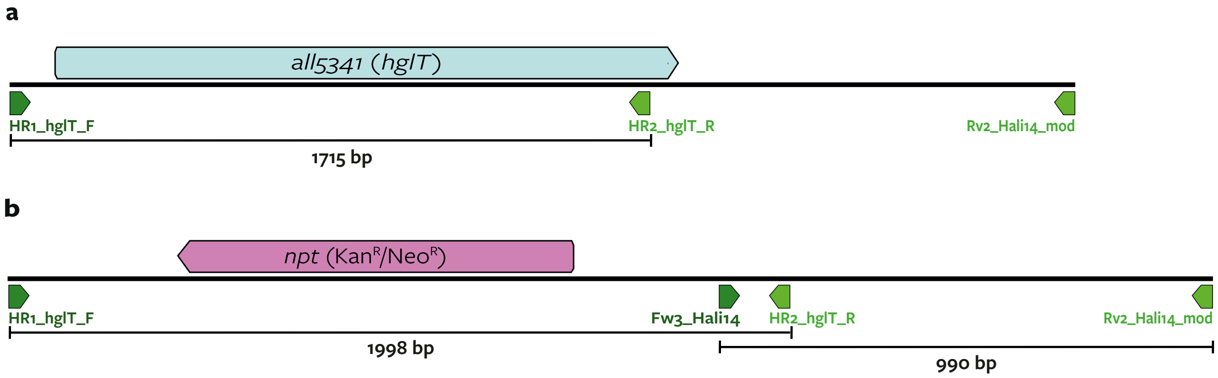

Supplementary Fig. 15. Schematic representation of *hglT* (*all5341*). a, in the *wild-type Anabaena* sp. PCC 7120 strain **b,** in the Δ*hglT* mutant strain where *hglT* is replaced by *npt* gene conferring resistance to kanamycin (Kan^R) and neomycin, (Neo^R)**.** HR1_hglT_F, HR2_hglT_R, Rv2_Hali14_mod and Fw3_Hali14, primer annealing sites. Lines and numbers indicate the expected length of the PCR product.

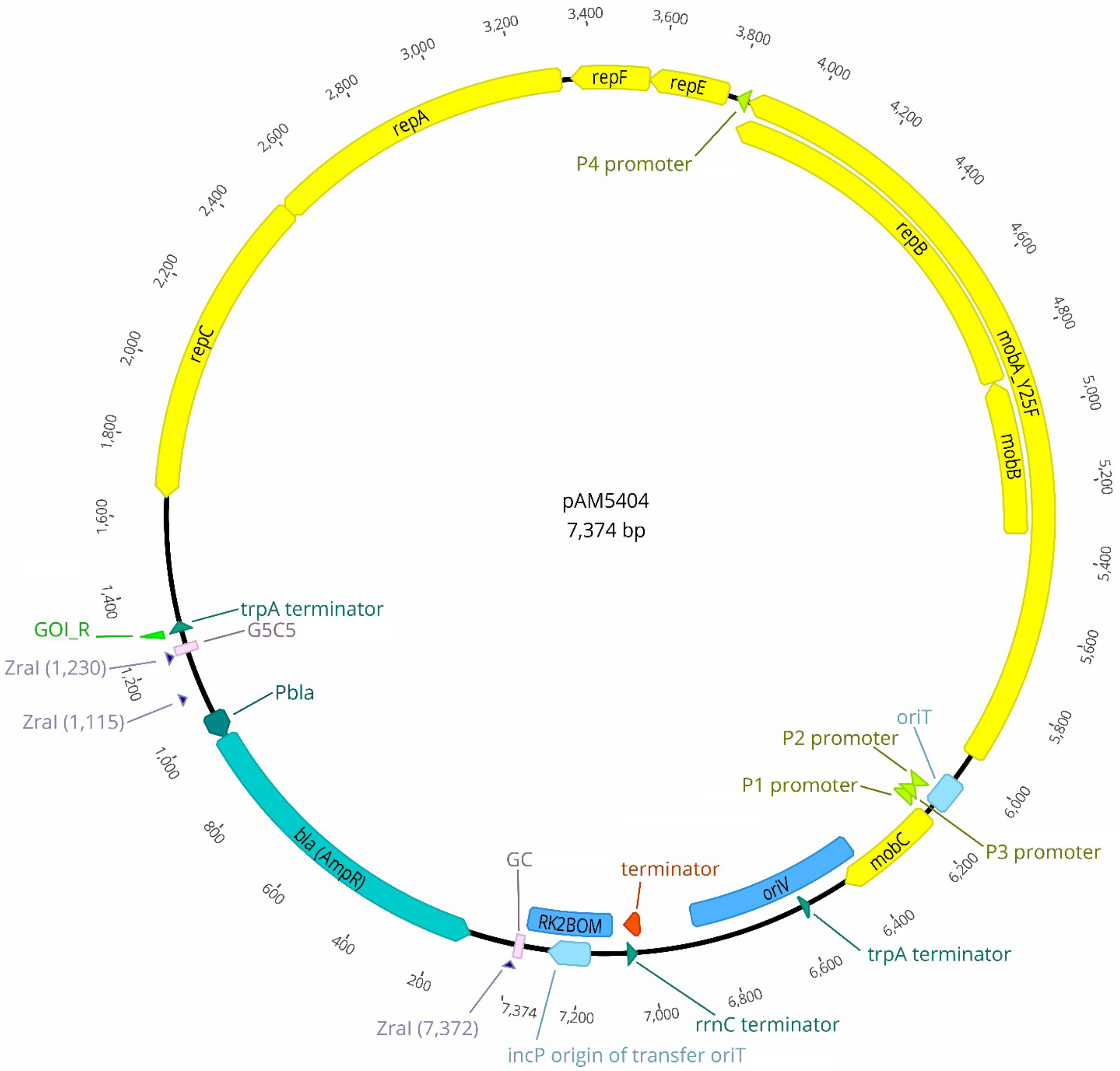

Supplementary Fig. 16. Schematic representation of the pAM5404 cargo plasmid. *repA*, *repB*, *repC*, *repE* and *repF*, genes required for plasmid replication; *mobA*, *mobB* and *mobC*, genes responsible for plasmid mobilization, *mobA* contains Y25F mutation responsible for improving cloning efficiency; *oriV*, RSF1010 broad-host-range plasmid origin of replication; *oriT*, RSF1010 plasmid origin of transfer; *incP*, RP4 conjugal plasmid origin of transfer; *RK2bom*, RK2 conjugal plasmid origin of transfer; P1, P2 and P3, RSF1010 native promoters; P_{bla}, ampicillin antibiotic resistance cassette promoter; bla (AmpR), ampicillin antibiotic resistance cassette; ZraI, restriction sites for ZraI enzyme; GOI_R, primer annealing site. GC and G5C5, GC-adaptor sequences described in ref. (93) and used for cloning of the GOIs and antibiotic resistance cassette.

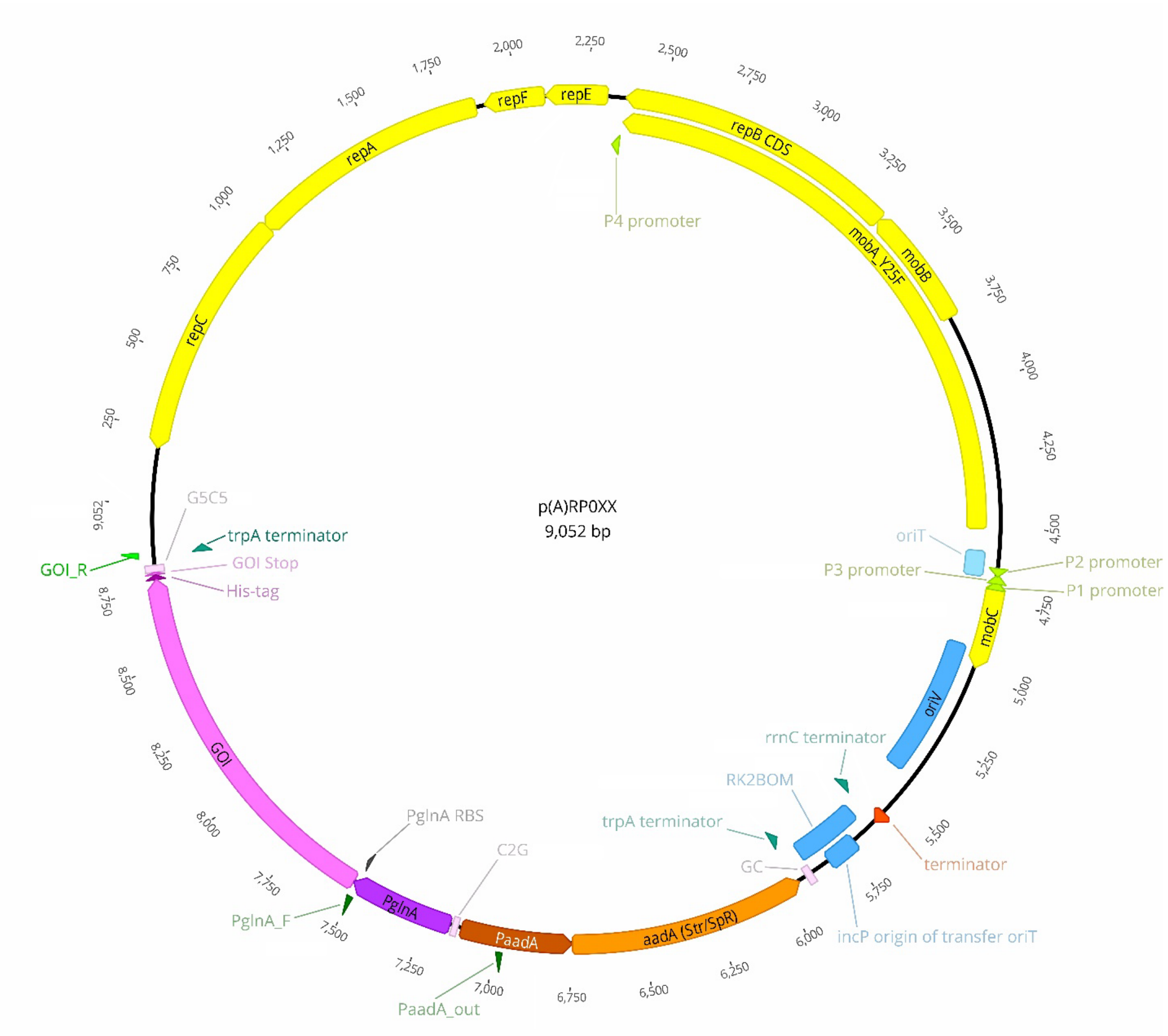

Supplementary Fig. 17. Common schematic representation of plasmids pRP012-14, pRP019-022. pRP019 and pRP020 do not possess a His-tag. GOI, gene of interest (all5341, RINTHH_17770, RINTHH_20790, RINTHH_5560-RINTHH_5570); P_{glnA}, glnA promoter; P_{aadA}, Streptomycin and spectinomycin resistance cassette promoter, *aadA*, spectinomycin and streptomycin resistance cassette. *repA*, *repB*, *repC*, *repE* and *repF*, genes required for plasmid replication; *mobA*, *mobB* and *mobC*, genes responsible for plasmid mobilization, *mobA* contains Y25F mutation responsible for improving cloning efficiency; oriV, RSF1010 broad-host-range plasmid origin of replication; oriT, RSF1010 plasmid origin of transfer; incP, RP4 conjugal plasmid origin of transfer; RK2bom, RK2 conjugal plasmid origin of transfer; P1, P2 and P3, RSF1010 native promoters; P_{bla} , ampicillin antibiotic resistance cassette promoter; bla (AmpR), ampicillin antibiotic resistance cassette; ZraI, restriction sites for ZraI enzyme; GOI_R, primer annealing site. GC and G5C5, GC-adaptor sequences described in ref. (93) and used for cloning of the GOIs and antibiotic resistance cassette.

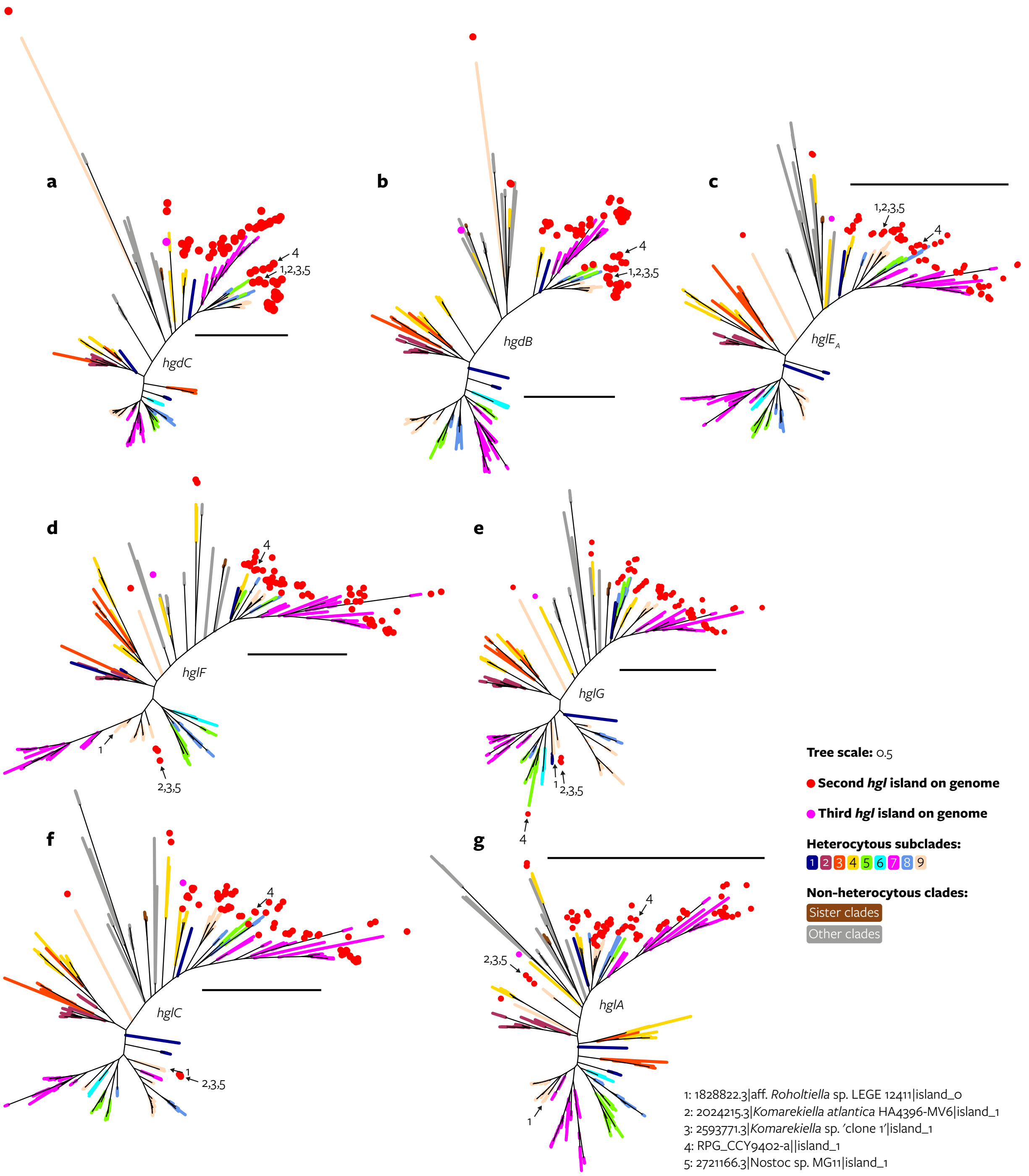

Supplementary Fig. 18. Individual gene trees of homologs of seven HG biosynthesis genes that are often present on *hgl* islands (a-g). The seven phylogenies have similar tree topologies—with additional islands of heterocytous cyanobacteria forming a distinct group, and the *hgl* islands of non-heterocytous cyanobacteria branching in between this group and the most extended *hgl* islands on the genomes of heterocytous cyanobacteria. Five heterocytous islands have an incongruent evolutionary history and are indicated with arrows and numbers 1-5 (legend in lower right; the island names have zero-based numbering, where 'island_0' is the first island). 'First', 'second', and 'third' hgl islands are based on the presence of other islands on the genome, where the 'first' hgl island (not marked) is the most extended island in terms of number of Anabaena sp. PCC 7120 genes with homologs on the island, the second hgl island (red dot) the second-most extended island, and the third island (pink dot) the third-most extended island. Scale bars represent a mean number of 0.5 substitutions per site. Source files in Supplementary Data 7.

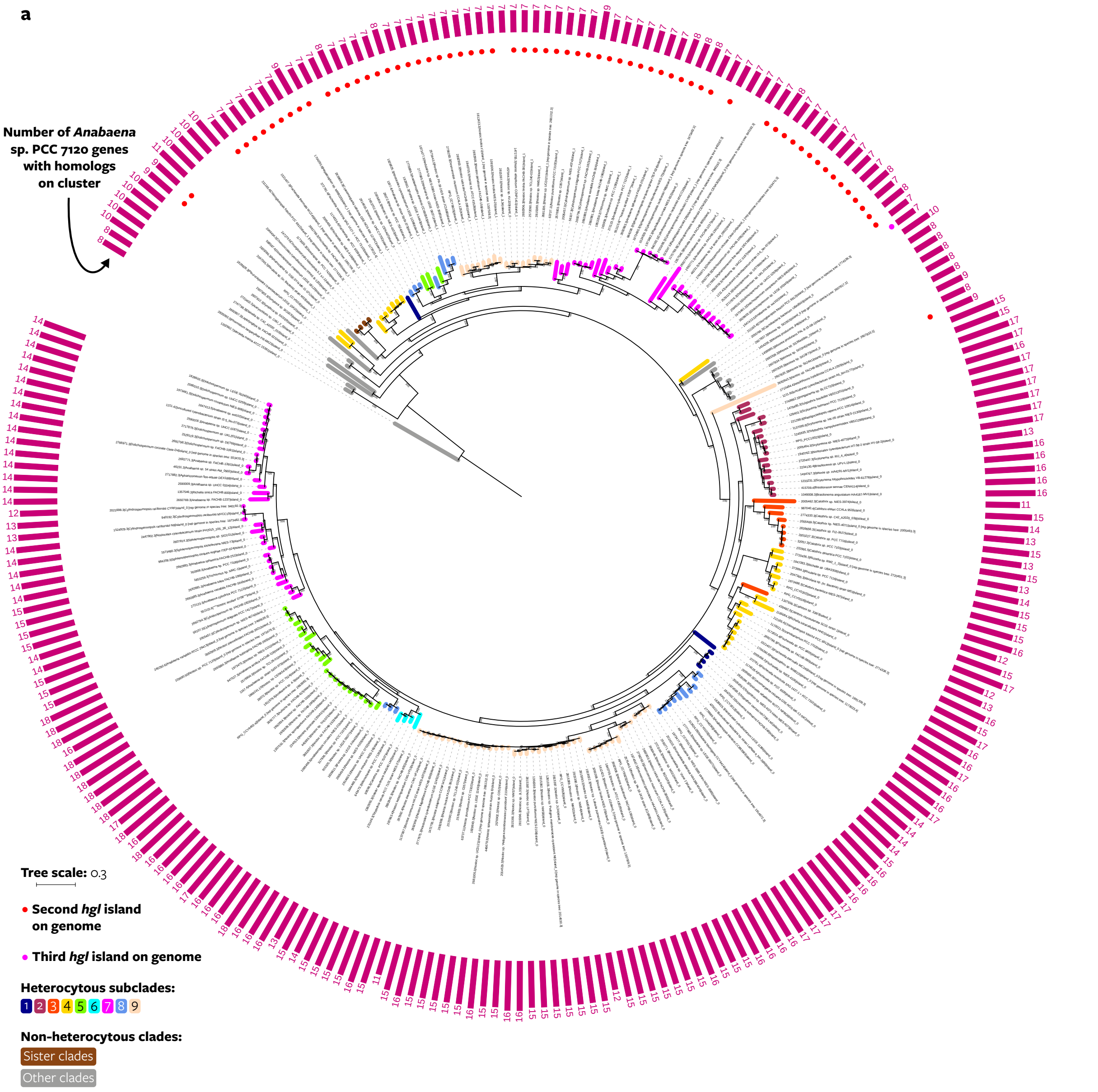

b Presence of different genera in defined clades containing:

i) One island

Clade / genus	Genome count
**Sister clades**	
Chroococcales	1
Chroogloeocystis	1
Gloeocapsa	1
Gloeocapsopsis	1
**Subclade 1**	
Tolypothrix	2
Scytonema	1
Spirirestis	1
**Subclade 2**	
Scytonema	5
Brasilonema	3
Tolypothrix	2
Aetokthonos	1
Iningainema	1
Iphinoe	1
Mastigocladopsis	1
no genus annotation	1
Nostocales	1
**Subclade 3**	
Calothrix	9
**Subclade 4**	
Richelia	3
Calothrix	2
Fischerella	1
no genus annotation	1
Rivularia	1
**Subclade 5**	
Nostoc	13
Anabaena	4
Anabaenopsis	1
Aulosira	1
**Subclade 6**	
Calothrix	1
Goleter	1
Microchaete	1
Tolypothrix	1
**Subclade 7**	
Raphidiopsis	2
Anabaena	1
Cylindrospermopsis	1
no genus annotation	1
Sphaerospermopsis	1
**Subclade 8**	
Nodularia	2
Nostoc	2
**Subclade 9**	
Nostoc	20
Komarekiella	2
Desmonostoc	1
no genus annotation	1

ii) Two islands

Clade / genus	Genome count
**Subclade 1**	
Cyanomargarita	1
**Subclade 4**	
Fischerella	5
Hapalosiphon	1
Rivularia	1
**Subclade 5**	
Anabaena	1
Fortiea	1
Nostoc	1
**Subclade 7**	
Anabaena	10
Dolichospermum	7
Cylindrospermopsis	3
Cylindrospermum	3
Sphaerospermopsis	2
Aphanizomenon	1
no genus annotation	1
Nostoc	1
Richelia	1
Trichormus	1
**Subclade 8**	
Nodularia	2
Nostoc	2
Anabaenopsis	1
Nostocales	1
**Subclade 9**	
Nostoc	15
Desmonostoc	1

iii) Three islands

Clade / genus	Genome count
**Subclade 4**	
Chlorogloeopsis	1

Supplementary Fig. 19. Annotated tree of a concatenated alignment of homologs of seven HG biosynthesis genes. (Caption on next page).

Supplementary Fig. 19. Annotated tree of a concatenated alignment of homologs of seven HG biosynthesis genes. The tree structure is identical to Fig. 4, except for the distance to the *pfa* synthase of the gammaproteobacterium *Moritella marina* ATCC 15381, which is drawn to scale here and artificially shortened in Fig. 4. 'First', 'second', and 'third' *hgl* islands are based on the presence of other islands on the genome, where the 'first' hgl island (not marked) is the most extended island in terms of number of *Anabaena* sp. PCC 7120 genes with homologs on the island, the second *hgl* island (red dot) the second-most extended island, and the third island (pink dot) the third-most extended island. The island names in this panel have zero-based numbering, where 'island_0' is the first island. Ultrafast bootstrap approximation values are indicated along the branches of the tree. Scale bar represents the mean number of substitutions per site. Source files in Supplementary Data 7. An unrooted version can be found in Supplementary Fig. 20. **b**, Distribution of genera within the different heterocytous subclades and sister clades that are included in the tree in panel a. The genus names are based on the taxonomic annotation in the 'genome_lineage' file on the PATRIC ftp server.

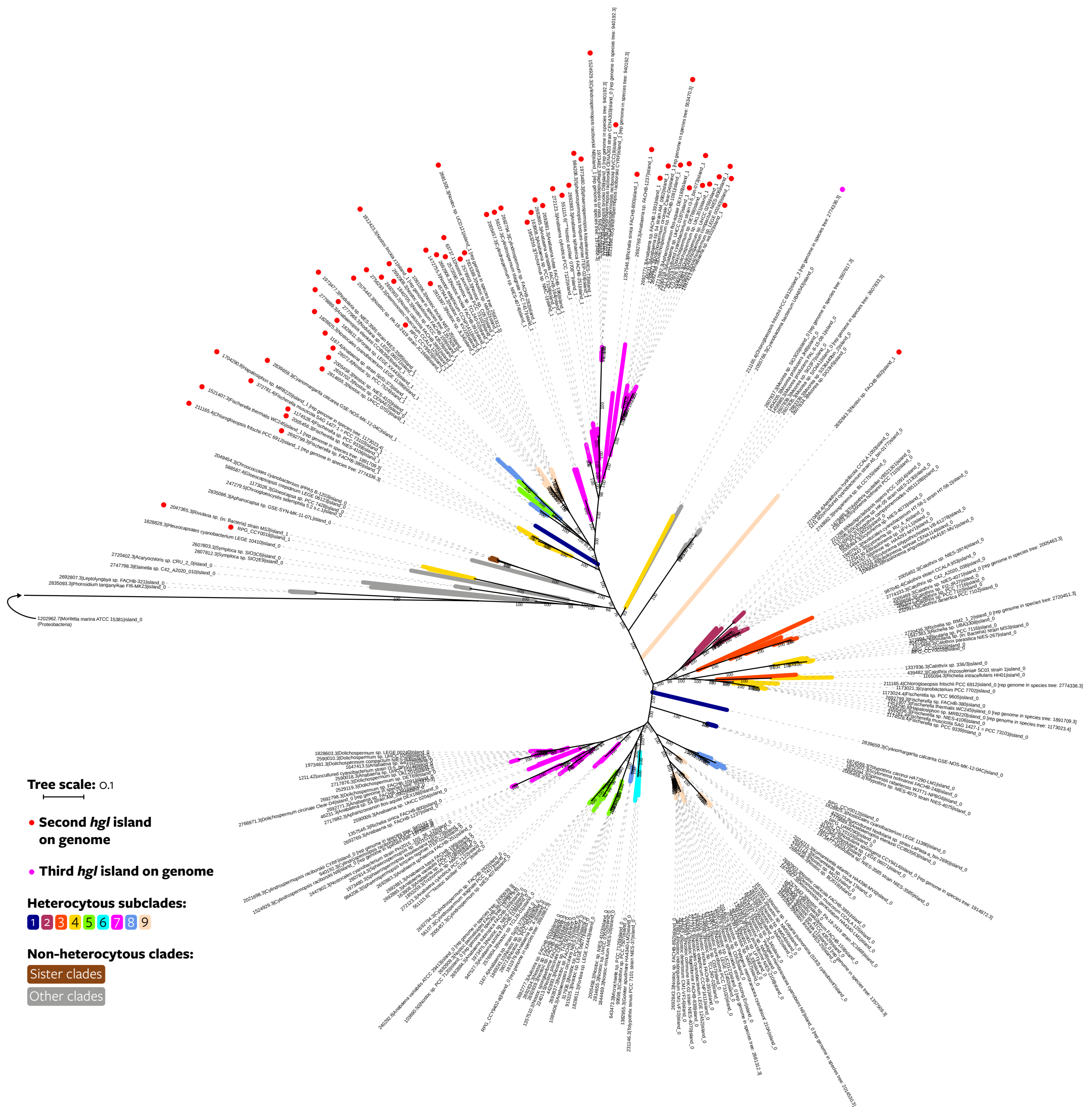

Supplementary Fig. 20. The same phylogeny of a concatenated alignment of homologs of seven HG biosynthesis genes (*hgdCB* and *hglE$_A$FGCA*) as Fig. 4 but drawn unrooted. The branch to the *pfa* synthase of the gammaproteobacterium *Moritella marina* ATCC 15381 is drawn to scale—not artificially shortened as in Fig. 4. 'First', 'second', and 'third' *hgl* islands are based on the presence of other islands on the genome, where the 'first' *hgl* island (not marked) is the most extended island in terms of number of *Anabaena* sp. PCC 7120 genes with homologs on the island, the second *hgl* island (red dot) the second-most extended island, and the third island (pink dot) the third-most extended island. The island names in this figure have zero-based numbering, where 'island_0' is the first island. Ultrafast bootstrap approximation values are indicated along the branches of the tree. Scale bar represents the mean number of substitutions per site. Source files in Supplementary Data 7.

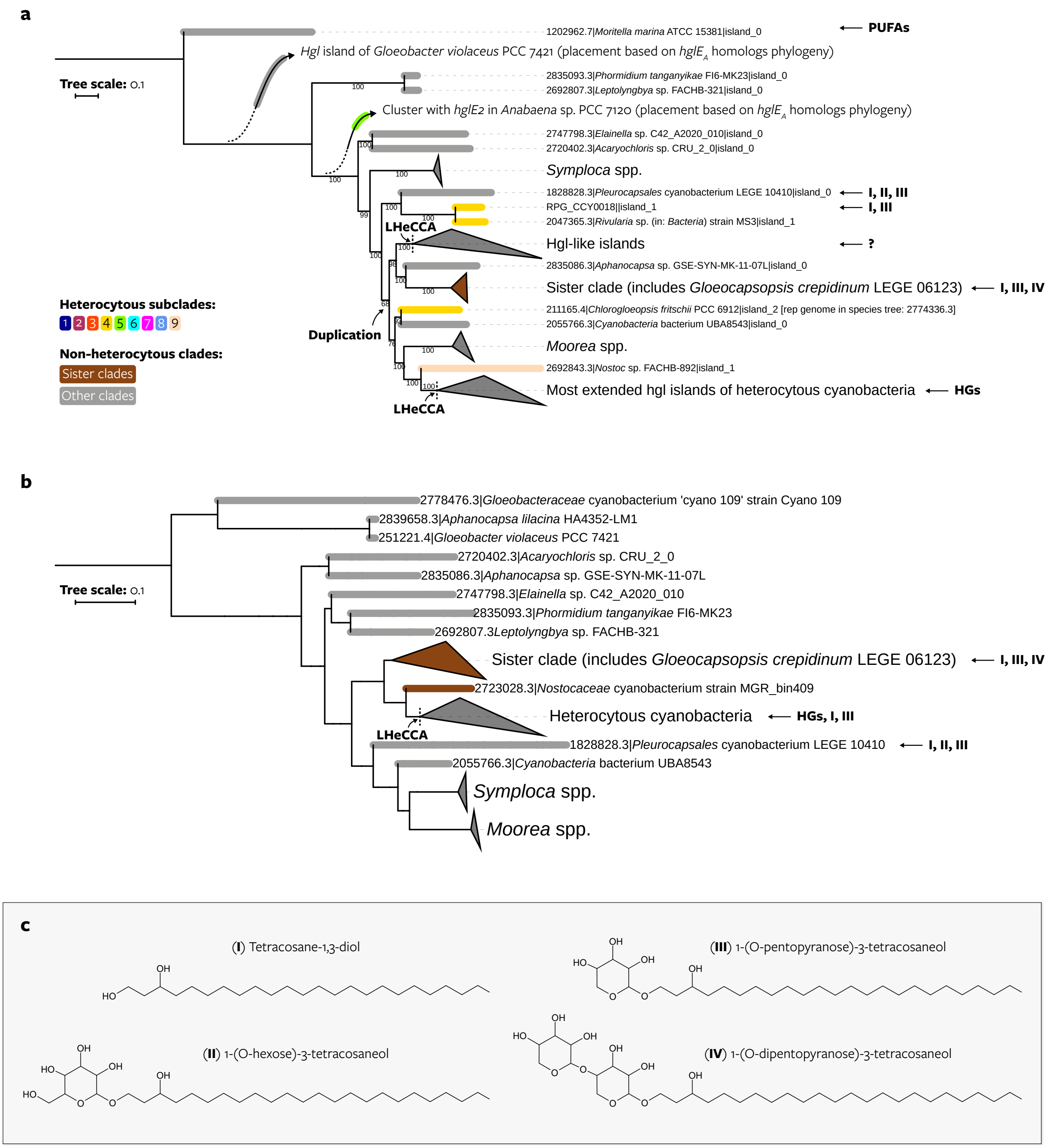

Supplementary Fig. 21. Evolutionary history of HG biosynthesis within the *Cyanobacteriia*. a, The same phylogeny of a concatenated alignment of homologs of seven HG biosynthesis genes (*hgdCB* and *hglE$_A$FGCA*) as Fig. 4 but with collapsed clades for legibility. Likely duplication events and LHeCCA are indicated along the branches. The *hgl* island of *Gloeobacter violaceus* PCC 7421 and of the cluster containing hglE2 in *Anabaena* sp. PCC 7120 are not included in this phylogeny as they do not contain homologs of all seven HG biosynthesis genes, and their putative placement here is based on the phylogeny of *hglE$_A$* homologs (Supplementary Figs. 24 and 25). The island names in this panel have zero-based numbering, where 'island_0' is the first island. **b**, The same phylogeny of a concatenated alignment of 24 core vertically-transferred genes as Fig. 2a, but with collapsed clades and pruned clades for legibility. The pruned tree contains all non-cyanobacterial genomes with an *hgl* island. Known biosynthetic products produced by cultures (PUFAs, HGs, and roman numerals I-IV) are indicated to the right in panels a and b, where roman numerals represent the HG analogs identified in this study and depicted in panel c. Ultrafast bootstrap approximation values are indicated along the branches of the trees in panels a and b. Scale bars in panels a and b represent the mean number of substitutions per site. **c**, Tentative structure of HG analogs.

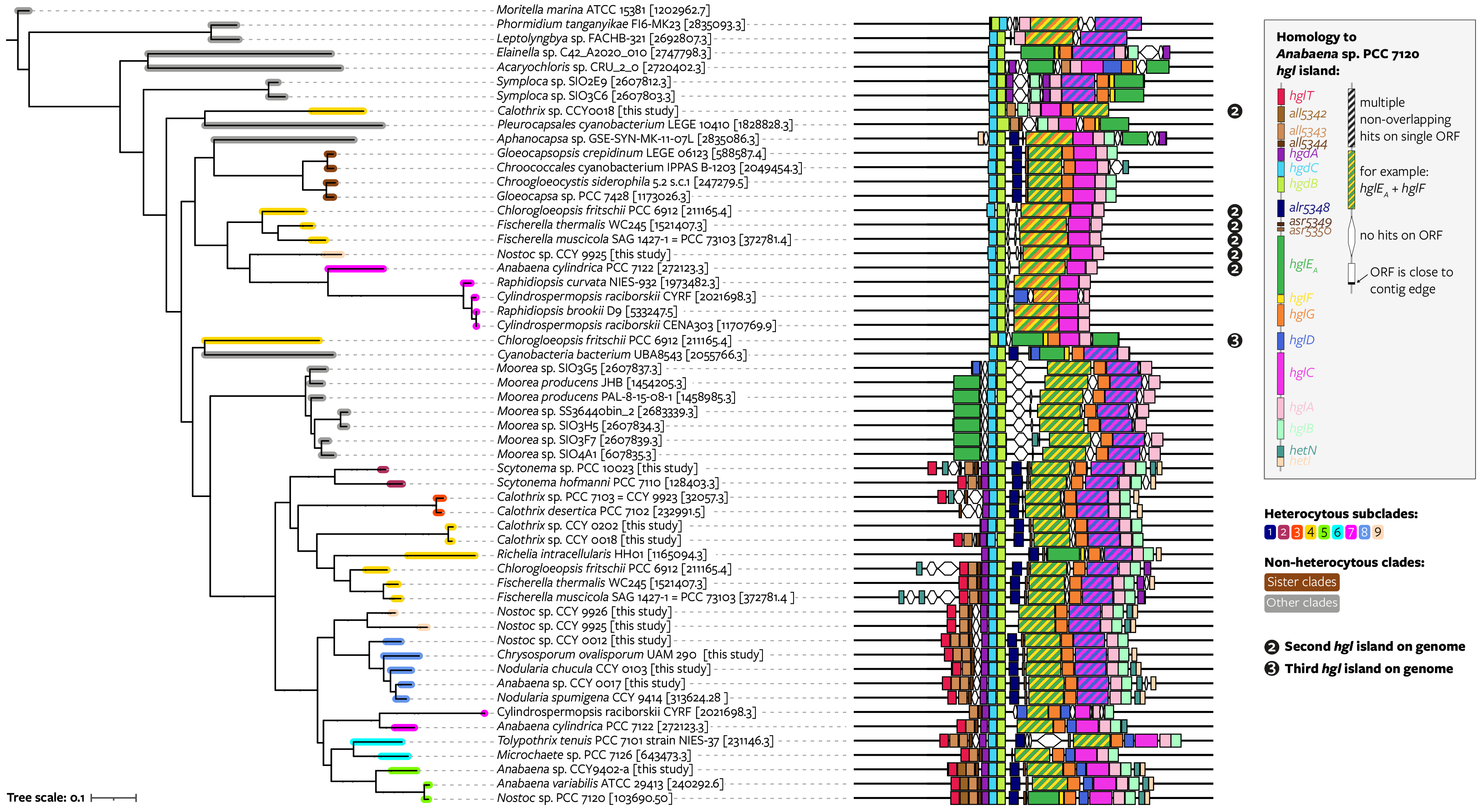

Supplementary Fig. 22. Phylogenetic relationship between selected *hgl* and *hgl*-like islands. (Caption on next page).

Supplementary Fig. 22. Phylogenetic relationship between selected *hgl* and *hgl*-like islands. The maximum likelihood phylogenetic tree of concatenated *hgdCB* and *hglE$_A$FGCA* homologous sequences of *hgl* islands is pruned from Fig. 4. All *hgl* islands from strains with a known HG lipid profile and from all non-heterocytous cyanobacterial strains that contain an *hgl* island and that are shown in Fig. 4 are included. In addition, the islands of *Raphidiopsis curvata* NIES-932, *Cylindrospermopsis raciborskii* CENA303, and *Raphidiopsis brookii* D9 are included because they are non-diazotrophic cyanobacteria from within the heterocytous clade. Their single islands cluster with the *hgl*-like islands of heterocytous cyanobacteria instead of with their more extended *hgl* islands. In addition, the two islands of *Cylindrospermopsis raciborskii* CYRF are included because its *hgl* islands lack *hglT*. 'First', 'second', and 'third' *hgl* islands are based on the presence of other islands on the genome, where the 'first' *hgl* island (not marked) is the most extended island in terms of number of *Anabaena* sp. PCC 7120 genes with homologs on the island, the second *hgl* island (marked with a 2) the second-most extended island, and the third island (marked with 3) the third-most extended island. The tree is rooted in between the cyanobacterial *hgl* islands and the *pfa* synthase of the gammaproteobacterium *Moritella marina* ATCC 15381. The root is artificially shortened for legibility. Scale bar represents the mean number of substitutions per site. ORF, open reading frame; contig, contiguous sequence.

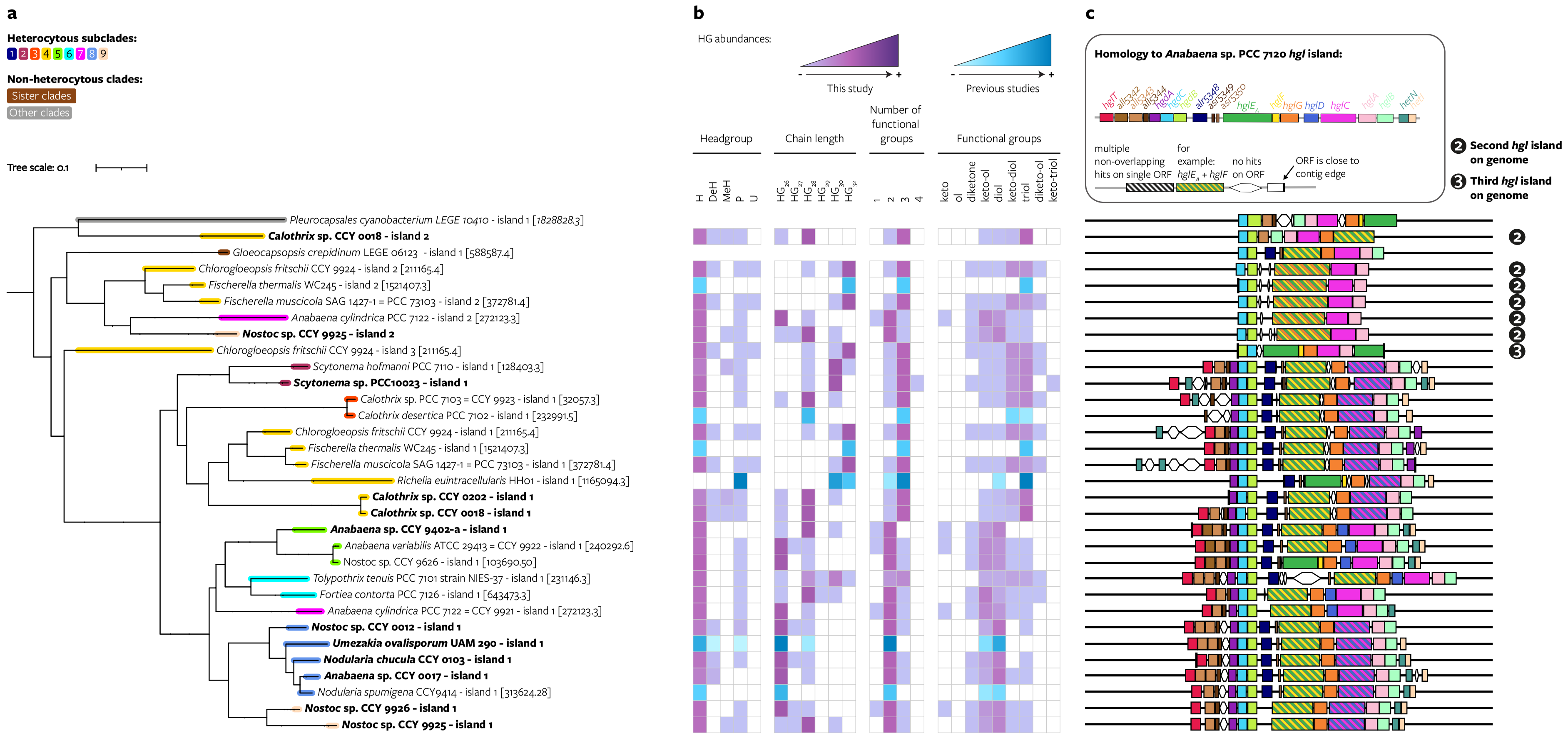

Supplementary Fig. 23. Distribution of 49 HGs grouped according to their structure's characteristics throughout Cyanobacteriia and plotted on the hgl island phylogeny. (Caption on next page).

Supplementary Fig. 23. Distribution of 49 HGs grouped according to their structure's characteristics throughout *Cyanobacteriia* and plotted on the *hgl* island phylogeny. a, Maximum likelihood phylogeny of the *hgl* island created using a concatenated alignment of homologous sequences of seven HG biosynthesis genes (*hgdCB* and *hglE$_A$FGCA*) that are often present on *hgl* islands, including only genomes with a known HG lipid profile (pruned, see Online Methods). Genomes sequenced in this study are shown in bold. Some genomes contain multiple *hgl* islands and are thus present in the tree multiple times. **b**, Heatmap of HG relative abundances grouped according to the headgroup (H, hexose; DeH, deoxyhexose; MeH, methyl-hexose; P, pentose; U, unknown), chain length, number and type of functional groups of each HG. Relative abundances of each HG characteristic are calculated in relation to the sum of all HGs produced by each strain. HG abundances obtained from either this study or previous studies are shown per row as purple or blue heatmaps, respectively. When data from both literature and this study were available only the data collected in this study are shown in the heatmap. Data originally represented in the literature as symbols were converted to percentages as follows; "+++", 90%; "++", 40%, "+", 15%; "tr.", 1%. Data used to generate this heatmap are found in Supplementary Table 10. HG relative abundances obtained from this study are shown in purple and those obtained from literature are shown in blue. **c**, Schematic representation of the genes present on the *hgl* island. When more than one island is present in the genome of the strain, the same HG abundances heatmap is shown for each island. 'First', 'second', and 'third' *hgl* islands are based on the presence of other islands on the genome, where the 'first' *hgl* island (not marked) is the most extended island in terms of number of *Anabaena* sp. PCC 7120 genes with homologs on the island, the second *hgl* island (marked with a 2) the second-most extended island, and the third island (marked with 3) the third-most extended island. ORF, open reading frame; contig, contiguous sequence.

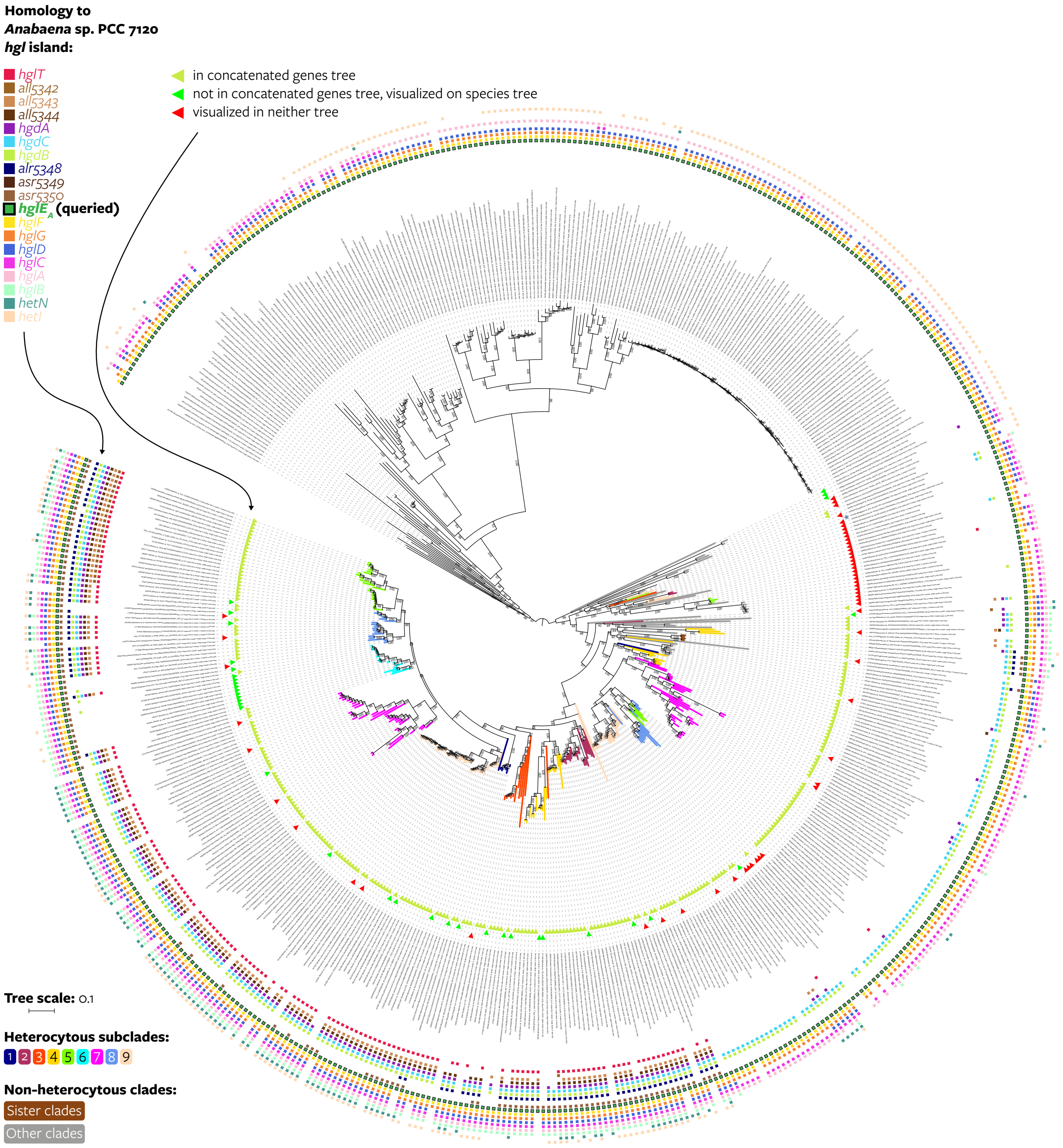

Supplementary Fig. 24. Phylogeny of *hglE$_A$* homologs. The presence of homologs of other queried genes on the cluster is indicated as a ring around the tree. The *hglE$_A$* hit of the single *Proteobacteria* genome (the MAG *Beggiatoa* sp. 4572_84) that branches from within the *Cyanobacteriia* is indicated with a grey star. Ultrafast bootstrap approximation values are indicated along the branches of the tree. Scale bar represents the mean number of substitutions per site. Source files in Supplementary Data 8.

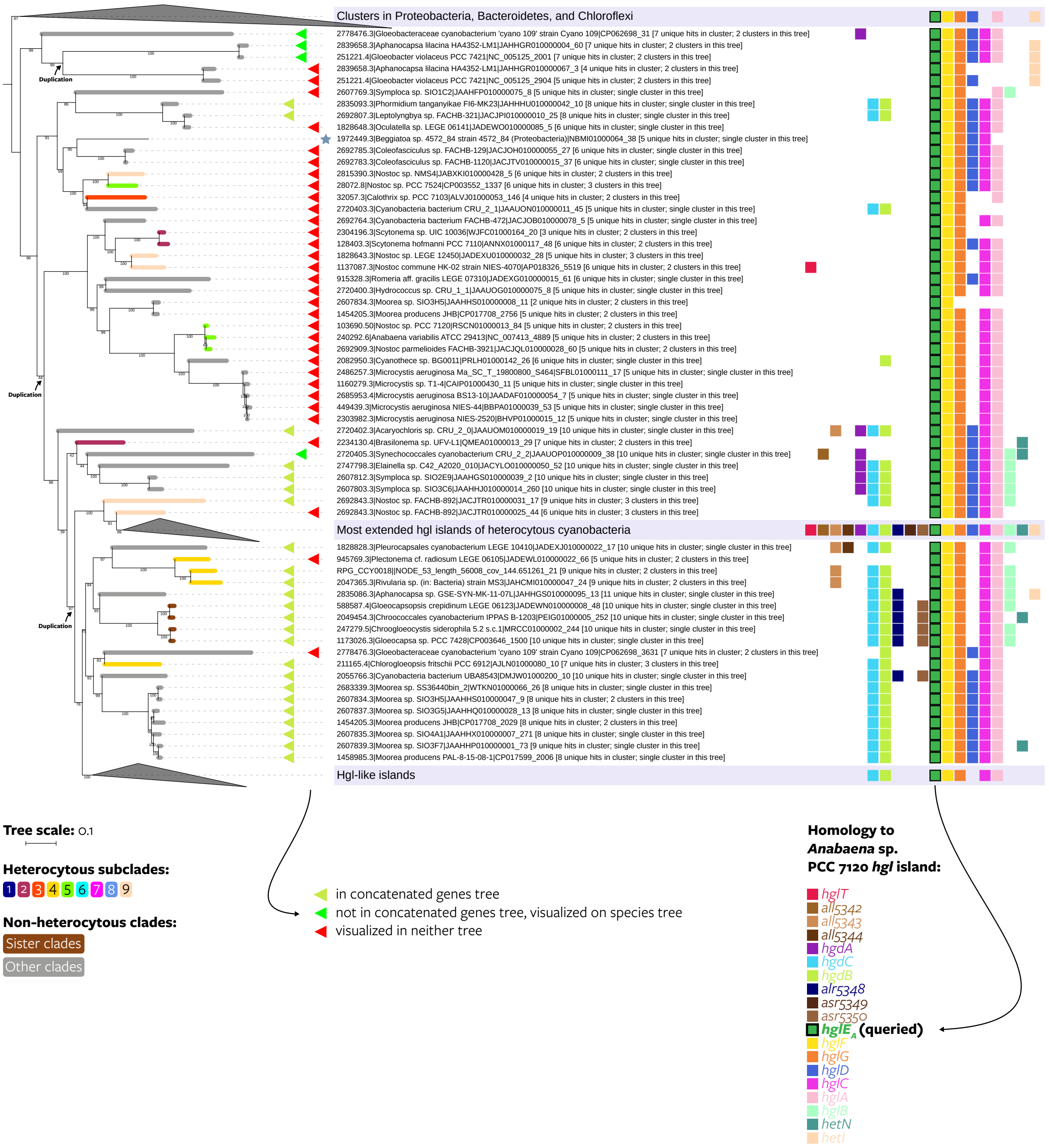

Supplementary Fig. 25. The same phylogeny of *hglE$_A$* homologs as Supplementary Fig. 24 but with collapsed clades for legibility. Likely duplication events are indicated along the branches. The clusters drawn on the collapsed clades correspond to either the majority of the clusters in that clade (for the non-cyanobacterial clusters and the *hgl*-like islands) or the queried *hgl* island of *Anabaena* sp. PCC 7120 (for the most extended *hgl* islands of heterocytous cyanobacteria).

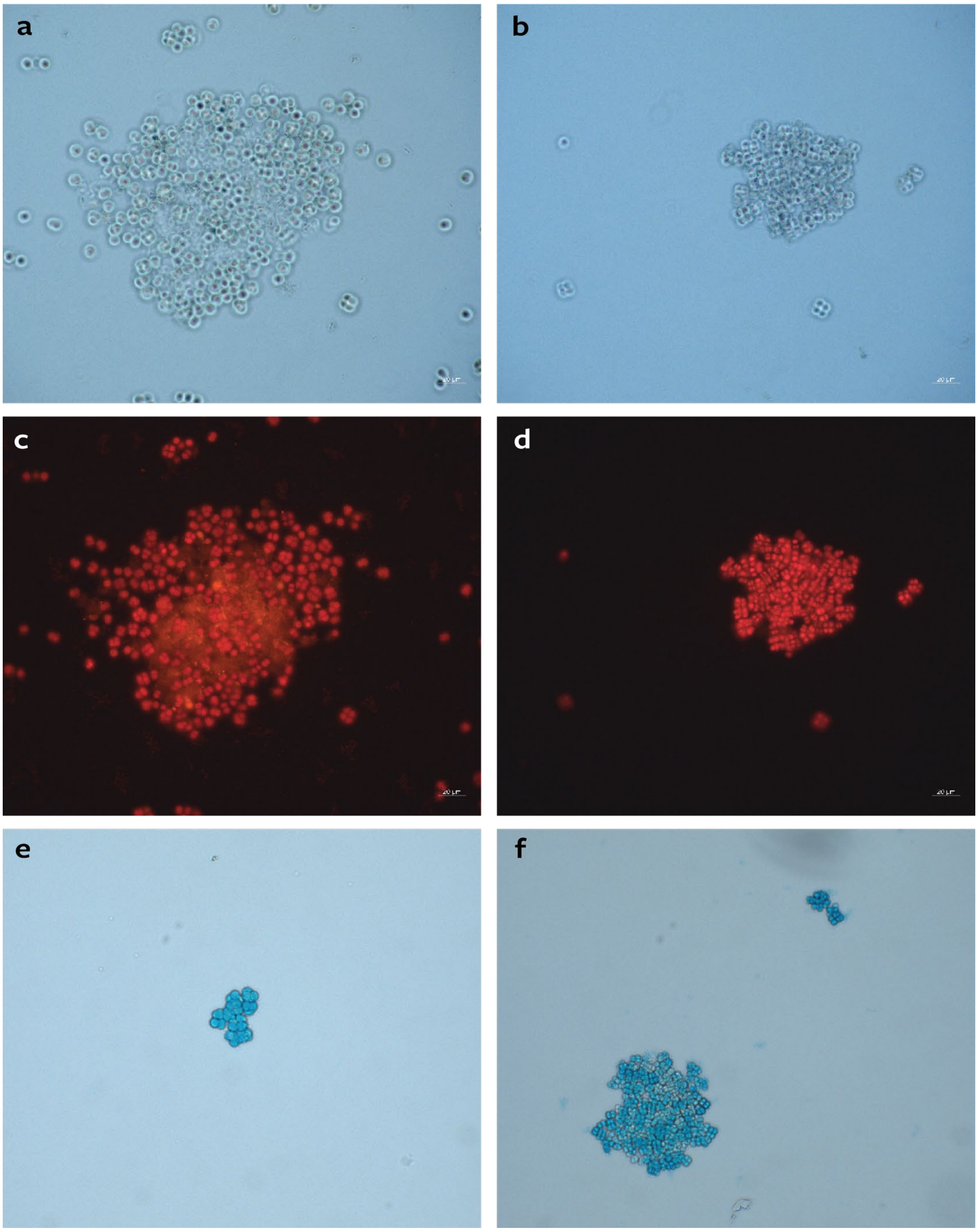

Supplementary Fig. 26. Microscopic analysis of *Gloeocapsopsis crepidinum* LEGE 06123 (a,c,e) and *Pleurocapsales* cyanobacterium LEGE 10410 (b,d,f) grown in nitrogen-replete media for 38 days (a,b) and 77 days (c-f). Panels a-d show lipids stained with Nile Red and analyzed using bright field (a-b) and epifluorescence microscopy (c-d). Panels e and f show polysaccharides stained with Alcian Blue.

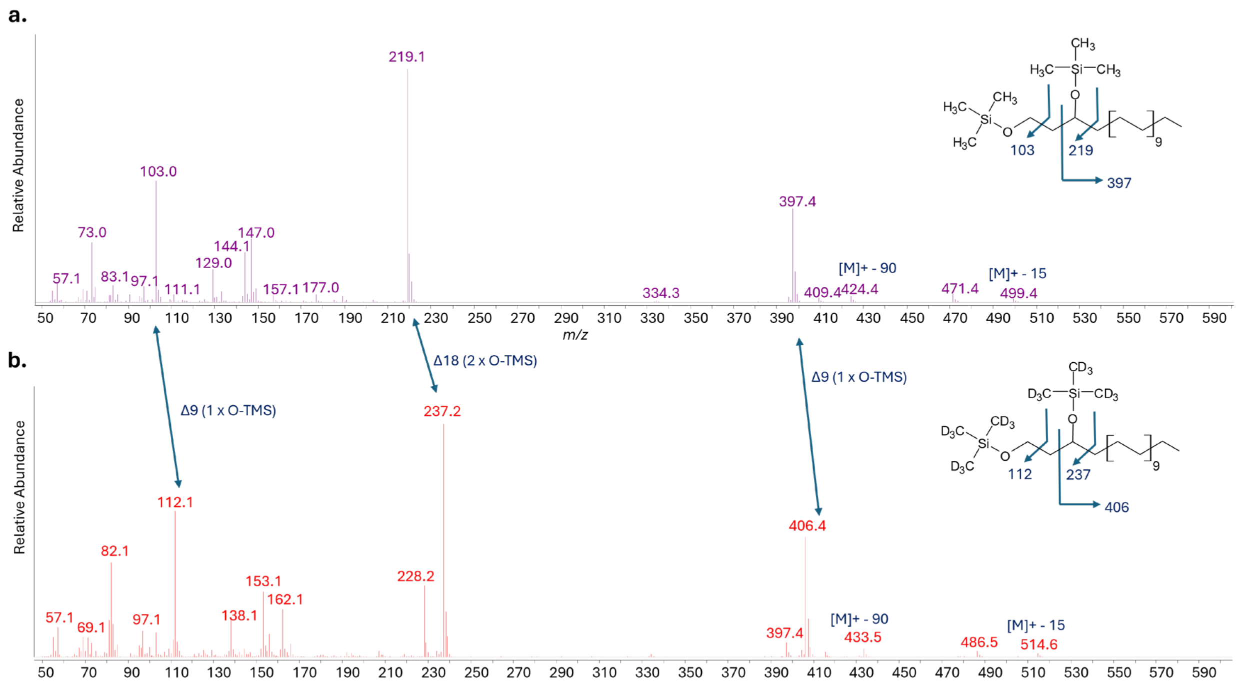

Supplementary Fig. 27. GC–MS spectra of compound eluting at 29.9 min after a, silylation with BSFTA and **b**, with deuterated BSTFA.

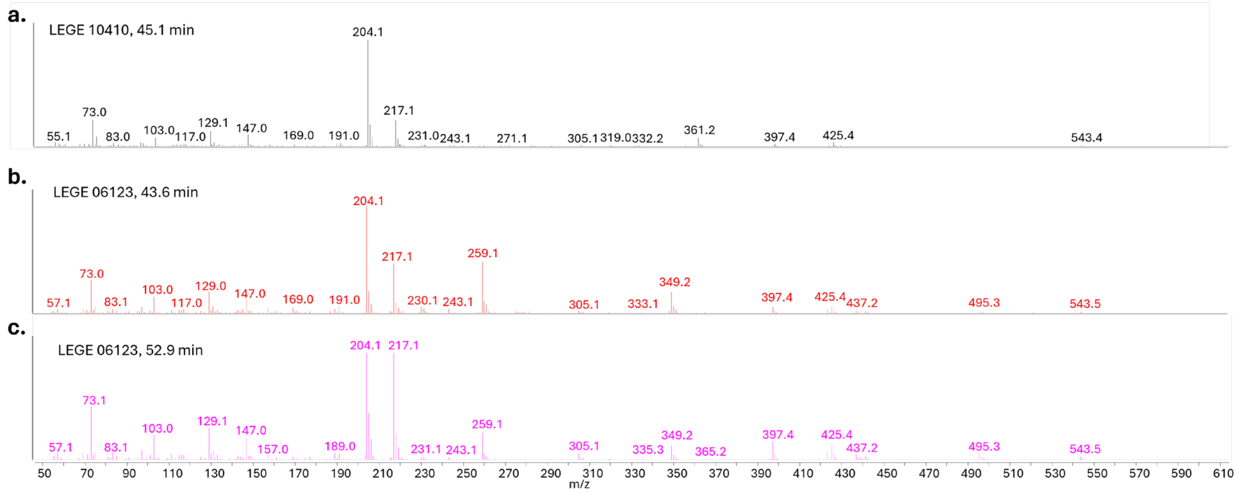

Supplementary Fig. 28. GC–MS spectra of tetracosane-1,3-diols eluting at a, 35.1 min with a hexose sugar headgroup, **b**, at 43.6 min and **c**, 52.9 min, both with a pentopyranose headgroup.

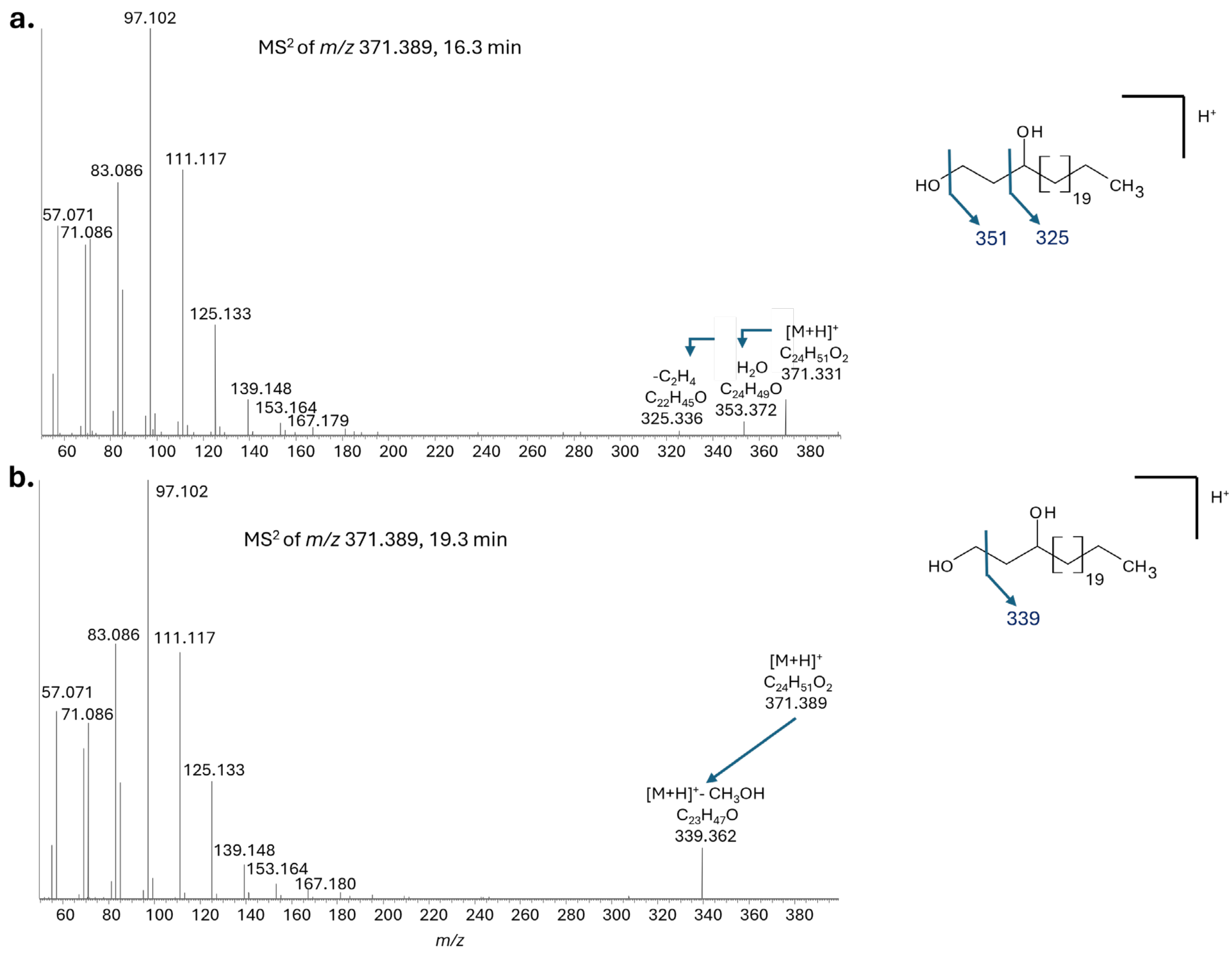

Supplementary Fig. 29. UHPLC-HRMS MS^2 spectra of tetracosane-1,3-diol ($[M+H]^+$ $C_{24}H_{51}O_2$, *m/z* 371.389) eluting at a, 16.3 min and **b**, 19.3 min.

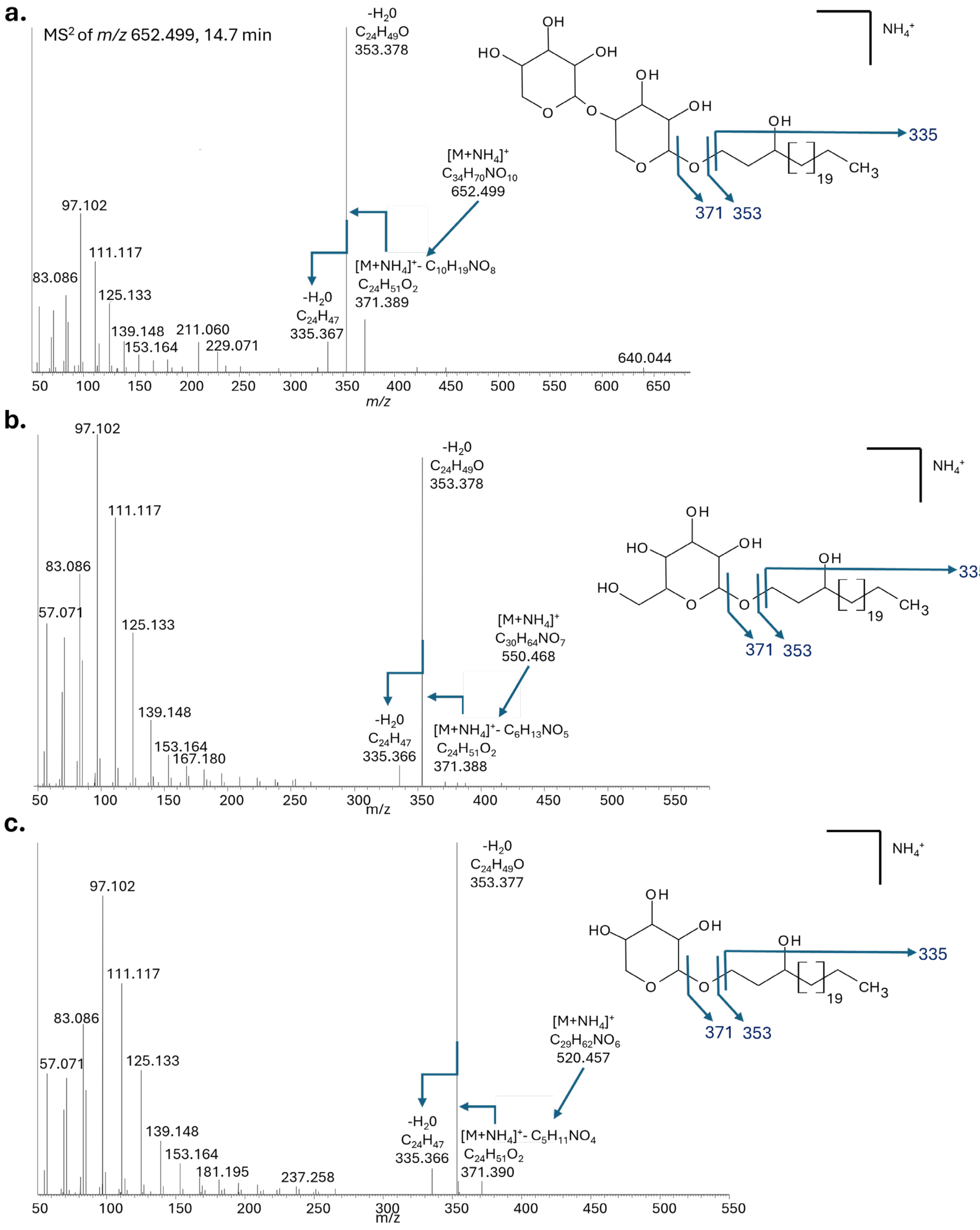

Supplementary Fig. 30. UHPLC-HRMS MS^2 spectra of larger components giving rise to fragment ions at *m/z* 371.389. a, compound eluting at 14.67 min ($[M+NH_4]^+$ at *m/z* 652.499) tentatively assigned as 1-(O-dipentopyranose)-3-tetracosanol. **b,** compound eluting at 14.81 min ($[M+NH_4]^+$ at *m/z* 550.468) tentatively assigned as a 1-(O-hexose)-3-tetracosaneol. **c,** compound eluting at 15.22 min giving rise to an $[M+NH_4]^+$ ion at m/z 520.457 tentatively assigned as a 1-(O-pentopyranose)-3-tetracosaneol.

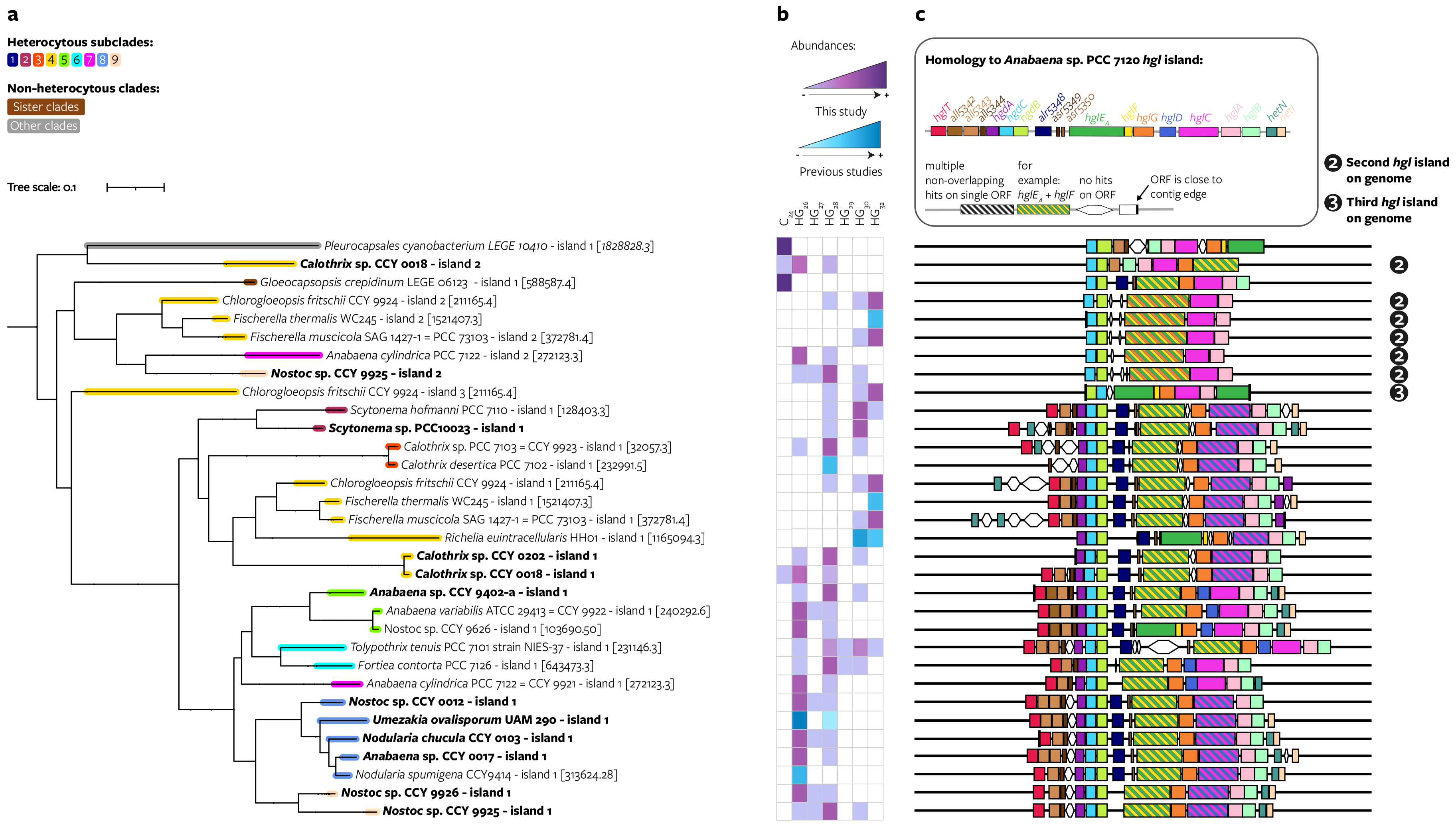

Supplementary Fig. 31. Distribution of 49 HGs and four HG analogs grouped according to alkyl chain length throughout cultures of heterocytous and non-heterocytous Cyanobacteriia. (Caption on next page).

Supplementary Fig. 31. Distribution of 49 HGs and four HG analogs grouped according to alkyl chain length throughout cultures of heterocytous and non-heterocytous *Cyanobacteriia*. **a**, Maximum likelihood phylogeny of the *hgl* island created using a concatenated alignment of homologous sequences of seven HG biosynthesis genes (*hgdCB* and *hglE*$_A$*FGCA*) that are often present on *hgl* islands including only genomes with a known HG lipid profile (pruned, see Online Methods). Genomes sequenced in this study are shown in bold. Some genomes contain multiple *hgl* islands and are in the tree multiple times. **b**, Heatmap of HG and HG analog abundances grouped according to their alkyl chain length. HG analogs (marked as C_{24} in the panel) are the sum of tetracosane-1,3-diol, 1-(O-hexose)-3-tetracosaneol, 1-(O-pentopyranose)-3-tetracosaneol, and 1-(O-dipentopyranose)-3-tetracosaneol. Relative abundances of HGs in *Calothrix* sp. CCY 0018 are scaled according to the total sum of HGs + HG analogs, which differs from other figures in which HG abundances in *Calothrix* sp. CCY 0018 are shown. Relative abundances are calculated in relation to the sum of all HGs produced by each strain. HG abundances obtained from either this study or previous studies are shown per row as purple or blue heatmaps, respectively. When data from both literature and this study were available only the data collected in this study are shown in the heatmap. Data originally represented in the literature as symbols were converted to percentages as follows; "+++", 90%; "++", 40%, "+", 15%; "tr.", 1%. Data used to generate this heatmap are found in Supplementary Table 10. **c**, Schematic representation of the genes present on the *hgl* island. When more than one island is present in the genome of the strain, the same HG abundances heatmap is shown for each island. 'First', 'second', and 'third' *hgl* islands are based on the presence of other islands on the genome, where the 'first' *hgl* island (not marked) is the most extended island in terms of number of *Anabaena* sp. PCC 7120 genes with homologs on the island, the second *hgl* island (marked with a 2) the second-most extended island, and the third island (marked with 3) the third-most extended island. ORF, open reading frame; contig, contiguous sequence.

Supplementary Data captions

Supplementary Data 1. Selected cyanobacterial genomes from the PATRIC genome database (now part of the BV-BRC database). Files called 'selected_Cyanogenomes.genome_*.20220430.txt' are sourced from the PATRIC File Transfer Protocol server (ftp.patricbrc.org). 'gtdbtk.bac120.summary.tsv' is the GTDB-Tk output file, and 'qa.summary_extended.txt' the CheckM output file.

Supplementary Data 2. HG biosynthetic gene clusters in selected PATRIC genomes and 14 newly sequenced genomes. The file 'islands_on_contigs.3_ORFs_in_between.expanded_island_with_nucleotide_positions.txt' contains the location of all hits to *Anabaena* sp. PCC 7120 HG biosynthesis genes. ORFs were predicted with Prodigal. The structure of a contig is as follows: "genome | contig". The structure of a hit is as follows: "ORF number on contig | query (*e*-value; bit-score; start of alignment in query; end of alignment in query; query coverage per subject; start of alignment in subject; end of alignment in subject; subject coverage) [nucleotide position on contig start; nucleotide position on contig end; direction]". Non-overlapping hits on the same ORF (see Online Methods) are connected with '&&&' characters. An asterisk ('*') indicates that the hit is located at most three ORFs from a contig edge. Clusters of hits that are at most three open reading frames (ORFs) apart are connected with '~~~' characters. The file 'Supplementary_table.script_1.txt' contains a summary of all identified *hgl* islands (i.e. clusters containing at least seven unique HG biosynthesis gene hits).

Supplementary Data 3. HG biosynthetic gene clusters in 255,388 prokaryotic genomes from the PATRIC genome database (now part of the BV-BRC database). The file called 'PATRIC_20230120.selection_c50_c10.txt' contains information on the selected PATRIC genomes based on data sourced from the PATRIC File Transfer Protocol server (ftp.patricbrc.org). The file 'all_tree_of_life_genomes.islands_on_contigs.3_ORFs_in_between.expanded_island_with_nucleotide_positions.txt' contains the location of all hits to *Anabaena* sp. PCC 7120 HG biosynthesis genes. ORFs were predicted with Prodigal. The structure of a contig is as follows: "genome | contig". The structure of a hit is as follows: "ORF number on contig | query (*e*-value; bit-score; start of alignment in query; end of alignment in query; query coverage per subject; start of alignment in subject; end of alignment in subject; subject coverage) [nucleotide position on contig start; nucleotide position on contig end; direction]". Non-overlapping hits on the same ORF (see Online Methods) are connected with '&&&' characters. An asterisk ('*') indicates that the hit is located at most three ORFs from a

contig edge. Clusters of hits that are at most three open reading frames (ORFs) apart are connected with '~~~' characters.

Supplementary Data 4. Phylogeny of representative cyanobacterial genomes based on a core gene superalignment. The folder contains the files used to generate Fig. 2a. The directory 'IQ-TREE' contains the tree file and iTOL annotation files. The file 'dRep.representative_to_cluster.txt' contains the dRep clusters. Note that the manually defined subclades in the iTOL annotation file 'iTOL_annotation.manually_defined_clades.DATASET_STYLE.txt' have a different numbering from the paper: subclades 0 and 1 are the 'heterocytous sister clades', and subclades 2-10 in the annotation file are heterocytous subclades 1-9 in the paper, respectively.

Supplementary Data 5. Lipid data files. The folder contains all the UHPLC-HRMS^n (Orbitrap) datafiles used in this study. The directory 'CCY strains' includes 24 heterocytous cyanobacterial cultures corresponding to 23 strains grown in nitrogen-deficient media, the resulting data are shown in Supplementary Table 10. Directory 'HglT mutant' contains the datafiles used to generate Supplementary Table 15. The directory 'LEGE strains' includes the UHPLC-HRMS^n (Orbitrap) and GC-MS datafiles corresponding to eight cultures of two non-heterocytous strains grown in media with and without nitrogen for 38 to 77 days, the resulting data are shown in Supplementary Tables 10, 17 and 18.

Supplementary Data 6. Plasmid maps. GenBank and FASTA files of plasmids generated in this study. 'HglT deletion' directory contains the genomic region surrounding *hglT* in the *wild-type* strain and after deletion used to generate Supplementary Fig. 15. pAM5404 is shown in Supplementary Fig. 16 and p(A)RP0XX are shown in Supplementary Fig. 17.

Supplementary Data 7. Phylogenies of seven *hgl* island genes and of a concatenated alignment of these genes. The folder contains the files used to generate Supplementary Fig. 18 (in the directory 'gene_trees_hgl_islands'), and Fig. 4 and related figures (in the directory 'gene_trees_hgl_islands_4'. The directories contain the alignments and trimmed alignments, IQ-TREE output files, and iTOL annotation files. The file 'gene_trees_hgl_islands/analysis_individual_gene_trees/explore_individual_gene_clusters.ipynb' contains the code to identify the five *hgl* islands that contain genes with incongruent evolutionary histories.

Supplementary Data 8. Phylogeny of *hglE$_A$* homologs. The folder contains the files used to generate Supplementary Fig. 24 and related figures. The file 'selected_hglE_hits.txt' contains the selected *hglE$_A$* hits and the genomic cluster on which they are located. The folder contains the alignment and trimmed alignment, IQ-TREE output files, and iTOL annotation files.

Supplementary Information References

1. S. Ehira, M. Ohmori, N. Sato, Genome-wide Expression Analysis of the Responses to Nitrogen Deprivation in the Heterocyst-forming Cyanobacterium *Anabaena* sp. Strain PCC 7120. *DNA Research* **10**, 97–113 (2003).

2. Q. Fan, *et al.*, Clustered genes required for synthesis and deposition of envelope glycolipids in *Anabaena* sp. strain PCC 7120: Synthesis of heterocyst envelope glycolipid layer. *Molecular Microbiology* **58**, 227–243 (2005).

3. K. Awai, C. P. Wolk, Identification of the glycosyl transferase required for synthesis of the principal glycolipid characteristic of heterocysts of *Anabaena* sp. strain PCC 7120. *FEMS Microbiol Lett* **266**, 98–102 (2007).

4. H. S. M. Halimatul, S. Ehira, K. Awai, Fatty alcohols can complement functions of heterocyst specific glycolipids in *Anabaena* sp. PCC 7120. *Biochemical and Biophysical Research Communications* **450**, 178–183 (2014).

5. B. Kultschar, C. Llewellyn, B. Kultschar, C. Llewellyn, "Secondary Metabolites in Cyanobacteria" in *Secondary Metabolites - Sources and Applications*, (IntechOpen, 2018).

6. Y. Jeong, *et al.*, Current Status and Future Strategies to Increase Secondary Metabolite Production from Cyanobacteria. *Microorganisms* **8**, 1849 (2020).

7. P. M. Shih, *et al.*, Improving the coverage of the cyanobacterial phylum using diversity-driven genome sequencing. *Proceedings of the National Academy of Sciences* **110**, 1053–1058 (2013).

8. M.-Y. Chen, *et al.*, Phylogenomics Uncovers Evolutionary Trajectory of Nitrogen Fixation in Cyanobacteria. *Molecular Biology and Evolution* **39**, msac171 (2022).

9. A. A. Esteves-Ferreira, *et al.*, Cyanobacterial nitrogenases: phylogenetic diversity, regulation and functional predictions. *Genet Mol Biol* **40**, 261–275 (2017).

10. E. R. Moody, *et al.*, An estimate of the deepest branches of the tree of life from ancient vertically evolving genes. *eLife* **11**, e66695 (2022).

11. R. M. Soo, *et al.*, An Expanded Genomic Representation of the Phylum Cyanobacteria. *Genome Biology and Evolution* **6**, 1031–1045 (2014).

12. S. C. Di Rienzi, *et al.*, The human gut and groundwater harbor non-photosynthetic bacteria belonging to a new candidate phylum sibling to Cyanobacteria. *eLife* **2**, e01102 (2013).

13. D. Shvarev, C. N. Nishi, L. Wörmer, I. Maldener, The ABC Transporter Components HgdB and HgdC are Important for Glycolipid Layer Composition and Function of Heterocysts in *Anabaena* sp. PCC 7120. *Life (Basel)* **8**, 26 (2018).

14. T. Saito, K. Awai, A polyketide synthase HglEA, but not HglE2, synthesizes heterocyst specific glycolipids in *Anabaena* sp. PCC 7120. *J Gen Appl Microbiol* **66**, 99–105 (2020).

15. C. C. Bauer, *et al.*, Suppression of heterocyst differentiation in *Anabaena* PCC 7120 by a cosmid carrying wild-type genes encoding enzymes for fatty acid synthesis. *FEMS Microbiology Letters* **151**, 23–30 (1997).

16. T. A. Black, C. P. Wolk, Analysis of a Het- mutation in *Anabaena* sp. strain PCC 7120 implicates a secondary metabolite in the regulation of heterocyst spacing. *J Bacteriol* **176**, 2282–2292 (1994).

17. R. Garg, I. Maldener, The Dual Role of the Glycolipid Envelope in Different Cell Types of the Multicellular Cyanobacterium *Anabaena variabilis* ATCC 29413. *Frontiers in Microbiology* **12** (2021).

18. S. M. Callahan, W. J. Buikema, The role of HetN in maintenance of the heterocyst pattern in *Anabaena* sp. PCC 7120. *Molecular Microbiology* **40**, 941–950 (2001).

19. J. N. Copp, B. A. Neilan, The Phosphopantetheinyl Transferase Superfamily: Phylogenetic Analysis and Functional Implications in Cyanobacteria. *Appl Environ Microbiol* **72**, 2298–2305 (2006).

20. V. A. C. Abreu, *et al.*, Genomic and Genotypic Characterization of *Cylindrospermopsis raciborskii*: Toward an Intraspecific Phylogenetic Evaluation by Comparative Genomics. *Front Microbiol* **9**, 306 (2018).

21. C. P. Wolk, A. Ernst, J. Elhai, "Heterocyst Metabolism and Development" in *The Molecular Biology of Cyanobacteria*, Advances in Photosynthesis., D. A. Bryant, Ed. (Springer Netherlands, 1994), pp. 769–823.

22. L. Cardemil, C. P. Wolk, The polysaccharides from heterocyst and spore envelopes of a blue-green alga. Structure of the basic repeating unit. *Journal of Biological Chemistry* **254**, 736–741 (1979).

23. A. Soriente, *et al.*, Heterocyst glycolipids of the cyanobacterium *Cyanospira rippkae*. *Phytochemistry* **33**, 393–396 (1993).

24. T. Bauersachs, *et al.*, Rapid analysis of long-chain glycolipids in heterocystous cyanobacteria using high-performance liquid chromatography coupled to electrospray ionization tandem mass spectrometry. *Rapid Commun. Mass Spectrom.* **23**, 1387–1394 (2009).

25. T. Bauersachs, M. Gugger, L. Schwark, Heterocyte glycolipid diketones: A novel type of biomarker in the N_2-fixing heterocytous cyanobacterium *Microchaete* sp. *Organic Geochemistry* **141**, 103976 (2020).

26. L. Wörmer, S. Cires, D. Velazquez, A. Quesada, K.-U. Hinrichs, Cyanobacterial heterocyst glycolipids in cultures and environmental samples: Diversity and biomarker potential. *Limnol. Oceanogr.* **57**, 1775–1788 (2012).

27. T. Bauersachs, *et al.*, Distribution of long chain heterocyst glycolipids in cultures of the thermophilic cyanobacterium *Mastigocladus laminosus* and a hot spring microbial mat. *Organic Geochemistry* **56**, 19–24 (2013).

28. T. Bauersachs, O. Mudimu, R. Schulz, L. Schwark, Distribution of long chain heterocyst glycolipids in N_2 fixing cyanobacteria of the order *Stigonematales*. *Phytochemistry* **98**, 145–150 (2014).

29. S. Schouten, *et al.*, Endosymbiotic heterocystous cyanobacteria synthesize different heterocyst glycolipids than free-living heterocystous cyanobacteria. *Phytochemistry* **85**, 115–121 (2013).

30. N. J. Bale, *et al.*, Long chain glycolipids with pentose head groups as biomarkers for marine endosymbiotic heterocystous cyanobacteria. *Organic Geochemistry* **81**, 1–7 (2015).

31. N. J. Bale, *et al.*, A novel heterocyst glycolipid detected in a pelagic N_2-fixing cyanobacterium of the genus *Calothrix*. *Organic Geochemistry* **123**, 44–47 (2018).

32. T. Bauersachs, *et al.*, Distribution of heterocyst glycolipids in cyanobacteria. *Phytochemistry* **70**, 2034–2039 (2009).

33. A. Gambacorta, *et al.*, The heterocyst glycolipids of cyanobacteria. 5. Heterocyst glycolipids from five nitrogen-fixing cyanobacteria. *Gazz. Chim. Ital.* **126**, 653–656 (1996).

34. L. Krall, J. Huege, G. Catchpole, D. Steinhauser, L. Willmitzer, Assessment of sampling strategies for gas chromatography–mass spectrometry (GC–MS) based metabolomics of cyanobacteria. *Journal of Chromatography B* **877**, 2952–2960 (2009).

35. J. Komarek, J. Kastovsky, J. Mares, J. Johansen, Taxonomic classification of cyanoprokaryotes (cyanobacterial genera) 2014, using a polyphasic approach. *Preslia* **86**, 295–335 (2014).

36. R. W. Castenholz, *et al.*, “Phylum BX. Cyanobacteria” in *Bergey’s Manual of Systematic Bacteriology: Volume One : The Archaea and the Deeply Branching and Phototrophic Bacteria*, D. R. Boone, R. W. Castenholz, G. M. Garrity, Eds. (Springer, 2001), pp. 473–599.

37. T. Bauersachs, H. M. Talbot, F. Sidgwick, K. Sivonen, L. Schwark, Lipid biomarker signatures as tracers for harmful cyanobacterial blooms in the Baltic Sea. *PLoS ONE* **12**, e0186360 (2017).

38. T. Hauer, M. Bohunická, J. R. Johansen, J. Mareš, E. Berrendero-Gomez, Reassessment of the cyanobacterial family *Microchaetaceae* and establishment of new families *Tolypothrichaceae* and *Godleyaceae*. *Journal of Phycology* **50**, 1089–1100 (2014).

39. E. E. Allen, D. H. Bartlett, Structure and regulation of the omega-3 polyunsaturated fatty acid synthase genes from the deep-sea bacterium *Photobacterium profundum* strain SS9. *Microbiology* **148**, 1903–1913 (2002).

40. M. N. Allemann, C. N. Shulse, E. E. Allen, Linkage of Marine Bacterial Polyunsaturated Fatty Acid and Long-Chain Hydrocarbon Biosynthesis. *Front. Microbiol.* **10** (2019).

41. M. Tanaka, *et al.*, Isolation of clustered genes that are notably homologous to the eicosapentaenoic acid biosynthesis gene cluster from the docosahexaenoic acid-producing bacterium *Vibrio marinus* strain MP-1. *Biotechnology Letters* **21**, 939–945 (1999).

42. H. Okuyama, Y. Orikasa, T. Nishida, K. Watanabe, N. Morita, Bacterial Genes Responsible for the Biosynthesis of Eicosapentaenoic and Docosahexaenoic Acids and Their Heterologous Expression. *Applied and Environmental Microbiology* **73**, 665–670 (2007).

43. C. N. Shulse, E. E. Allen, Widespread Occurrence of Secondary Lipid Biosynthesis Potential in Microbial Lineages. *PLOS ONE* **6**, e20146 (2011).

44. T. Nishida, *et al.*, Escherichia coli engineered to produce eicosapentaenoic acid becomes resistant against oxidative damages. *FEBS Letters* **580**, 2731–2735 (2006).

45. T. Nishida, R. Hori, N. Morita, H. Okuyama, Membrane eicosapentaenoic acid is involved in the hydrophobicity of bacterial cells and affects the entry of hydrophilic and hydrophobic compounds. *FEMS Microbiology Letters* **306**, 91–96 (2010).

46. H. Okuyama, Y. Orikasa, T. Nishida, Significance of Antioxidative Functions of Eicosapentaenoic and Docosahexaenoic Acids in Marine Microorganisms. *Applied and Environmental Microbiology* **74**, 570–574 (2008).

47. A. Tilay, U. Annapure, Novel Simplified and Rapid Method for Screening and Isolation of Polyunsaturated Fatty Acids Producing Marine Bacteria. *Biotechnology Research International* **2012**, 542721 (2012).

48. K. Yoshida, *et al.*, Bacterial Long-Chain Polyunsaturated Fatty Acids: Their Biosynthetic Genes, Functions, and Practical Use. *Marine Drugs* **14**, 94 (2016).

49. S. Hosoya, V. Arunpairojana, C. Suwannachart, A. Kanjana-Opas, A. Yokota, *Aureispira marina* gen. nov., sp. nov., a gliding, arachidonic acid-containing bacterium isolated from the southern coastline of Thailand. *International Journal of Systematic and Evolutionary Microbiology* **56**, 2931–2935 (2006).

50. S. Feng, S. M. Powell, R. Wilson, J. P. Bowman, Extensive Gene Acquisition in the Extremely Psychrophilic Bacterial Species *Psychroflexus torquis* and the Link to Sea-Ice Ecosystem Specialism. *Genome Biology and Evolution* **6**, 133–148 (2014).

51. N. Funa, H. Ozawa, A. Hirata, S. Horinouchi, Phenolic lipid synthesis by type III polyketide synthases is essential for cyst formation in *Azotobacter vinelandii*. *Proceedings of the National Academy of Sciences* **103**, 6356–6361 (2006).

52. F. A. B. von Meijenfeldt, K. Arkhipova, D. D. Cambuy, F. H. Coutinho, B. E. Dutilh, Robust taxonomic classification of uncharted microbial sequences and bins with CAT and BAT. *Genome Biol* **20**, 217 (2019).

53. J.-L. Shang, *et al.*, Genomic and transcriptomic insights into the survival of the subaerial cyanobacterium *Nostoc flagelliforme* in arid and exposed habitats. *Environmental Microbiology* **21**, 845–863 (2019).

54. P. M. Medeiros, B. R. T. Simoneit, Analysis of sugars in environmental samples by gas chromatography–mass spectrometry. *Journal of Chromatography A* **1141**, 271–278 (2007).

55. R. Rippka, J. Deruelles, J. B. Waterbury, M. Herdman, R. Y. Stanier, Generic Assignments, Strain Histories and Properties of Pure Cultures of Cyanobacteria. *Microbiology,* **111**, 1–61 (1979).

56. A. M. Bolger, M. Lohse, B. Usadel, Trimmomatic: a flexible trimmer for Illumina sequence data. *Bioinformatics* **30**, 2114–2120 (2014).

57. M. Martin, Cutadapt removes adapter sequences from high-throughput sequencing reads. *EMBnet.journal* **17**, 10–12 (2011).

58. S. Andrews, FastQC: a quality control tool for high throughput sequence data. (2010). Available at: https://www.bioinformatics.babraham.ac.uk/projects/fastqc/ [Accessed 18 January 2023].

59. D. Meleshko, *et al.*, BiosyntheticSPAdes: Reconstructing Biosynthetic Gene Clusters From Assembly Graphs. *Genome Res.* gr.243477.118 (2019).

60. R. Challis, E. Richards, J. Rajan, G. Cochrane, M. Blaxter, BlobToolKit – Interactive Quality Assessment of Genome Assemblies. *G3 Genes|Genomes|Genetics* **10**, 1361–1374 (2020).

61. S. F. Altschul, W. Gish, W. Miller, E. W. Myers, D. J. Lipman, Basic local alignment search tool. *J Mol Biol* **215**, 403–410 (1990).

62. C. Camacho, *et al.*, BLAST+: architecture and applications. *BMC Bioinformatics* **10**, 421 (2009).

63. B. Buchfink, C. Xie, D. H. Huson, Fast and sensitive protein alignment using DIAMOND. *Nat Methods* **12**, 59–60 (2015).

64. The UniProt Consortium, UniProt: the universal protein knowledgebase in 2021. *Nucleic Acids Research* **49**, D480–D489 (2021).

65. H. Li, Minimap2: pairwise alignment for nucleotide sequences. *Bioinformatics* **34**, 3094–3100 (2018).

66. M. Manni, M. R. Berkeley, M. Seppey, F. A. Simão, E. M. Zdobnov, BUSCO Update: Novel and Streamlined Workflows along with Broader and Deeper Phylogenetic Coverage for Scoring of Eukaryotic, Prokaryotic, and Viral Genomes. *Molecular Biology and Evolution* **38**, 4647–4654 (2021).

67. J. J. Davis, *et al.*, The PATRIC Bioinformatics Resource Center: expanding data and analysis capabilities. *Nucleic Acids Research* **48**, D606–D612 (2020).

68. R. D. Olson, *et al.*, Introducing the Bacterial and Viral Bioinformatics Resource Center (BV-BRC): a resource combining PATRIC, IRD and ViPR. *Nucleic Acids Res* **51**, D678–D689 (2023).

69. P.-A. Chaumeil, A. J. Mussig, P. Hugenholtz, D. H. Parks, GTDB-Tk v2: memory friendly classification with the genome taxonomy database. *Bioinformatics* **38**, 5315–5316 (2022).

70. D. H. Parks, *et al.*, GTDB: an ongoing census of bacterial and archaeal diversity through a phylogenetically consistent, rank normalized and complete genome-based taxonomy. *Nucleic Acids Research* **50**, D785–D794 (2022).

71. D. H. Parks, M. Imelfort, C. T. Skennerton, P. Hugenholtz, G. W. Tyson, CheckM: assessing the quality of microbial genomes recovered from isolates, single cells, and metagenomes. *Genome Res.* **25**, 1043–1055 (2015).

72. D. Hyatt, *et al.*, Prodigal: prokaryotic gene recognition and translation initiation site identification. *BMC Bioinformatics* **11**, 119 (2010).

73. T. Kaneko, Complete Genomic Sequence of the Filamentous Nitrogen-fixing Cyanobacterium *Anabaena* sp. Strain PCC 7120. *DNA Research* **8**, 205–213 (2001).

74. E. W. Sayers, *et al.*, GenBank. *Nucleic Acids Research* **48**, D84–D86 (2020).

75. H. Zhang, *et al.*, dbCAN2: a meta server for automated carbohydrate-active enzyme annotation. *Nucleic Acids Res* **46**, W95–W101 (2018).

76. R. M. Bowers, *et al.*, Minimum information about a single amplified genome (MISAG) and a metagenome-assembled genome (MIMAG) of bacteria and archaea. *Nat Biotechnol* **35**, 725–731 (2017).

77. M. R. Olm, C. T. Brown, B. Brooks, J. F. Banfield, dRep: a tool for fast and accurate genomic comparisons that enables improved genome recovery from metagenomes through de-replication. *ISME J* **11**, 2864–2868 (2017).

78. M. R. Olm, *et al.*, Consistent Metagenome-Derived Metrics Verify and Delineate Bacterial Species Boundaries. *mSystems* **5**, 10.1128/msystems.00731-19 (2020).

79. K. Katoh, D. M. Standley, MAFFT Multiple Sequence Alignment Software Version 7: Improvements in Performance and Usability. *Molecular Biology and Evolution* **30**, 772–780 (2013).

80. S. R. Eddy, Accelerated Profile HMM Searches. *PLoS Comput Biol* **7**, e1002195 (2011).

81. S. Capella-Gutiérrez, J. M. Silla-Martínez, T. Gabaldón, trimAl: a tool for automated alignment trimming in large-scale phylogenetic analyses. *Bioinformatics* **25**, 1972–1973 (2009).

82. B. Q. Minh, *et al.*, IQ-TREE 2: New Models and Efficient Methods for Phylogenetic Inference in the Genomic Era. *Mol Biol Evol* **37**, 1530–1534 (2020).

83. D. T. Hoang, O. Chernomor, A. von Haeseler, B. Q. Minh, L. S. Vinh, UFBoot2: Improving the Ultrafast Bootstrap Approximation. *Molecular Biology and Evolution* **35**, 518–522 (2018).

84. S. Kalyaanamoorthy, B. Q. Minh, T. K. F. Wong, A. von Haeseler, L. S. Jermiin, ModelFinder: fast model selection for accurate phylogenetic estimates. *Nat Methods* **14**, 587–589 (2017).

85. I. Letunic, P. Bork, Interactive Tree Of Life (iTOL) v5: an online tool for phylogenetic tree display and annotation. *Nucleic Acids Research* **49**, W293–W296 (2021).

86. E. W. Sayers, *et al.*, Database resources of the national center for biotechnology information. *Nucleic Acids Research* **50**, D20–D26 (2022).

87. D. Hyatt, P. F. LoCascio, L. J. Hauser, E. C. Uberbacher, Gene and translation initiation site prediction in metagenomic sequences. *Bioinformatics* **28**, 2223–2230 (2012).

88. B. Buchfink, K. Reuter, H.-G. Drost, Sensitive protein alignments at tree-of-life scale using DIAMOND. *Nat Methods* **18**, 366–368 (2021).

89. I.-M. A. Chen, *et al.*, The IMG/M data management and analysis system v.6.0: new tools and advanced capabilities. *Nucleic Acids Research* **49**, D751–D763 (2021).

90. R. K. Aziz, *et al.*, The RAST Server: Rapid Annotations using Subsystems Technology. *BMC Genomics* **9**, 75 (2008).

91. J. Elhai, C. P. Wolk, Developmental regulation and spatial pattern of expression of the structural genes for nitrogenase in the cyanobacterium *Anabaena*. *EMBO J* **9**, 3379–3388 (1990).

92. B. Bishé, A. Taton, J. W. Golden, Modification of RSF1010-Based Broad-Host-Range Plasmids for Improved Conjugation and Cyanobacterial Bioprospecting.

93. A. Taton, *et al.*, Broad-host-range vector system for synthetic biology and biotechnology in cyanobacteria. *Nucleic Acids Research* **42**, e136–e136 (2014).

94. J. Elhai, C. P. Wolk, "[83] Conjugal transfer of DNA to cyanobacteria" in *Methods in Enzymology*, Cyanobacteria., (Academic Press, 1988), pp. 747–754.

95. T. Thiel, C. Peter Wolk, "[13] Conjugal transfer of plasmids to cyanobacteria" in *Methods in Enzymology*, Recombinant DNA Part D., (Academic Press, 1987), pp. 232–243.

96. J. Elhai, A. Vepritskiy, A. M. Muro-Pastor, E. Flores, C. P. Wolk, Reduction of conjugal transfer efficiency by three restriction activities of *Anabaena* sp. strain PCC 7120. *Journal of Bacteriology* **179**, 1998–2005 (1997).

97. N. J. Bale, *et al.*, C_5 glycolipids of heterocystous cyanobacteria track symbiont abundance in the diatom *Hemiaulus hauckii* across the tropical North Atlantic. *Biogeosciences* **15**, 1229–1241 (2018).

98. N. J. Bale, *et al.*, Lipidomics of Environmental Microbial Communities. I: Visualization of Component Distributions Using Untargeted Analysis of High-Resolution Mass Spectrometry Data. *Frontiers in Microbiology* **12** (2021).

99. K. Awai, S. Lechno-Yossef, C. P. Wolk, "Heterocyst Envelope Glycolipids" in *Lipids in Photosynthesis: Essential and Regulatory Functions*, Advances in Photosynthesis and Respiration., H. Wada, N. Murata, Eds. (Springer Netherlands, 2009), pp. 179–202.

100. A. R. Wattam, *et al.*, Improvements to PATRIC, the all-bacterial Bioinformatics Database and Analysis Resource Center. *Nucleic Acids Res* **45**, D535–D542 (2017).

101. T. Bauersachs, *et al.*, Heterocyte glycolipids indicate polyphyly of stigonematalean cyanobacteria. *Phytochemistry* **166**, 112059 (2019).

102. A. Gambacorta, E. Pagnotta, I. Romano, G. Sodano, A. Trincone, Heterocyst glycolipids from nitrogen-fixing cyanobacteria other than Nostocaceae. *Phytochemistry* **48**, 801–805 (1998).

103. T. Bauersachs, *et al.*, Fossilized glycolipids reveal past oceanic N_2 fixation by heterocystous cyanobacteria. *Proceedings of the National Academy of Sciences* **107**, 19190–19194 (2010).
